# Biosynthesis of silver nanoparticles by *Fusarium scirpi* and its potential as antimicrobial agent against uropathogenic *Escherichia coli* biofilms

**DOI:** 10.1371/journal.pone.0230275

**Published:** 2020-03-12

**Authors:** Candelario Rodríguez-Serrano, Jesús Guzmán-Moreno, Carlos Ángeles-Chávez, Vicente Rodríguez-González, José Juan Ortega-Sigala, Rosa María Ramírez-Santoyo, Luz Elena Vidales-Rodríguez

**Affiliations:** 1 Unidad Académica de Ciencias Biológicas, Universidad Autónoma de Zacatecas “Francisco García Salinas”, Zacatecas, Zacatecas, México; 2 Gerencia de Desarrollo de Materiales y Productos Químicos, Instituto Mexicano del Petróleo, Ciudad de México, México; 3 División de Materiales Avanzados, Instituto Potosino de Investigación Científica y Tecnológica A. C., San Luis Potosí, San Luis Potosí, México; 4 Unidad Académica de Física, Universidad Autónoma de Zacatecas “Francisco García Salinas”, Zacatecas, México; Institute of Materials Science, GERMANY

## Abstract

The ability of Uropathogenic *Escherichia coli* (UPEC) to form biofilms, can be considered an important factor that contributes to the prevalence of Urinary Tract Infections (UTIs) due to the inaccessibility of the antibiotics into the highly complex structure of the biofilm. Moreover, with the appearance of antibiotic multiresistant UPEC strains, the alternatives of treatment of UTIs are less. Silver nanoparticles (AgNPs) can be useful in the treatment of the UPEC infections due to its physicochemical properties that confer them antibacterial activity against both planktonic and biofilm structured cells. A diversity of biological methods for synthesis of AgNPs with antimicrobial activity has been widely investigated during the last decades, between these methods; the fungal-biosynthesis of AgNPs highlights as an ecofriendly, scalable and low cost method. In this study, biogenic AgNPs were synthesized with extracellular metabolites secreted by the soil fungal strain *Fusarium scirpi* (Ag0.5–5) by an ecofriendly, simple and efficient method. The antimicrobial activity of the biosynthesized AgNPs against UPEC was evaluated. The Minimal Inhibitory Concentration (MIC) of biogenic AgNPs over planktonic UPEC cells was 25 mg/mL, whereas a sub-MIC concentration (7.5 mg/L) was sufficient to inhibit the UPEC-biofilm formation about a 97%, or produce the disruption of an 80% of mature UPEC-biofilms demonstrating the potential of fungal-derived AgNPs to prevent UPEC infections.

## Introduction

Uropthogenic *Escherichia coli* (UPEC) is the main pathogen associated with Urinary Tract Infections (UTIs) [1], its pathogenicity resides on several virulence factors codified in genomic Pathogenicity Islands (PAI) that includes adhesins, toxins, flagella, surface polysaccharides and iron-acquisition systems [2]. The ability of UPEC to form biofilms is an important factor that contributes to the prevalence of UTIs since it confers some advantages for concentration of nutrients, resistance to drastic fluctuations of environmental conditions and protection against the effects of antibiotics between others [3]. Between a wide number of genes implicated in biofilm formation [4,5]; those related to the phenotypic expression of some adhesins such as Type I fimbriae (*fim* operon encoded), curli fimbriae (*csg* operon encoded), P fimbriae (*pap* operon encoded) and Antigen 43 factor (*flu* gene encoded); are crucial at initial steps of the UPEC-biofilm formation [1,6].

The persistence of UPEC within the bladder, regardless of antibiotic treatments, and the increased number of UPEC-resistant isolates to several antibiotics [7]; leads to a reduction of the alternatives for the UTIs treatment. Thus, it could represents a public health problem that can be coped with the development of new or more effective antibiotics, or antimicrobial agents such as the antimicrobial peptides, synthetic chemical compounds, or inorganic nanoparticles (NPs) [8]. The antimicrobial properties of a wide variety of NPs has previously reported [9]. In particular; the use of silver nanoparticles (AgNPs) as an efficient microbicide agent highlights between a wide number of applications such as electronic, bio-sensing, clothing, and medical devices between others [10]. The antimicrobial effectiveness of AgNPs is influenced by some physicochemical properties such as shape, size, structure and capping, which can vary depending of the method used for their synthesis [11–13]. Physicochemical methods are commonly used for AgNPs synthesis due to the highly monodispersion and size/shape homogeneity of the suspensions obtained, however, this methods implies high-energy input, the use of toxic solvents and stabilizing/reducent agents [14]. In order to find ecofriendly, low-energy consumer and inexpensive methods for AgNPs synthesis, the number of studies about biological synthesis (also called “biosynthesis”) using different biological organisms increased during the last two decades [10,15–17]. The interest in biosynthesis could be due to the advantage that this kind of synthesis (also called “green synthesis”) represents to the environmental care with respect to the injury caused by reagents used in conventional methods of synthesis, moreover, biosynthesis involve high yield and stability; however, the homogeneity of size and shape is frequently a challenge [16].

The antibacterial properties of AgNPs has been widely demonstrated in different species of both pathogenic and non-pathogenic bacteria [11, 18–20]. The mechanisms proposed to explain the cellular damage caused by AgNPs includes disruption of the membrane potential produced by the electrostatic interactions of AgNPs with the membrane, and the internalization of AgNPs with the subsequent liberation of Ag ions that promotes increase of oxidative stress and consequently a damage to DNA and respiratory chain proteins [21–24]. The antibiofilm activity of AgNPs has widely demonstrated in pathogenic bacteria [22–23, 25], however, the studies about the effect of AgNPs over the formation or destruction of UPEC biofilms are still limited [26]; this can be considered an important aspect on prevention of UPEC infection and dissemination. This work was focused in the establishment of a simple, efficient and low cost method for synthesis of AgNPs with antibiofilm activity against the UPEC. Biosynthesis of AgNPs using a native fungi isolated from soil was achieved and its ability to prevent the formation of biofilms by a pathogenic bacteria (UPEC) was demonstrated.

## Materials and methods

### Fungi and bacterial strains

The fungal strain Ag0.5–5 used in this work was isolated in presence of AgNO_3_ at 0.5 mM from mining tails located at Zacatecas, Mexico (22°47’01.4”N 102°36’21”W and 2426 m.a.s.l.). The uropathogenic *E*. *coli* (UPEC-PAM5) is a clinical isolate from the stock of the Infectious Diseases Laboratory (Zacatecas Autonomous University) with phenotypic and genotypic characteristics involved in biofilm formation such as the production of exopolysaccharide and the presence of three important genes, *csgA* (curli fimbriae), *fimH* (type I fimbriae) and *flu* (antigen 43), related to the formation of *E*. *coli* biofilms.

#### Identification and morphological characterization of the AgNPs producer fungi

The phenotypic characterization of the Ag0.5–5 strain was based on the description of colony and morphological characteristics in three different culture media: PDA (Difco); Sabouraud (Difco) and Czapek (Difco) was carried out. The cell morphology was observed in fungal microcultures by light microscopy using a cotton blue solution (1%) as a contrast dye. The genus of the Ag0.5–5 strain was established accordingly to morphological characteristics previously described [27]. The phenotypic identification was complemented by a molecular approach by sequencing of the internal transcribed spacers (ITSs) of the rDNA genes. Briefly, 100 ng of genomic DNA was used for amplification of the ITSs regions by PCR under previously reported conditions with primers ITS1 (5' TCCGTAGGTGAACCTGCGG 3' corresponding to the end of the 18S gen) and ITS4 (5' TCCTCCGCTTATTGATATGC 3' corresponding to the initial region of the 28S gene) [28]. The PCR products were purified with the Wizard SV Gel and PCR Clean-Up System Kit (Promega, Madison, USA), and sequenced using the primers used in PCR. The sequences were assembled and edited using the DNASTAR Lasergene v.7.1 software and compared to the ITSs rDNA of fungal sequences deposited in the GeneBank database of the National Center for Biotechnology Information (NCBI) server and in the CBS-KNAW Fungal Biodiversity Centre’s Fusarium MLST website (http://fusarium.mycobank.org). Sequence alignments were performed using the BLASTN program of the NCBI server, using the MEGABLAST algorithm for highly similar sequences and default parameters to determine the sequence identity percentages.

The BLASTn query in the CBSKNAW’s Fusarium MLST database was carried out using the reference files for *Fusarium* database and default parameters.

### Fungal Ag-tolerance and biosynthesis of the AgNPs

To test fungal tolerance to Ag ions (Ag^+1^), the minimum inhibitory concentration (MIC) was determined. Briefly, an agar plug of a fungal colony was inoculated onto PDA plates supplemented with concentrations of AgNO_3_ (0, 0.25, 0.5, 0.75, 1.0, 1.5, 2.0, 2.5 and 3.0 mM) and incubated at 28°C during 7 days. The lowest concentration of metal salt that prevented the growth was recorded as MIC. The fungal extracellular biosynthesis of AgNPs, was carried out as previously described [29]. Briefly, 50 mL of PDB media (Difco®) were inoculated with 7X10^6^ conidia and incubated (28°C, 180 rpm, 72 h). The mycelium was separated from media by vaccum filtration under sterile conditions; 2.5 g of mycelium was added to 50 mL of deionized sterile water and incubated at the same conditions. Mycelium was separated by vaccum filtration and the free-cell filtrate was supplemented with 1.0 mM of AgNO_3_ and incubated (28°C, 180 rpm, 168 h) in dark conditions. Simultaneously, media culture without fungi inoculant was treated under the above conditions and used as negative control of AgNPs synthesis, whereas a fungal culture without the addition of the metal salt was obtained at the same conditions to be used as blank in the AgNPs physicochemical analyses. The silver concentration associated to AgNPs was estimated by measuring the concentration of the non-reduced silver ions into AgNPs that remaining soluble in the supernatant of the free cell filtrate. The AgNPs suspension was centrifuged (14 000 x g, 1 h) to pellet the AgNPs and supernatant was analyzed by Energy dispersive X-Ray fluorescence in an EDXRF Spectrometer (Rigaku® NEX QC+ QuantEZ) to determine the concentration of non-reduced silver ions. The concentration of silver associated to AgNPs was calculated with the formula: C_AgNPs_ = CAg ions—C_AgSN_, where, C_AgNPs_ is the silver concentration associated to the AgNPs in the suspension, C_Ag ions_ is the total concentration of silver ions used for the biosynthesis, and C_AgSN_ is the concentration of non-reduced silver ions remained in supernatant after centrifugation of AgNPs.

### Characterization of AgNPs synthesized by fungi

In order to reveal the crystalline structure, size and the elemental chemical composition of AgNPs, the nanoparticles were analyzed by UV-Vis and Infrared (IR) Spectroscopy, X-ray Diffraction (XRD), Scanning Transmission Electron Microscopy (STEM), high resolution Transmission Electron Microscopy (HRTEM), and Energy-dispersive X-ray Spectroscopy (EDX). The TEM analysis was performed in a JEM-2200FS microscope that operated at 200kV of acceleration voltage and has attached a NORAN spectrometer. Bioreduction of silver ions was monitored spectroscopically in the aliquots obtained from fungal filtrates by measurement of light absorption at the UV-Vis range, using a spectrophotometer (Perkin Elmer, model Lambda 35) at a wavelength range between 200 and 900 nm. The data were processed in the Origin 8.0 software and the absorption spectra were obtained. For Infrared (IR) spectroscopy, samples of the fungal filtrates were analyzed in an IRTracer-100 (Shimadzu) Spectrophotometer and data were recorded for wavenumbers ranging from 400–4,000 cm^-1^, with a scan rate of 0.2 cm/sec and resolution of 4 cm^-1^. The data were processed in the Origin 8.0 software for obtaining of the IR spectra. For the XRD analysis, the fungal biosynthesized AgNPs suspension and the fungal water-filtrate (as a negative control of biosynthesis) were lyophilized and grinded to obtain a dry/fine powder of the samples that were analyzed in a diffractometer SmartLab (Rigaku) with a diffracted beam of CuK_α,_ λ = 1.54060 Å at 40 Kv and 44 mA. The diffraction patterns were obtained with an angular range from 10 to 80° and 2°/min. The code numbers of the crystalline phases detected in the samples were identified using the X’Pert HighScore Plus software (Rigaku PDXL 2.4.2.0 version). For TEM, samples were prepared by placing a drop of the AgNPs suspension on a carbon coated copper grid and dried at room temperature.

### Analysis of the antibacterial effect of fungal biosynthesized AgNPs over the planktonic cells of UPEC

The bactericidal effect of AgNPs was analyzed by the agar well-diffusion method and by determination of viability of bacterial cells exposed to AgNPs. First, a culture of UPEC-PAM5 at the middle of the log phase was prepared in nutrient broth. A volume of 0.1 mL of the culture (containing 7 X10^8^ UFC/mL) was spread out onto Muller–Hinton agar plates with wells of 6-mm diameter at the center of the plate. AgNPs suspensions at concentrations of 2.5, 7.5, 10, 15, 25 and 35 mg L^-1^, were deposited into the wells of the plates and incubated (37°C/28 h). The inhibition zones formed around the wells were measured. For viability assay, 1 mL of a bacterial suspension containing 7X10^8^ CFU/mL, was deposited by triplicate in 24-wells polystyrene plates (Nunclon Delta Surface, Nunc^TM^) and treated with AgNPs at final concentrations of 0, 7.5, 15, 25, and 35 mg L^-1^. The plate was incubated (37°C/120 rpm) and 0.1 mL was taken at different times (0.5, 1, 2 and 3 h) of incubation for serial dilution. Finally, 0.1 mL of each dilution was plated onto LB agar and incubated at 37°C/for 18 h, the colonies formed were counted and the bactericidal effect of AgNPs over planktonic UPEC was reported as colony forming units per milliliter (CFU/mL) as a measure of viability.

### Analysis of the antibacterial effect of fungal biosynthesized AgNPs over the biofilm formation and established biofilms of UPEC

The effect produced by AgNPs over UPEC biofilms was determined quantitatively by the crystal-violet stain method previously described [30] using bacterial suspensions in M63 minimal media containing 3X10^7^ CFU/mL for the biofilm formation. Different concentrations of AgNPs (0, 7.5, 15, 25, and 35 mg L^-1^) were added during the process biofilm formation and after that biofilm was established. The colorant crystal violet attached to cells in biofilms was solubilized with 1 ml of absolute ethanol and the optical density (OD) of each well was measured spectrophotometrically at 590 nm. Non-inoculated media were included as negative control in each experiment. Each condition evaluated was tested in triplicate and experiments were repeated three times.

For qualitative analysis, replica of plates 1 and 2 were prepared and treated with the AgNPs as describe above. Both plates were washed twice with PBS and stained with a mixture of 6 μM SYTO 9 and 30 μM potassium iodide from Live/Dead BacLight Viability kit L13152 (Invitrogen Molecular Probes, Inc. Eugene, OR, USA) for 15 min. The imaging of stained biofilms was performed using an Axiovert 25 CFL (Zeiss Göttingen, Germany) fluorescence microscope and documented with an AmScope Microscope Digital Camera (MU300 3.0 MP USB). The experiment was repeated 3 times.

### Statistical analysis

For determination of bactericidal effect of AgNPs over bacterial biofilms, differences between the O.D. _590 nm_ values obtained with AgNPs treatments at different concentrations were calculated by performing a two tails t-test analysis in the Excel program of the Microsoft software package by comparison of each O.D._590 nm_ value obtained for each concentration, with the O.D._590 nm_ values obtained for the untreated cells and each of the concentrations analyzed. The significance was set at a *P* < 0.05. The letters a, b, c and d was used to indicate statistical differences between the AgNPs concentrations tested.

## Results

### Identification and morphological characterization of the AgNPs producer fungi

**Fig 1:**
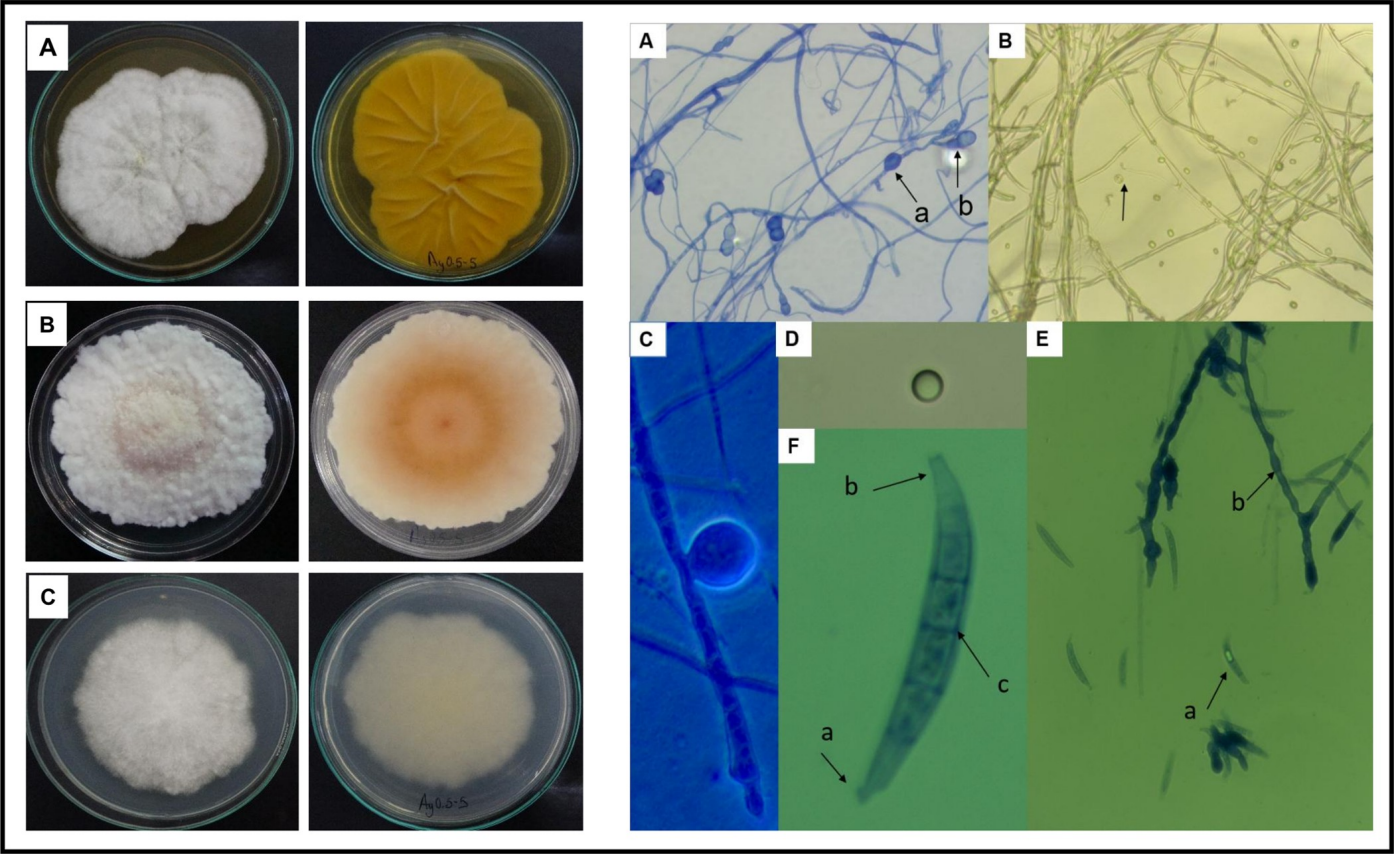
Colony and microscopic characteristics of the Ag0.5–5 isolate. *Left panel*: Colonies in different media cultures are shown: (A) Sabouraud agar, (B) PDA and (C) Czapek agar. *Right panel*: *In vitro* microcultures of the isolate were prepared and the cellular structure of fungi was observed microscopically: (A) Mycelium with intercalar (a) and terminal (b) chlamydospores in Czapek agar (Bright field, 40X with blue cotton as contrast dye). (B) Septated mycelium and microconidia in PDA agar (Bright field, 40X). (C) Intercalar chlamydospore in Czapek agar (Bright field, 100X with blue cotton as contrast dye). (D) Microconidia globosa in PDA (Bright field 100X). (E) Macroconidia (a) and chain grouped chlamydospores (b) in Czapek agar (Bright field, 40X with blue cotton as contrast dye). (F) Macroconidia in Czapek agar, septum (c), basal cell (a) and blunt apical cell (b) (bright field, 100X with blue cotton as contrast dye).

The morphological characterization was based on description of the fungal colonies and microscopic structures of the Ag0.5–5 strain in different media cultures. In Sabouraud agar, a white cotton colony with regular edge, radial growth and aerial mycelia was observed in the obverse of colony, whereas a yellow pigment was present in the reverse side of the colony (Fig 1A, left panel). In PDA media, a white cotton colony with irregular edge and abundant aerial mycelia, was formed in the obverse face, whereas a beige color was observed in the reverse face of the colony (Fig 1B, left panel). In Czapeck agar, a slow growing and white cotton colony with irregular edge and scarce mycelia was observed (Fig 1C, left panel). A microscopically analysis of fungi cells revealed that in PDA and Sabouraud agar, a septate hyaline basal mycelia and single rounded microconidia in the aerial mycelia is formed (Fig 1B, right panel). In Czapeck agar, multiseptated curved macroconidia (Fig 1E and 1F, right panel), terminal and intercalar chlamydospores (a kind of asexual resistance spores) in old cultures were observed (Fig 1A, 1C and 1E, right panel). Taking together, all the characteristics including the morphology of colonies and sexual reproduction structures microscopically observed in microcultures, correlates with those described for *Fusarium* species [27,31].

Accordingly to the morphological characteristics, the molecular approach based on analysis of the Ag0.5–5 ITSs rRNA sequence (GenBank, access number MN633390), revealed that the Ag0.5–5 strain belongs to the *Fusarium* genus (*Ascomycota* phylum, *Ascomycetes* class, *Hypocreales* order). This classification is supported by results of the sequence alignment obtained using the Genebank database of the NCBI, which showed a 100% of identity and coverage with sequences of *Fusarium equiseti*. However, it has been reported that about a 50% of ITSs and LSU (Large Subunit) rDNA *locus* sequences of *Fusarium* that are deposited in NCBI are misidentified [32]. Thus, in order to obtain an accurate identification, we carried out a BLASTn in the CBSKNAW’s *Fusarium* MLST (Multilocus Sequence Typing) database (http://www.cbs.knaw.nl/Fusarium/) [33]. The sequence alignment using the *Fusarium* database revealed a 100% of identity and coverage with several MLST-types (1a, 1c, 2b, 3a, 3b, 4a, 4b, 5d, 5e, 5f, 7a, 9a and 9c) of the *Fusarium incarnatum*-*equiseti* Species Complex (FIESC) (S1 File), all of which belong to the *Equiseti* clade of this species complex [34]. Importantly, two of these MLST-types (9a and 9b), are properly identified and recognized as *Fusarium scirpi*, a specie frequently confused with *F*. *equiseti* [27]. Moreover, no alignment with the MLST-types 14a, 14b or 14c (properly identified and recognized as *F*. *equiseti*) was observed. Based on this results and in previous reports which indicate that identification of *Fusarium* species using ITSs and LSU rDNA sequences is limited [32], we carried out a comparison of morphological key characters usually used to differentiate between *Fusarium equiseti* (Corda) Saccardo and *Fusarium scirpi* (Lambotte & Fautrey**)** [27], in order to determinate the specie of the Ag0.5–5 strain. The comparison of key characters (Table 1), revealed that in contrast with *F*. *equiseti*, the Ag-05-5 strain and *F*. *scirpi* produce microconidia; which is the principal key character used to differentiate *F*. *equiseti* and *F*. *scirpi* [27]. This result support the molecular identification of the Ag0.5–5 strain as *Fusarium scirpi*.

**Table 1:** Morphological key characters of *Fusarium equiseti* (Corda) Saccardo, *Fusarium scirpi* (Lambotte & Fautrey) and *Fusarium* sp. (Ag0.5–5).

Key character	*F*. *equiseti* (Corda) Saccardo^a^	*F*. *scirpi* (Lambotte & Fautrey)^a^	*Ag 0.5–5 (Fusarium* sp.)^b^
**Microconidia**	Absent	Ellipsoidal on polyphialides with three openings	Round/ellipsoidal
**General description of macroconidia**	Long to very long and slender. Pronounced dorsi-ventral curvature	Relatively slender, but widest at the center. Strong, pronounced dorsi-ventral curvature	Curved dorsal surface and a straighter ventral surface
**Macroconidia apical cell**	Tapering rounded or filamentous whip-like	Tapering and elongate	Elonged and tapering with blunt end. Sometimes filamentous, whip or needle-like
**Macroconidia basal cell**	Prominent foot-like shape	Well-developed foot- like shape	Prominent foot-like shape
**Number of macroconidia septa**	5 to 7	6 to 7 closer together in the middle	Usually 5
**Macroconidia conidiophore description**	Monophialides on branched conidiophores in sporodochia	Non described	Non determined
**Chlamydospore**	Singly, in chains or in clumps	Chains and clumps	Singly and in chains. Globose and hyaline
**Sporodoquia**	orange	Scarce and orange	Non determined
**Pigment on PDA**	Initially white and brown with age	Initially white and brown with age	Initially white and light brown with age

^a^ Key characters described in Leslie and Sumerell, 2006 [27].

^b^ Key characters described in this study.

### Fungal Ag-tolerance and biosynthesis of the AgNPs

**Fig 2:**
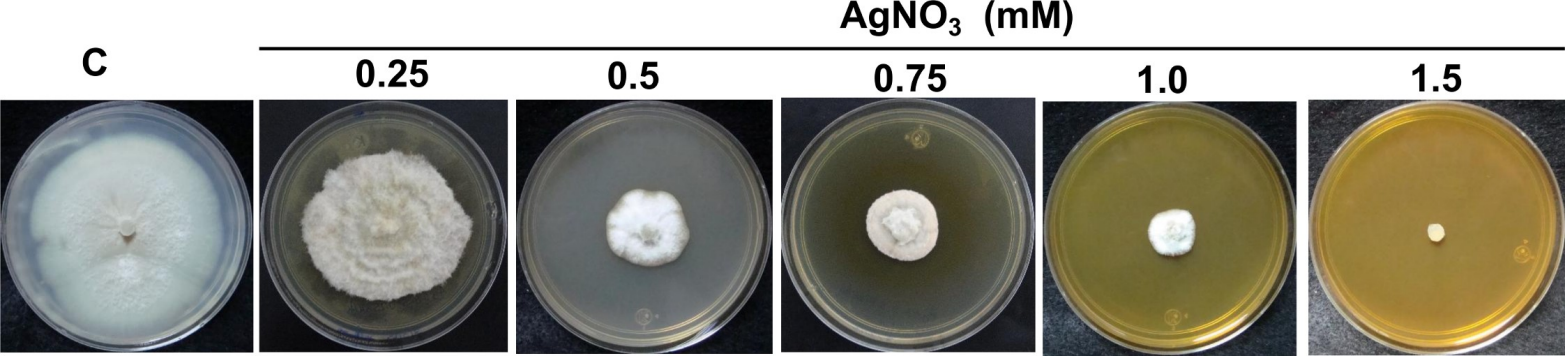
Silver tolerance of the Ag0.5–5 *Fusarium scirpi* strain. MIC (Minimal Inhibitory Concentration) of AgNO_3_ was determined in fungal cultures of PDA agar supplemented with increasing concentrations of AgNO_3. _

**Fig 3:**
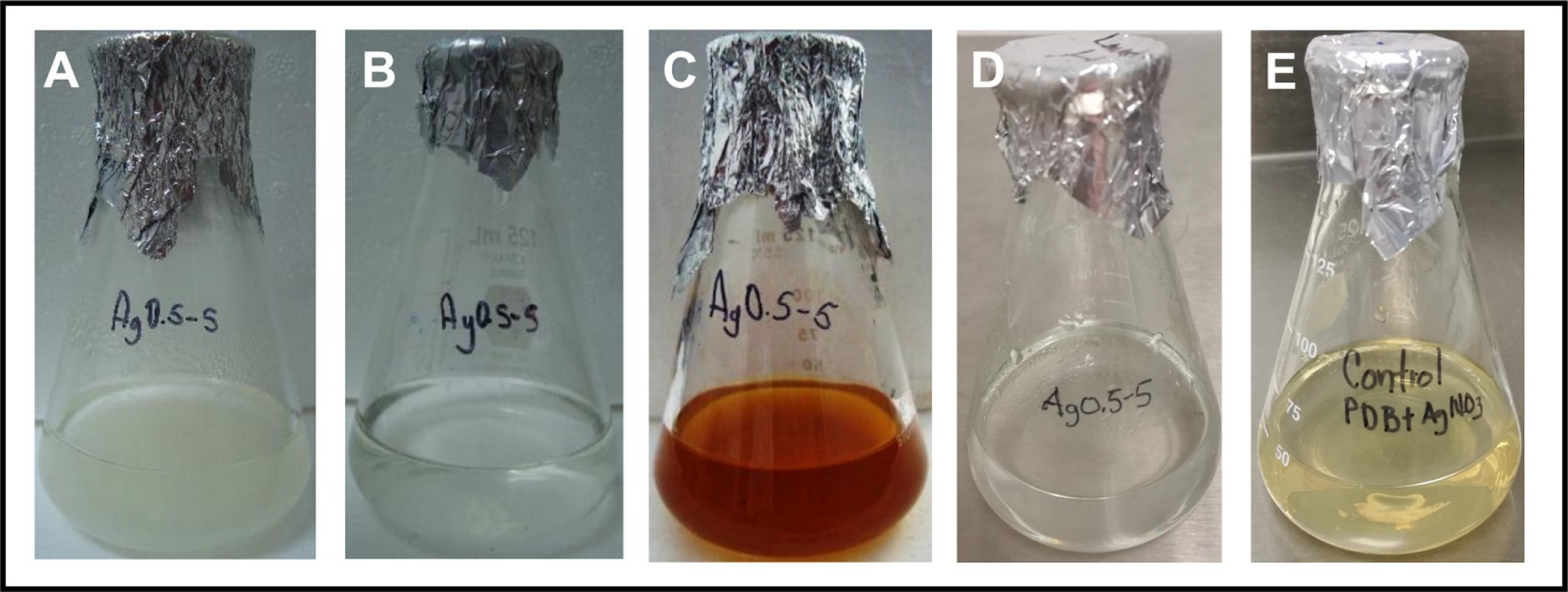
Extracellular synthesis of silver nanoparticles (AgNPs) by the Ag0.5–5 *Fusarium scirpi* strain. AgNPs were synthesized in water. (A) Fungal biomass incubated in sterile deionized water. (B) Free-cell water-filtrate. (C) Water-filtrate incubated with 1.0 mM of AgNO_3_ during 7 days. (D) Water-filtrate incubated without AgNO_3_ (control). (E) PDB culture media with 1.0 mM of AgNO_3_ and incubated.

**Fig 4:**
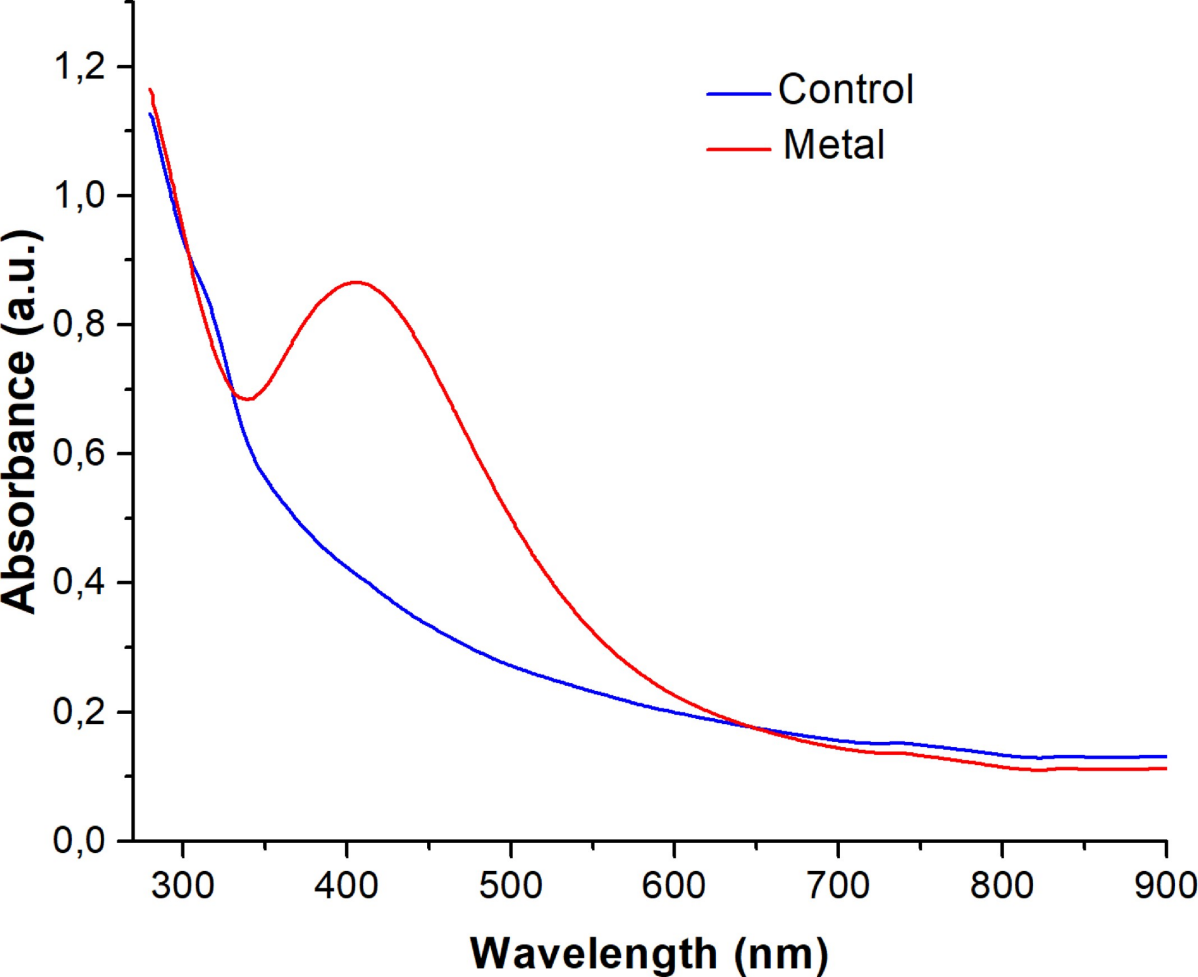
UV-Vis spectroscopy of AgNPs synthesized extracellularly by the Ag0.5–5 *Fusarium scirpi* strain. Absorption spectra of AgNPs formed in the free-cell H_2_O-filtrate of *Fusarium scirpi* (*red line*). The absorbance of the fungal filtrate without the addition of AgNO_3_ was analyzed as negative control of AgNPs synthesis (*blue line*).

The tolerance of the fungal strain Ag0.5–5 to the Ag ions was established in 1.5 mM of AgNO_3_ (Fig 2). The biosynthesis of AgNPs using the free cell water-filtrate (Fig 3B) obtained from a culture of the fungal biomass in water (Fig 3A), was successfully achieved by the addition of 1 mM of AgNO_3,_ and verified by a color change from a colorless (Fig 3B) to brown after 7 days of incubation with the metal solution (Fig 3C). The change in color observed, correlates with a maximum absorption peak in the UV-Visible spectra at 405 nm (Fig 4, *red line*), a characteristic property of AgNPs suspensions that is produced by the surface plasmon resonance (SPR) of the AgNPs. The color change and the absorption peak at 405 nm was not observed in the fungal water-filtrate without addition of metal used as control (Figs 3D and 4, *blue line*), neither in the negative control of metallic salt added to the PDB culture media (Fig 3E). The concentration of silver associated to AgNPs in the suspension obtained, was calculated about 76 mg L^-1^ using the formula C_AgNPs_ = CAg ions—C_AgSN_ (C = 107.86 25 mg L^-1^–32.1 mg L^-1^) as described in Materials and methods. Where, C_Ag ions_ is the total concentration of silver ions used for the biosynthesis, in this case 107.86 mg L^-1^ corresponding to 1 mM, and C_AgSN_ is the concentration of non-reduced silver ions determined by EDXRF that remain in the supernatant after centrifugation of AgNPs. This concentration indicates that an 81.7% of the total silver nitrate added to media was reduced to AgNPs by the fungal strain.

### Characterization of biosynthesized AgNPs

**Fig 5:**
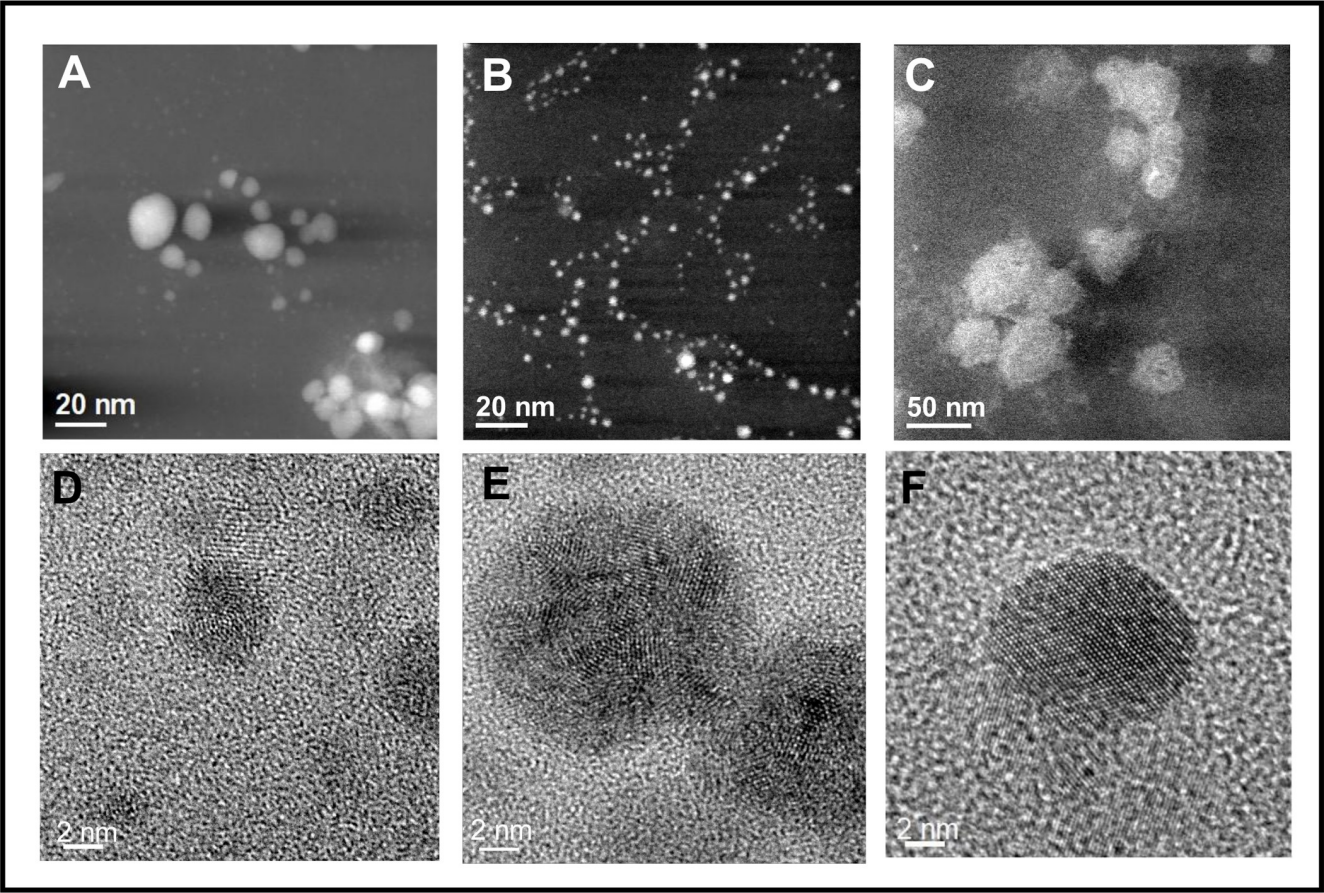
Annular dark field ADF-STEM images **(A-C)** and HRTEM images **(D-F)**. The biogenic AgNPs suspension formed by the Ag0.5–5 strain of *Fusarium scirpi* (A and B) and the fungal water-filtrate without addition of metal used as control (C) were deposited over copper grids. Brighter dots with different sizes of metallic AgNPs (A—B) are displayed in the ADF-STEM images and atomic resolution images of single nanoparticles are showed in *lower panels* (D—E).

**Fig 6:**
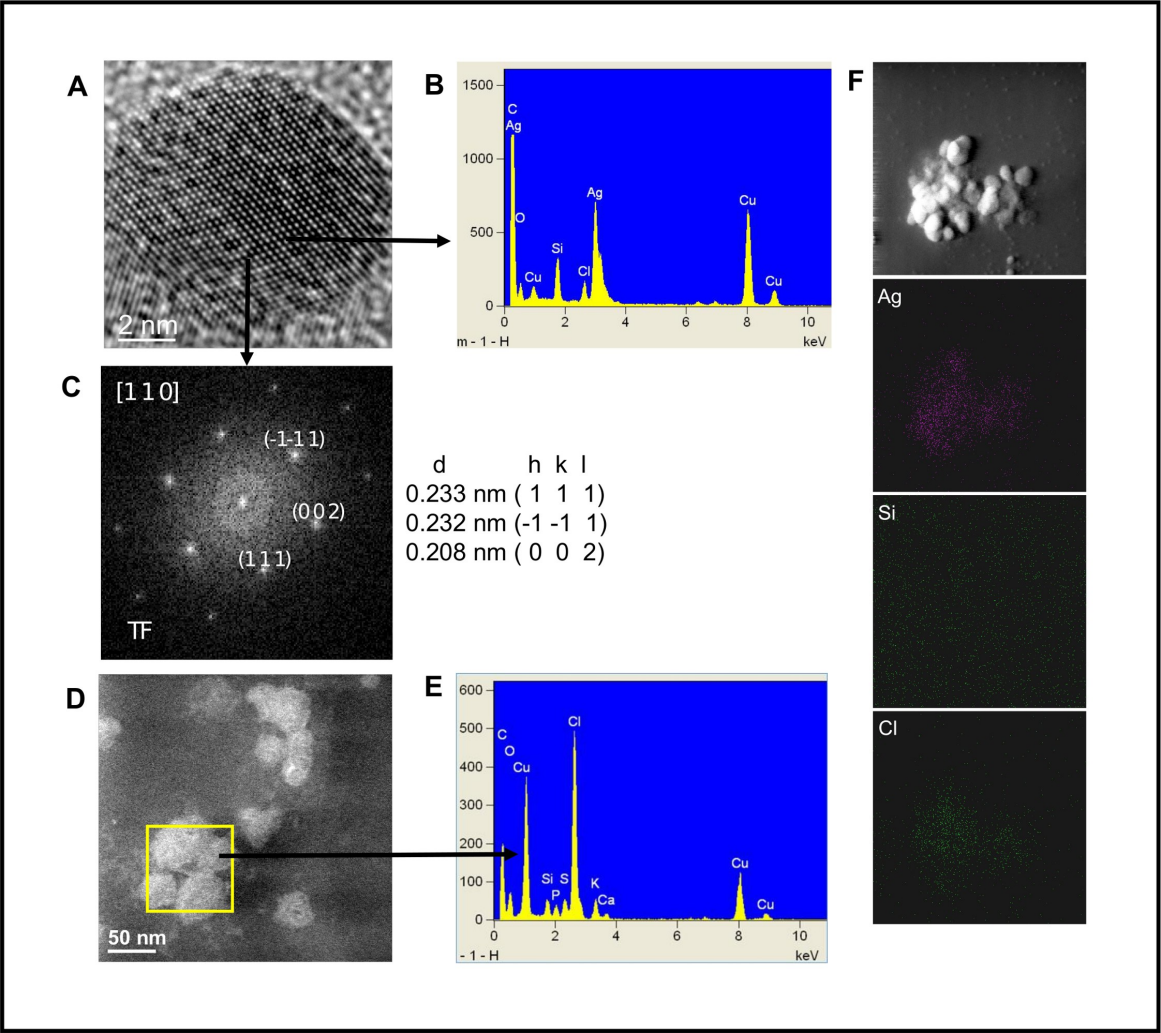
Crystalline structure and elemental composition of AgNPs biosynthesized with free-cell water-filtrate of the *Fusarium scirpi* Ag0.5–5 strain. (A) HRTEM image that shows an AgNP. (B) Energy dispersive X-ray (EDX) spectrum obtained in the AgNP. (C) FFT pattern of the atomic resolution image. (D) ADF-STEM image that shows the amorphous material in the negative control sample and the area selected for EDX analysis. (E) EDX spectrum of the negative control selected area. (F) Elemental chemical mapping of an AgNP cluster.

AgNPs suspension was analyzed by STEM and HRTEM (Fig 5), and results corroborates the presence of quasi-spherical AgNPs in a size range from 2 to 20 nm (Fig 5A, 5B, 5D–5F), with predominance of AgNPs with size around 2 nm and a minor AgNPs population with size around 20 nm. The AgNPs were absent in the fungal water-filtrate without metal used as control, on which amorphous material was observed (Fig 5C). The elemental EDX and the Fast Fourier Transform (FFT) analyses (Fig 6B and 6C, respectively) of a nanoparticle observed by HRTEM (Fig 6A), revealed the crystalline and metallic Ag^0^ composition of the AgNPs. As shown in Fig 6C, the FTT patterns indicate that the Miller indices and d-values (0.233 nm [111], 0.232 nm [–1–11], 0.208 nm [2]) of the crystallographic planes corresponds to a fcc (face centered cube) lattice of metallic silver (Ag^0^). Moreover, the EDX analysis detected strong signals of silver (Ag) (Fig 6B) that were absent in the amorphous material observed in the control (Fig 6D and 6E). Additionally, moderate signals of elements such as oxygen (O), silicon (Si), chlorine (Cl), potassium (K), calcium (Ca), carbon (C) and copper (Cu), were detected in both AgNPs and control suspensions analyzed, the two latest due to the carbon coated copper grid used for this analysis (Fig 6B, 6E and 6F).

**Fig 7:**
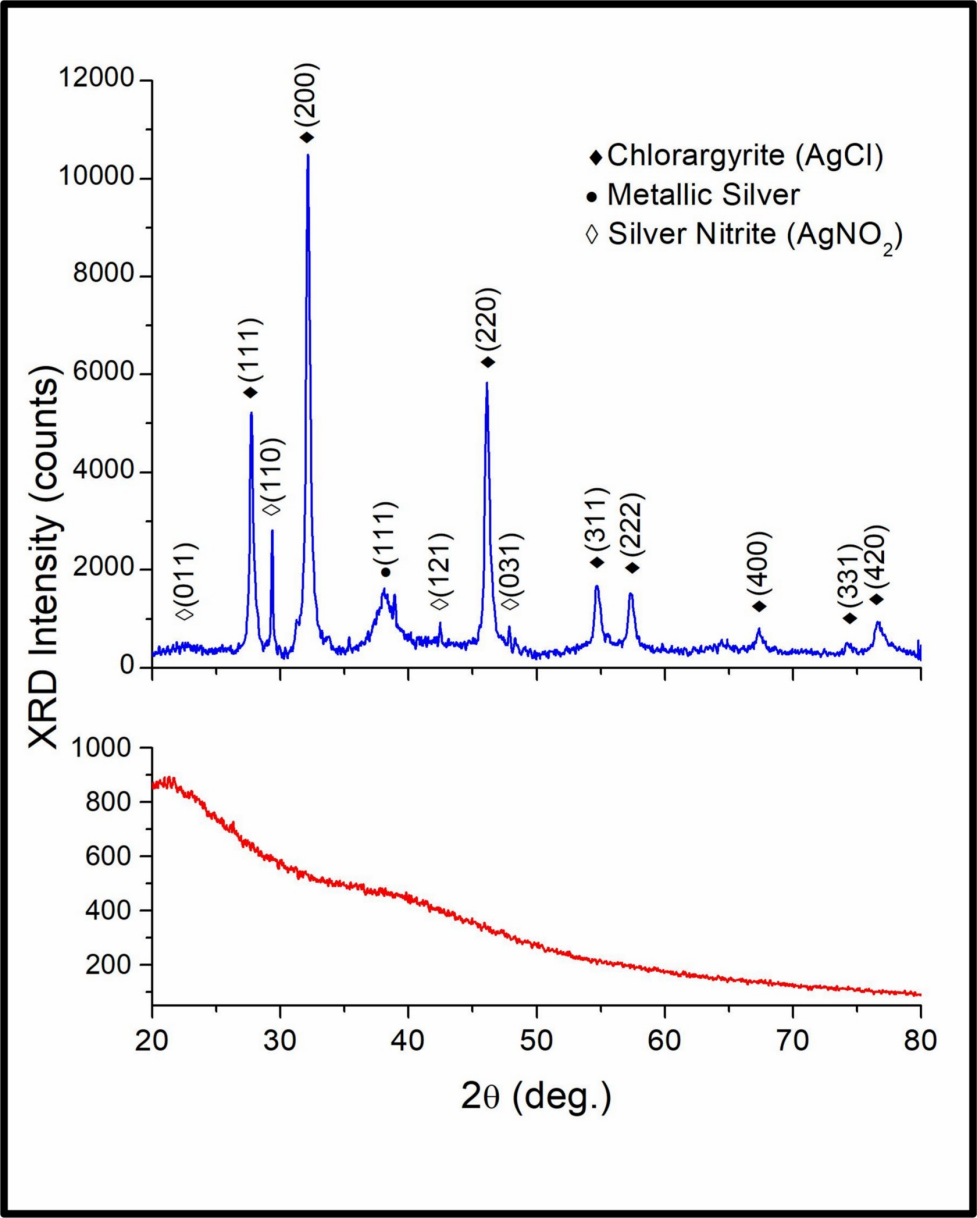
X-ray diffraction pattern of the fungal biosynthesized AgNPs powder. Suspension of the biosynthesized AgNPs was lyophilized and grinded to prepare the samples for XRD analysis. The X-ray diffraction pattern obtained from the AgNPs powder (*blue line*) and the fungal water-filtrate used as a negative control (*red line*) are shown.

In the XRD pattern obtained from samples of the AgNPs powder (Fig 7 *blue line*), several peaks were observed at 2θ = 27.76°, 32.11°, 46.15°, 54.72°, 57.39°, 67.32°, 74.32° and 76.93°, which correspond to (111), (200), (220), (311), (222), (400), (331) and (420) Bragg’s reflections of the face-centered cubic (fcc) phase of chlorargirite, respectively (AgCl, COD PDF # 96-901-1667) [35]. The peak observed at 2θ = 38.93° corresponds to (111) Bragg’s reflection of a face-centered cubic (fcc) phase of metallic silver (Ag^0^, COD PDF # 96-110-0137) [36]. Finally, the peaks at 2θ = 22.39°, 28.93°, 29.39°, 42.65°, 45.81° and 47.52°, correspond to (011), (020), (110), (121), (112) y (031) Braggs’s reflections of the orthorhombic structure of silver nitrite (AgNO_2_, JCPDS PDF # 06–0349). In the XRD pattern of the control sample (fungal water-filtrate) no evident diffraction peaks were observed (Fig 7 *red line*). Using the highest intensity diffraction peaks centered at 2θ = 32.11° and 38.93°, and the full-width at half-maximum (FWHM) β with values of 0.34946° and 1.8591° for chlorargirite and metallic silver patterns respectively, the average of the crystallite diameter *D* was obtained by the Scherrer formula *D = (0*.*9)λ / (β cosθ*_*β*_*)*, where λ is the wavelength (1.5406 Å), θ_β_ is the Bragg angle. The average D values obtained were approximately of 24 nm for the AgCl nanocrystals and 5.6 nm for the AgNPs. These D values are in agreement with the AgNPs dimension observed by SEM (Fig 5A and 5B) and HRTEM (Fig 5D–5F).

**Fig 8:**
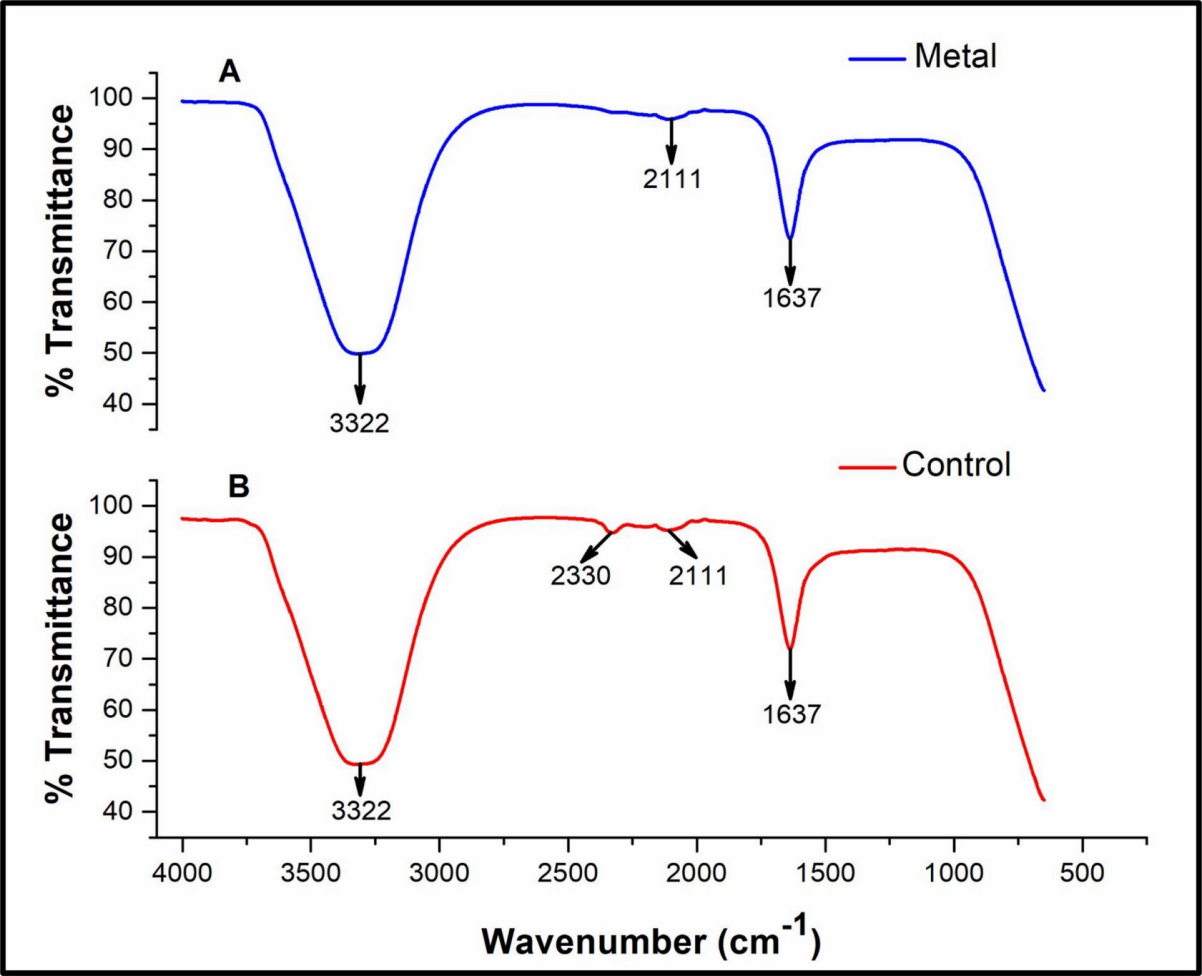
IR Spectroscopy of AgNPs synthesized extracellularly by the Ag0.5–5 *Fusarium scirpi* strain. IR spectra of AgNPs formed in the water-filtrate of *Fusarium scirpi* cultures (*blue lines*). The IR absorption of the fungal filtrate without the addition of AgNO_3_ was analyzed as controls (*red lines*).

In order to investigate whether fungal-derived molecules are present during the biosynthesis process of AgNPs, an analysis by FTIR spectroscopy of both the AgNPs suspension and the fungal water-filtrate without AgNO_3_ used as control, indicate the presence of bands at wavenumbers corresponding to three functional groups (Fig 8). A broad band with wavenumbers around the 3326- and 3322- cm^-1^ was assigned to the stretching vibration of the O-H group. A subtle band with wavenumber at 2111 cm^-1^ corresponding to the stretching vibration of a C-H bond, and a stretching band with wavenumbers around 1637- and 1640- cm^-1^ assigned to the asymmetric stretching vibration of the C = O group [37].

### Antibacterial effect of fungal biosynthesized AgNPs over the planktonic cells of UPEC

**Fig 9:**
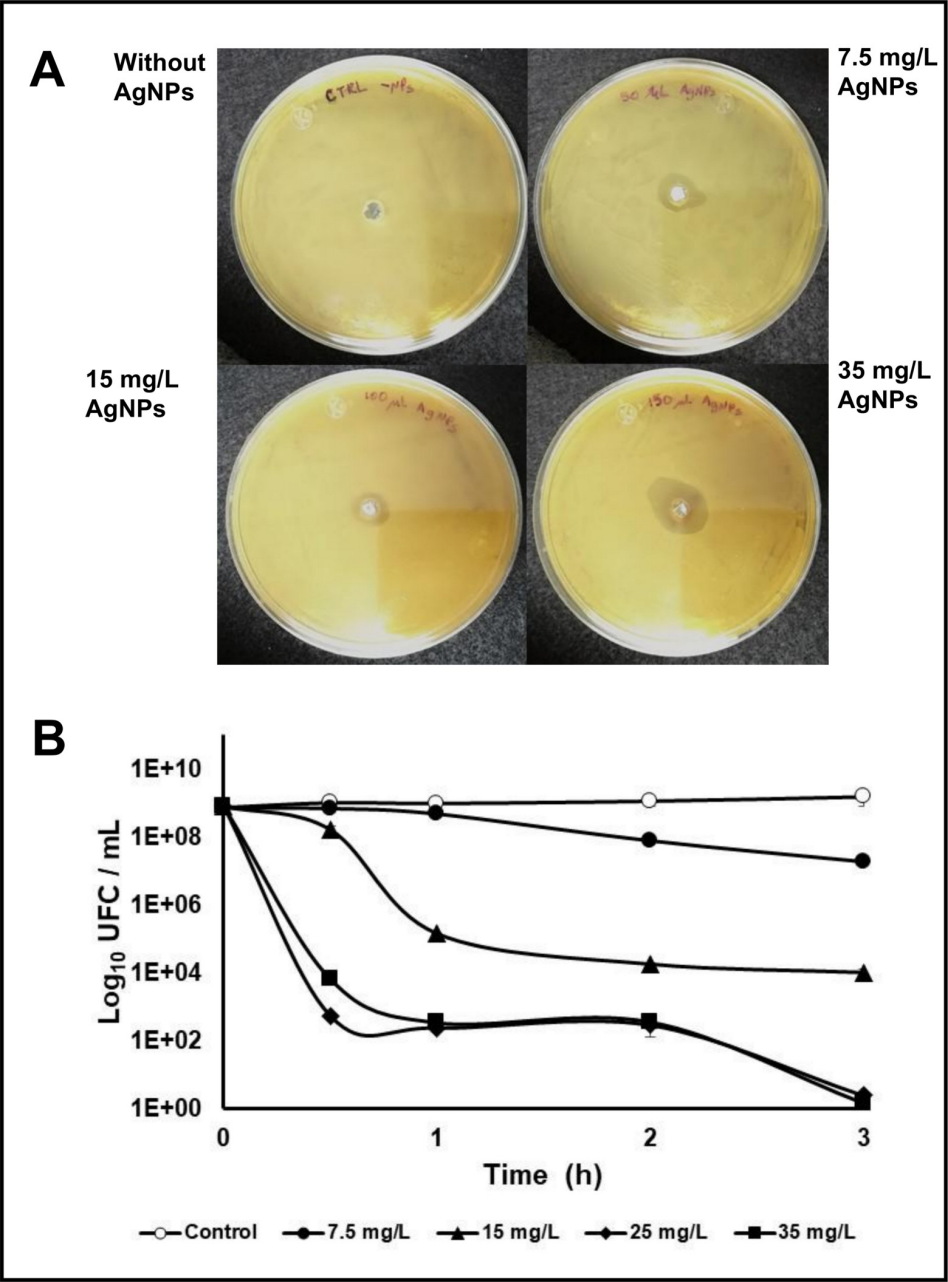
Antibacterial effect of fungal biosynthesized AgNPs over the planktonic cells of uropathogenic *E*. *coli* (UPEC). (A) Diffusion agar plates that show the growth-inhibition zones produced by the exposition of UPEC-PAM5 cells to increasing concentrations of AgNPs. (B) Cell viability in UPEC-PAM5 bacterial suspensions at different times of treatment with increasing concentrations of fungal-biosynthesized AgNPs. Viability without addition of AgNPs was used as control test.

The potential of biogenic AgNPs to inhibit the growth of pathogenic and well biofilm former bacteria such as UPEC, was evident in a well diffusion assay showed in Fig 9A, on which growth-inhibition zones in all concentration tested were produced, in a dose-dependent manner when AgNPs were present. Moreover, the viability of UPEC planktonic cells treated with AgNPs decreased as the concentration and time increases (Fig 9B). These results indicate that although all the concentrations tested produced a viability decrease, a minimal inhibitory concentration of 25 mg/L, was sufficient to reduce the viability about six orders of magnitude after 1 h of exposition and abolished bacterial viability after 3 h of treatment with AgNPs (Fig 9B).

### Antibacterial effect of fungal biosynthesized AgNPs over the biofilm formation and established biofilms of UPEC

**Fig 10:**
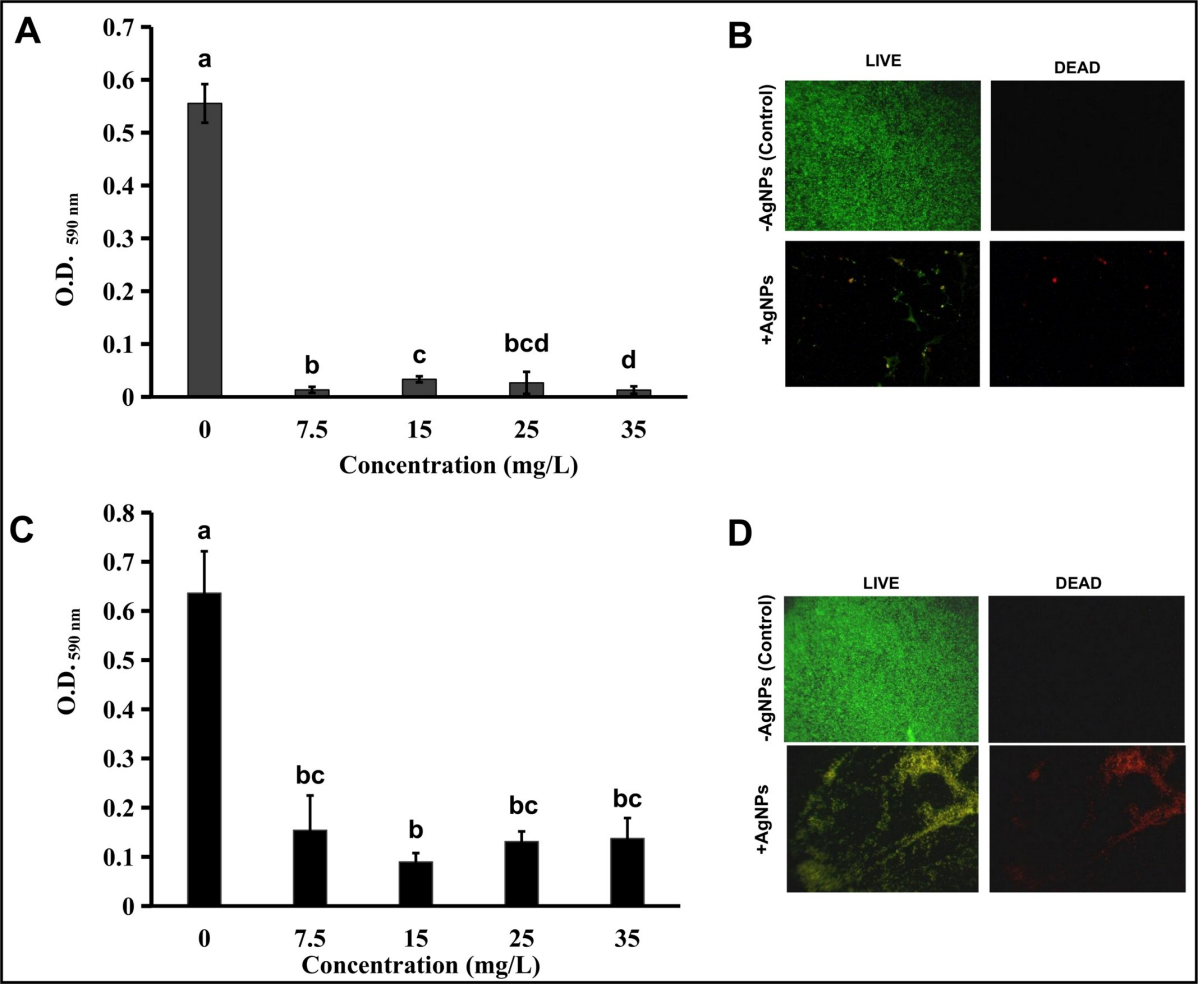
Effect of fungal biosynthesized AgNPs over biofilm formation and preformed biofilms of uropathogenic *Escherichia coli* (UPEC). (A) Inhibition of biofilm formation of cell suspensions treated with different AgNPs concentrations and determined by measurement of crystal violet dye attached to biofilm-associated cells at OD _590 nm_. (B) Damage produced over established biofilms treated with different AgNPs concentration and determined by measurement of crystal violet dye attached to biofilm-associated cells at OD_590 nm_. (C and D) Micrographs of UPEC-PAM5 strain treated with 7.5 mg/L AgNPs (+AgNPs) or without AgNPs (-AgNPs) before the biofilm formation (C) and in preformed biofilms (D). Bacterial cells were stained with Live/Dead BacLight Viability kit and observed by fluorescence microscopy at 40X amplification. Each bar in graphs (A and C) represents the mean of data collected from three independent experiments done in triplicate, and the error bars represent standard errors of the means (SEM). The statistical differences (a, b, c, and d) between the biofilm formation at the different AgNPs concentration tested, as determined by t-test (*P<*0.05), are shown above each bar.

The ability of the biogenic AgNPs to inhibit the formation and/or disrupt established UPEC biofilms was tested in a colorimetric assay that measure the associated-dye to cells in biofilms. The results indicated that formation of UPEC biofilms was dramatically inhibited (around a 97%) in all the AgNPs concentrations used respecting to untreated bacterial cells; this result was supported by the statistical differences encountered between the untreated and the treated bacterial cells with AgNPs (Fig 10A). This inhibition was also evident in microscopic observations of bacterial cells that were treated with 7.5 mg/L of AgNPs. A scarce number of attached cells in the AgNPs treated samples were red stained as indicative of cellular dead, whereas the untreated cells used as control were green stained, as indicative of live cells (Fig 10B). Additionally to the strong inhibition of biofilm formation observed, the effect that AgNPs produce over established UPEC-biofilms was also evaluated and results revealed that after the treatment with AgNPs, only a 20% of the bacterial cells in biofilms remains attached to the surface, this effect was observed in all AgNPs concentrations analyzed (Fig 10C). The microscopic analysis revealed an important damage of the biofilm structure when treated with 7.5 mg/L, this damage was observed as large patches of bacterial cells stained in red as indicative of cellular dead (Fig 10D).

## Discussion

The antibiofilm activity of the AgNPs over different pathogenic bacteria such as *Salmonella*, *Pseudomonas*, *Staphylococcus and E*. *coli* biofilms is well documented [23,25,26]. Thus, the AgNPs can be considered as an alternative to be used on surfaces of materials such as dentures, contact lenses, catheters and probes, in order to prevent biofilm associated infections [22,38]. The applicability of AgNPs could be limited by the high cost of pure AgNPs suspensions, however biological methods are extensively explored as an ecofriendly alternative for the AgNPs production at lower cost with respect to other methods [20]. Particularly, fungal biosynthesis has attracted the attention as a good “green” alternative for metal NPs production, due to the feasibility to use extracellular fungal molecules secreted as intermediaries of metal NPs synthesis [29,39]. In this study, a simple and “green” method for AgNPs biosynthesis was carried out using a soil fungal isolate that was tolerant to 1.5 mM of AgNO_3_. Based on morphological characters and the molecular analysis based on ITSs rDNA sequence, the fungal isolate Ag-0.5–5 used for AgNPs synthesis was identified as *F*. *scirpi*. However, it has been reported that the use of ITSs-LSU rDNA *locus* for *Fusarium* species identification is limited and frequently uninformative at the species-level [31], for this reason we consider that a robust morphology characterization and a MLST analysis could be necessary to support the classification of the Ag0.5–5 strain as *F*. *scirpi*. Although some of *Fusarium* species have been used for the AgNPs biosynthesis [40], to our knowledge, this is the first report on which *F*. *scirpi* is used for AgNPs synthesis.

The IR spectroscopy analysis suggested an interaction of the Ag ions with the fungi extracellular molecules secreted into water; which could be involved in reduction of Ag ions, nucleation and/or stabilization of AgNPs, as previously has been reported in other fungal species [40,41]. Physicochemical analyses indicate that the AgNPs obtained were crystalline, quasi-spherical and with a range size between 2 to 20 nm, these characteristics are consistent with those reported using a variety of fungus [40]. The concentration of silver associated to the AgNPs in suspension, was determined in 76 mg/L, this value corresponds to the 82% of the total silver used in the biosynthesis process (107.86 mg/L). Accordingly to this results, Lok and coworkers (2007), calculate that AgNPs suspensions contains approximately a 12% of silver ions; indicating that the yield of the synthesis method used in this study was similar to chemical methods [42]. However, the efficiency of the AgNPs biosynthesis process cannot be directly compared with other biosynthetic methods, due to differences between the methods used for quantification [25,43].

It has been shown that in contrast to the intracellular synthesis, that involves additional steps for AgNPs purification after biosynthesis [43–45]; the extracellular synthesis is cheap, favors large-scale production and requires simple downstream process for purification [18]. The method used in this work for the AgNPs biosynthesis in water, did not required additional steps such as ultrasound, filtration, or reaction with detergents to eliminate undesirable components; only centrifugation was used for elimination of the silver ions that remains in the AgNPs suspension, in order to discard the antibacterial activity that could be associated to silver ions. Thus, this method could be considered as suitable for large-scale production of AgNPs.

The activity of the AgNPs against bacterial biofilms has been widely reported [23]. Particularly, the antibiofilm activity of the AgNPs obtained by plant or microbial assisted biosynthesis, has been reported for several bacterial species such as *Pseudomonas putida* [25]; *P*. *aeruginosa* [26]; *E*. *coli* [25] and *S*. *epidermidis* [43]. In a recent work, Singh and coworkers (2018) demonstrated that biofilm formation of the UPEC strain UTI 89, was inhibited about an 80% in the presence of 200 mg/L of plant-biogenic AgNPs with a size range of 130 nm [26]. Accordingly to this report, the fungal AgNPs evaluated in this work, produced an inhibitory effect over the *in vitro* formation of UPEC biofilms as well as in the disruption of established biofilms. An inhibition of 97% of biofilm formation was achieved at 7.5 mg/L, at this concentration, only a slight decrease of viability was produced when planktonic cells were exposed to AgNPs; thus, a sub-MIC concentration of AgNPs was sufficient to avoid the establishment of the biofilm. This result suggest that lethal concentrations of AgNPs are not required to inhibit formation of UPEC-biofilms, probably because the antibiofilm activity of AgNPs is the result of the inhibition of gene expression during early-steps of the biofilm development, such as occurred with several antimicrobial peptides [46]. Thus, the efficiency of the fungal AgNPs to inhibit formation of UPEC-biofilms, was higher compared with those reported for plant derived AgNPs [26], and the AgNPs synthesized with starch that produced a maximum inhibition of 65 and 88% of biofilm formation in *P*. *aeruginosa* y *S*. *aureus*, respectively [47]. The differences between the antibiofilm activity of AgNPs against different bacterial species are probably due to the nature and size/shape of the AgNPs and to the structure/composition of the biofilm. On the other hand, the disruption of an 80% of mature UPEC-biofilms in presence of 7.5 mg/L of AgNPs and the presence of dead cells in the disrupted biofilm, suggest a high sensibility of UPEC to the AgNPs in mature biofilms. In contrast to this result, biofilms of *Pseudomonas putida* in late-steps of maturity were resistant to AgNPs and susceptible at early-steps of the biofilm development [25], suggesting again that the differences of biofilm structure between different bacterial species, and the physicochemical properties of AgNPs are important factors that affect its antibiofilm effectiveness.

**Fig 11:**
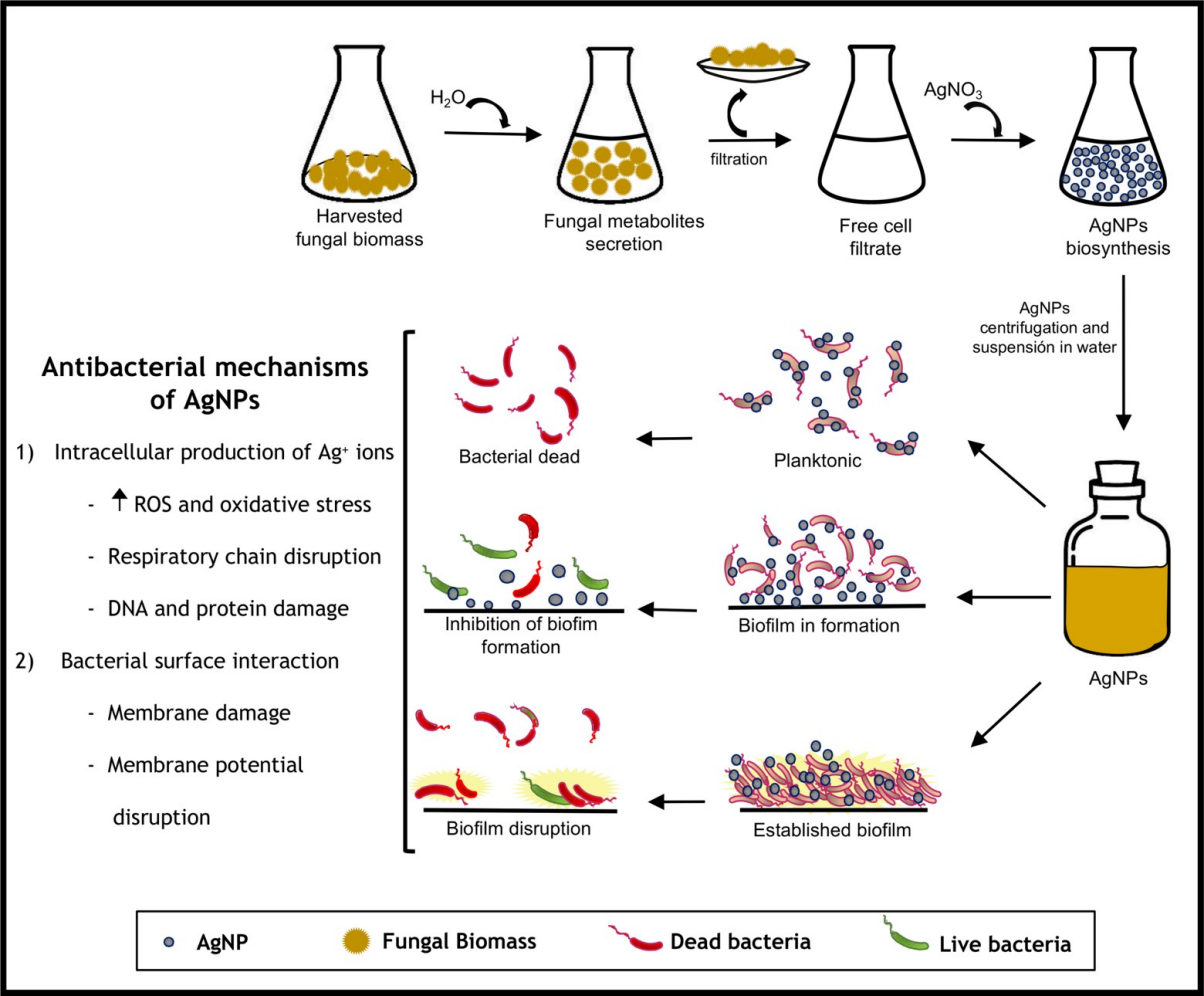
Schematic methodology of AgNPs synthesis, antimicrobial activity against UPEC biofilms and possible mechanisms involved.

The diagram depicted in Fig 11 shows the simplicity of the method used for fungal biosynthesis using the Ag0.5–5 strain of *F*. *scirpi*. The solely incubation of the fungal free-cell filtrate with the silver salt precursor produce AgNPs with a size between 2–20 nm. The biogenic AgNPs showed a differential and concentration-dependent antibacterial activity when planktonic, preformed or mature biofilms of UPEC was proved. This could suggest that bacterial aggregation and physiology are crucial factors that determinate the predominance of one or several of the mechanisms proposed for the AgNPs antibacterial activity. Some of these mechanisms include the increased oxidative stress induced by intracellular production of Ag^+^ ions, disruption of the membrane potential and the respiratory chain function, and the interaction with DNA and regulatory proteins [22–24].

The results of this study could be pivotal into the design of strategies to avoid the dissemination of antibiotic-resistant UPEC strains and consequently to decrease of the UTIs incidence. This strategies could be based on biosynthesis using *F*. *scirpi* Ag0.5–5, which implies some advantages over other biosynthetic methods such as low salt concentration required for biosynthesis, high antibiofilm activity (sub-MIC concentrations produce the antibiofilm effect) and simple down-process of centrifugation is required for AgNPs purification.

## Supporting information

S1 File Alignment of the ITS rDNA sequence of the Ag0.5–5 strain in *Fusarium* database. A BLASTn of the ITS rDNA sequence of the Ag0.5–5 strain (GenBank, access number MN633390), was carried out in the CBSKNAW’s *Fusarium* MLST (Multilocus Sequence Typing) database (http://www.cbs.knaw.nl/Fusarium/).
(PDF)
