# Supplementary material for: Biosynthesis of silver nanoparticles by Fusarium scirpi and its potential as antimicrobial agent against uropathogenic Escherichia coli biofilms
Source: PLoS One. 2020 Mar 12;15(3):e0230275. doi: 10.1371/journal.pone.0230275 (PMC7067426; doi:10.1371/journal.pone.0230275)
Supplement: S1 File — A BLASTn of the ITS rDNA sequence of the Ag0.5–5 strain (GenBank, access number MN633390), was carried out in the CBSKNAW’s Fusarium MLST (Multilocus Sequence Typing) database (http://www.cbs.knaw.nl/Fusarium/). (PDF) [file pone.0230275.s001.pdf]

#	Reference description	Score	Probability	Similarity	Fragments	Overlap%	Direction	Rating
1	Fusarium incarnatum-equiseti species complex (NRRL 34034; MLST type: 1-c) GQ505725 Fusarium incarnatum-equiseti species complex (NRRL 34034; MLST type: 1-c), Fusarium incarnatum-equiseti species complex (NRRL 34034; MLST type: 1-c), 1-c, USA AZ, Human leg, n7: 28S ribosomal RNA large subunit (28S - LSU)	721.158	0	100	2	96.761	+/+	****

Alignment

Reference sequence:
Fusarium incarnatum-equiseti species complex (NRRL 34034; MLST type: 1-c)
GQ505725
Fusarium incarnatum-equiseti species complex (NRRL 34034; MLST type: 1-c), Fusarium incarnatum-equiseti species complex (NRRL 34034; MLST type: 1-c), 1-c, USA AZ, Human leg, n7: 28S ribosomal RNA large subunit (28S - LSU)

Sequence length: 1132

Similarity: 454/454 [100.000 %], Gaps: 0 [0.000 %], Coverage: 454/454 [100.000 %]
Score: 721.158, Probability: 0, Direction: +/+

```
Qry 41       ACATACCTATACGTTGCCTCGGCGGATCAGCCCGCGCCCCGTAAAAAGGGACGGCCCGCC  100
             ||||||||||||||||||||||||||||||||||||||||||||||||||||||||||||
Ref 73       ACATACCTATACGTTGCCTCGGCGGATCAGCCCGCGCCCCGTAAAAAGGGACGGCCCGCC  132

Qry 101      CGAGGACCCCTAAACTCTGTTTTTAGTGGAACTTCTGAGTAAAACAAACAAATAAATCAA  160
             ||||||||||||||||||||||||||||||||||||||||||||||||||||||||||||
Ref 133      CGAGGACCCCTAAACTCTGTTTTTAGTGGAACTTCTGAGTAAAACAAACAAATAAATCAA  192

Qry 161      AACTTTCAACAACGGATCTCTTGGTTCTGGCATCGATGAAGAACGCAGCAAAATGCGATA  220
             ||||||||||||||||||||||||||||||||||||||||||||||||||||||||||||
Ref 193      AACTTTCAACAACGGATCTCTTGGTTCTGGCATCGATGAAGAACGCAGCAAAATGCGATA  252

Qry 221      AGTAATGTGAATTGCAGAATTCAGTGAATCATCGAATCTTTGAACGCACATTGCGCCCGC  280
             ||||||||||||||||||||||||||||||||||||||||||||||||||||||||||||
Ref 253      AGTAATGTGAATTGCAGAATTCAGTGAATCATCGAATCTTTGAACGCACATTGCGCCCGC  312

Qry 281      CAGTATTCTGGCGGGCATGCCTGTTCGAGCGTCATTTCAACCCTCAAGCTCAGCTTGGTG  340
             ||||||||||||||||||||||||||||||||||||||||||||||||||||||||||||
Ref 313      CAGTATTCTGGCGGGCATGCCTGTTCGAGCGTCATTTCAACCCTCAAGCTCAGCTTGGTG  372

Qry 341      TTGGGACTCGCGGTAACCCGCGTTCCCCAAATCGATTGGCGGTCACGTCGAGCTTCCATA  400
             ||||||||||||||||||||||||||||||||||||||||||||||||||||||||||||
Ref 373      TTGGGACTCGCGGTAACCCGCGTTCCCCAAATCGATTGGCGGTCACGTCGAGCTTCCATA  432

Qry 401      GCGTAGTAATCATACACCTCGTTACTGGTAATCGTCGCGGCCACGCCGTAAAACCCCAAC  460
             ||||||||||||||||||||||||||||||||||||||||||||||||||||||||||||
Ref 433      GCGTAGTAATCATACACCTCGTTACTGGTAATCGTCGCGGCCACGCCGTAAAACCCCAAC  492

Qry 461      TTCTGAATGTTGACCTCGGATCAGGTAGGAATAC  494
             ||||||||||||||||||||||||||||||||||
Ref 493      TTCTGAATGTTGACCTCGGATCAGGTAGGAATAC  526
```

Reference sequence:
Fusarium incarnatum-equiseti species complex (NRRL 34034; MLST type: 1-c)
GQ505725
Fusarium incarnatum-equiseti species complex (NRRL 34034; MLST type: 1-c), Fusarium incarnatum-equiseti species complex (NRRL 34034; MLST type: 1-c), 1-c, USA AZ, Human leg, n7: 28S ribosomal RNA large subunit (28S - LSU)

Sequence length: 1132

Similarity: 24/24 [100.000 %], Gaps: 0 [0.000 %], Coverage: 24/24 [100.000 %]
Score: 39.6241, Probability: 0.00194015, Direction: +/+

```
Qry 2          GCGGAGGGATCATTACCGAGTTTA  25
               ||||||||||||||||||||||||
Ref 29         GCGGAGGGATCATTACCGAGTTTA  52
```

2	Fusarium incarnatum-equiseti species complex (NRRL 28029; MLST type: 3-b) GQ505691 Fusarium incarnatum-equiseti species complex (NRRL 28029; MLST type: 3-b), Fusarium incarnatum-equiseti species complex (NRRL 28029; MLST type: 3-b), 3-b, USA CA, Human eye, n4: Internal transcribed spacers (ITS1 and ITS2)	721.158	0	100	2	96.761	+/+	****

Alignment

Reference sequence:
Fusarium incarnatum-equiseti species complex (NRRL 28029; MLST type: 3-b)
GQ505691
Fusarium incarnatum-equiseti species complex (NRRL 28029; MLST type: 3-b), Fusarium incarnatum-equiseti species complex (NRRL 28029; MLST type: 3-b), 3-b, USA CA, Human eye, n4: Internal transcribed spacers (ITS1 and ITS2)

Sequence length: 1132

Similarity: 454/454 [100.000 %], Gaps: 0 [0.000 %], Coverage: 454/454 [100.000 %]
Score: 721.158, Probability: 0, Direction: +/+

```
Qry 41         ACATACCTATACGTTGCCTCGGCGGATCAGCCCGCGCCCCGTAAAAAGGGACGGCCCGCC  100
               ||||||||||||||||||||||||||||||||||||||||||||||||||||||||||||
Ref 73         ACATACCTATACGTTGCCTCGGCGGATCAGCCCGCGCCCCGTAAAAAGGGACGGCCCGCC  132

Qry 101        CGAGGACCCCTAAACTCTGTTTTTAGTGGAACTTCTGAGTAAAACAAACAAATAAATCAA  160
               ||||||||||||||||||||||||||||||||||||||||||||||||||||||||||||
Ref 133        CGAGGACCCCTAAACTCTGTTTTTAGTGGAACTTCTGAGTAAAACAAACAAATAAATCAA  192

Qry 161        AACTTTCAACAACGGATCTCTTGGTTCTGGCATCGATGAAGAACGCAGCAAAATGCGATA  220
               ||||||||||||||||||||||||||||||||||||||||||||||||||||||||||||
Ref 193        AACTTTCAACAACGGATCTCTTGGTTCTGGCATCGATGAAGAACGCAGCAAAATGCGATA  252

Qry 221        AGTAATGTGAATTGCAGAATTCAGTGAATCATCGAATCTTTGAACGCACATTGCGCCCGC  280
               ||||||||||||||||||||||||||||||||||||||||||||||||||||||||||||
Ref 253        AGTAATGTGAATTGCAGAATTCAGTGAATCATCGAATCTTTGAACGCACATTGCGCCCGC  312

Qry 281        CAGTATTCTGGCGGGCATGCCTGTTCGAGCGTCATTTCAACCCTCAAGCTCAGCTTGGTG  340
               ||||||||||||||||||||||||||||||||||||||||||||||||||||||||||||
Ref 313        CAGTATTCTGGCGGGCATGCCTGTTCGAGCGTCATTTCAACCCTCAAGCTCAGCTTGGTG  372

Qry 341        TTGGGACTCGCGGTAACCCGCGTTCCCCAAATCGATTGGCGGTCACGTCGAGCTTCCATA  400
               ||||||||||||||||||||||||||||||||||||||||||||||||||||||||||||
Ref 373        TTGGGACTCGCGGTAACCCGCGTTCCCCAAATCGATTGGCGGTCACGTCGAGCTTCCATA  432

Qry 401        GCGTAGTAATCATACACCTCGTTACTGGTAATCGTCGCGGCCACGCCGTAAAACCCCAAC  460
               ||||||||||||||||||||||||||||||||||||||||||||||||||||||||||||
Ref 433        GCGTAGTAATCATACACCTCGTTACTGGTAATCGTCGCGGCCACGCCGTAAAACCCCAAC  492

Qry 461        TTCTGAATGTTGACCTCGGATCAGGTAGGAATAC  494
               ||||||||||||||||||||||||||||||||||
Ref 493        TTCTGAATGTTGACCTCGGATCAGGTAGGAATAC  526
```

Reference sequence:
Fusarium incarnatum-equiseti species complex (NRRL 28029; MLST type: 3-b)
GQ505691
Fusarium incarnatum-equiseti species complex (NRRL 28029; MLST type: 3-b), Fusarium incarnatum-equiseti species complex (NRRL 28029; MLST type: 3-b), 3-b, USA CA, Human eye, n4: Internal transcribed spacers (ITS1 and ITS2)

Sequence length: 1132

Similarity: 24/24 [100.000 %], Gaps: 0 [0.000 %], Coverage: 24/24 [100.000 %]
Score: 39.6241, Probability: 0.00194015, Direction: +/+

```
Qry 2          GCGGAGGGATCATTACCGAGTTTA  25
               ||||||||||||||||||||||||
Ref 29         GCGGAGGGATCATTACCGAGTTTA  52
```

3	Fusarium incarnatum-equiseti species complex (NRRL 28029; MLST type: 3-b) GQ505691 Fusarium incarnatum-equiseti species complex (NRRL 28029; MLST type: 3-b), Fusarium incarnatum-equiseti species complex (NRRL 28029; MLST type: 3-b), 3-b, USA CA, Human eye, n7: 28S ribosomal RNA large subunit (28S - LSU)	721.158	0	100	2	96.761	+/+	****

Alignment

Reference sequence:
Fusarium incarnatum-equiseti species complex (NRRL 28029; MLST type: 3-b)
GQ505691
Fusarium incarnatum-equiseti species complex (NRRL 28029; MLST type: 3-b), Fusarium incarnatum-equiseti species complex (NRRL 28029; MLST type: 3-b), 3-b, USA CA, Human eye, n7: 28S ribosomal RNA large subunit (28S - LSU)

Sequence length: 1132

Similarity: 454/454 [100.000 %], Gaps: 0 [0.000 %], Coverage: 454/454 [100.000 %]
Score: 721.158, Probability: 0, Direction: +/+

```
Qry 41         ACATACCTATACGTTGCCTCGGCGGATCAGCCCGCGCCCCGTAAAAAGGGACGGCCCGCC  100
               ||||||||||||||||||||||||||||||||||||||||||||||||||||||||||||
Ref 73         ACATACCTATACGTTGCCTCGGCGGATCAGCCCGCGCCCCGTAAAAAGGGACGGCCCGCC  132

Qry 101        CGAGGACCCCTAAACTCTGTTTTTAGTGGAACTTCTGAGTAAAACAAACAAATAAATCAA  160
               ||||||||||||||||||||||||||||||||||||||||||||||||||||||||||||
Ref 133        CGAGGACCCCTAAACTCTGTTTTTAGTGGAACTTCTGAGTAAAACAAACAAATAAATCAA  192

Qry 161        AACTTTCAACAACGGATCTCTTGGTTCTGGCATCGATGAAGAACGCAGCAAAATGCGATA  220
               ||||||||||||||||||||||||||||||||||||||||||||||||||||||||||||
Ref 193        AACTTTCAACAACGGATCTCTTGGTTCTGGCATCGATGAAGAACGCAGCAAAATGCGATA  252

Qry 221        AGTAATGTGAATTGCAGAATTCAGTGAATCATCGAATCTTTGAACGCACATTGCGCCCGC  280
               ||||||||||||||||||||||||||||||||||||||||||||||||||||||||||||
Ref 253        AGTAATGTGAATTGCAGAATTCAGTGAATCATCGAATCTTTGAACGCACATTGCGCCCGC  312

Qry 281        CAGTATTCTGGCGGGCATGCCTGTTCGAGCGTCATTTCAACCCTCAAGCTCAGCTTGGTG  340
               ||||||||||||||||||||||||||||||||||||||||||||||||||||||||||||
Ref 313        CAGTATTCTGGCGGGCATGCCTGTTCGAGCGTCATTTCAACCCTCAAGCTCAGCTTGGTG  372

Qry 341        TTGGGACTCGCGGTAACCCGCGTTCCCCAAATCGATTGGCGGTCACGTCGAGCTTCCATA  400
               ||||||||||||||||||||||||||||||||||||||||||||||||||||||||||||
Ref 373        TTGGGACTCGCGGTAACCCGCGTTCCCCAAATCGATTGGCGGTCACGTCGAGCTTCCATA  432

Qry 401        GCGTAGTAATCATACACCTCGTTACTGGTAATCGTCGCGGCCACGCCGTAAAACCCCAAC  460
               ||||||||||||||||||||||||||||||||||||||||||||||||||||||||||||
Ref 433        GCGTAGTAATCATACACCTCGTTACTGGTAATCGTCGCGGCCACGCCGTAAAACCCCAAC  492

Qry 461        TTCTGAATGTTGACCTCGGATCAGGTAGGAATAC  494
               ||||||||||||||||||||||||||||||||||
Ref 493        TTCTGAATGTTGACCTCGGATCAGGTAGGAATAC  526
```

Reference sequence:
Fusarium incarnatum-equiseti species complex (NRRL 28029; MLST type: 3-b)
GQ505691
Fusarium incarnatum-equiseti species complex (NRRL 28029; MLST type: 3-b), Fusarium incarnatum-equiseti species complex (NRRL 28029; MLST type: 3-b), 3-b, USA CA, Human

eye, n7: 28S ribosomal RNA large subunit (28S - LSU)

Sequence length: 1132

Similarity: 24/24 [100.000 %], Gaps: 0 [0.000 %], Coverage: 24/24 [100.000 %]
Score: 39.6241, Probability: 0.00194015, Direction: +/+

```
Qry 2          GCGGAGGGATCATTACCGAGTTTA  25
               ||||||||||||||||||||||||
Ref 29         GCGGAGGGATCATTACCGAGTTTA  52
```

4	Fusarium incarnatum-equiseti species complex (NRRL 29134; MLST type: 9-a) GQ505694 Fusarium incarnatum-equiseti species complex (NRRL 29134; MLST type: 9-a), Fusarium incarnatum-equiseti species complex (NRRL 29134; MLST type: 9-a), 9-a, Australia, Pasture soil, n4: Internal transcribed spacers (ITS1 and ITS2)	721.158	0	100	2	96.761	+/+	****

Alignment

Reference sequence:
Fusarium incarnatum-equiseti species complex (NRRL 29134; MLST type: 9-a)
GQ505694
Fusarium incarnatum-equiseti species complex (NRRL 29134; MLST type: 9-a), Fusarium incarnatum-equiseti species complex (NRRL 29134; MLST type: 9-a), 9-a, Australia, Pasture soil, n4: Internal transcribed spacers (ITS1 and ITS2)

Sequence length: 1132

Similarity: 454/454 [100.000 %], Gaps: 0 [0.000 %], Coverage: 454/454 [100.000 %]
Score: 721.158, Probability: 0, Direction: +/+

```
Qry 41         ACATACCTATACGTTGCCTCGGCGGATCAGCCCGCGCCCCGTAAAAAGGGACGGCCCGCC  100
               ||||||||||||||||||||||||||||||||||||||||||||||||||||||||||||
Ref 73         ACATACCTATACGTTGCCTCGGCGGATCAGCCCGCGCCCCGTAAAAAGGGACGGCCCGCC  132

Qry 101        CGAGGACCCCTAAACTCTGTTTTTAGTGGAACTTCTGAGTAAAACAAACAAATAAATCAA  160
               ||||||||||||||||||||||||||||||||||||||||||||||||||||||||||||
Ref 133        CGAGGACCCCTAAACTCTGTTTTTAGTGGAACTTCTGAGTAAAACAAACAAATAAATCAA  192

Qry 161        AACTTTCAACAACGGATCTCTTGGTTCTGGCATCGATGAAGAACGCAGCAAAATGCGATA  220
               ||||||||||||||||||||||||||||||||||||||||||||||||||||||||||||
Ref 193        AACTTTCAACAACGGATCTCTTGGTTCTGGCATCGATGAAGAACGCAGCAAAATGCGATA  252

Qry 221        AGTAATGTGAATTGCAGAATTCAGTGAATCATCGAATCTTTGAACGCACATTGCGCCCGC  280
               ||||||||||||||||||||||||||||||||||||||||||||||||||||||||||||
Ref 253        AGTAATGTGAATTGCAGAATTCAGTGAATCATCGAATCTTTGAACGCACATTGCGCCCGC  312

Qry 281        CAGTATTCTGGCGGGCATGCCTGTTCGAGCGTCATTTCAACCCTCAAGCTCAGCTTGGTG  340
               ||||||||||||||||||||||||||||||||||||||||||||||||||||||||||||
Ref 313        CAGTATTCTGGCGGGCATGCCTGTTCGAGCGTCATTTCAACCCTCAAGCTCAGCTTGGTG  372

Qry 341        TTGGGACTCGCGGTAACCCGCGTTCCCCAAATCGATTGGCGGTCACGTCGAGCTTCCATA  400
               ||||||||||||||||||||||||||||||||||||||||||||||||||||||||||||
Ref 373        TTGGGACTCGCGGTAACCCGCGTTCCCCAAATCGATTGGCGGTCACGTCGAGCTTCCATA  432

Qry 401        GCGTAGTAATCATACACCTCGTTACTGGTAATCGTCGCGGCCACGCCGTAAAACCCCAAC  460
               ||||||||||||||||||||||||||||||||||||||||||||||||||||||||||||
Ref 433        GCGTAGTAATCATACACCTCGTTACTGGTAATCGTCGCGGCCACGCCGTAAAACCCCAAC  492

Qry 461        TTCTGAATGTTGACCTCGGATCAGGTAGGAATAC  494
               ||||||||||||||||||||||||||||||||||
Ref 493        TTCTGAATGTTGACCTCGGATCAGGTAGGAATAC  526
```

Reference sequence:
Fusarium incarnatum-equiseti species complex (NRRL 29134; MLST type: 9-a)

GQ505694
Fusarium incarnatum-equiseti species complex (NRRL 29134; MLST type: 9-a), Fusarium incarnatum-equiseti species complex (NRRL 29134; MLST type: 9-a), 9-a, Australia, Pasture soil, n4: Internal transcribed spacers (ITS1 and ITS2)

Sequence length: 1132

Similarity: 24/24 [100.000 %], Gaps: 0 [0.000 %], Coverage: 24/24 [100.000 %]
Score: 39.6241, Probability: 0.00194015, Direction: +/+

```
Qry 2          GCGGAGGGATCATTACCGAGTTTA  25
               ||||||||||||||||||||||||
Ref 29         GCGGAGGGATCATTACCGAGTTTA  52
```

5	Fusarium incarnatum-equiseti species complex (NRRL 29134; MLST type: 9-a) GQ505694 Fusarium incarnatum-equiseti species complex (NRRL 29134; MLST type: 9-a), Fusarium incarnatum-equiseti species complex (NRRL 29134; MLST type: 9-a), 9-a, Australia, Pasture soil, n7: 28S ribosomal RNA large subunit (28S - LSU)	721.158	0	100	2	96.761	+/+	****

Alignment

Reference sequence:
Fusarium incarnatum-equiseti species complex (NRRL 29134; MLST type: 9-a)
GQ505694
Fusarium incarnatum-equiseti species complex (NRRL 29134; MLST type: 9-a), Fusarium incarnatum-equiseti species complex (NRRL 29134; MLST type: 9-a), 9-a, Australia, Pasture soil, n7: 28S ribosomal RNA large subunit (28S - LSU)

Sequence length: 1132

Similarity: 454/454 [100.000 %], Gaps: 0 [0.000 %], Coverage: 454/454 [100.000 %]
Score: 721.158, Probability: 0, Direction: +/+

```
Qry 41         ACATACCTATACGTTGCCTCGGCGGATCAGCCCGCGCCCCGTAAAAAGGGACGGCCCGCC  100
               ||||||||||||||||||||||||||||||||||||||||||||||||||||||||||||
Ref 73         ACATACCTATACGTTGCCTCGGCGGATCAGCCCGCGCCCCGTAAAAAGGGACGGCCCGCC  132

Qry 101        CGAGGACCCCTAAACTCTGTTTTTAGTGGAACTTCTGAGTAAAACAAACAAATAAATCAA  160
               ||||||||||||||||||||||||||||||||||||||||||||||||||||||||||||
Ref 133        CGAGGACCCCTAAACTCTGTTTTTAGTGGAACTTCTGAGTAAAACAAACAAATAAATCAA  192

Qry 161        AACTTTCAACAACGGATCTCTTGGTTCTGGCATCGATGAAGAACGCAGCAAAATGCGATA  220
               ||||||||||||||||||||||||||||||||||||||||||||||||||||||||||||
Ref 193        AACTTTCAACAACGGATCTCTTGGTTCTGGCATCGATGAAGAACGCAGCAAAATGCGATA  252

Qry 221        AGTAATGTGAATTGCAGAATTCAGTGAATCATCGAATCTTTGAACGCACATTGCGCCCGC  280
               ||||||||||||||||||||||||||||||||||||||||||||||||||||||||||||
Ref 253        AGTAATGTGAATTGCAGAATTCAGTGAATCATCGAATCTTTGAACGCACATTGCGCCCGC  312

Qry 281        CAGTATTCTGGCGGGCATGCCTGTTCGAGCGTCATTTCAACCCTCAAGCTCAGCTTGGTG  340
               ||||||||||||||||||||||||||||||||||||||||||||||||||||||||||||
Ref 313        CAGTATTCTGGCGGGCATGCCTGTTCGAGCGTCATTTCAACCCTCAAGCTCAGCTTGGTG  372

Qry 341        TTGGGACTCGCGGTAACCCGCGTTCCCCAAATCGATTGGCGGTCACGTCGAGCTTCCATA  400
               ||||||||||||||||||||||||||||||||||||||||||||||||||||||||||||
Ref 373        TTGGGACTCGCGGTAACCCGCGTTCCCCAAATCGATTGGCGGTCACGTCGAGCTTCCATA  432

Qry 401        GCGTAGTAATCATACACCTCGTTACTGGTAATCGTCGCGGCCACGCCGTAAAACCCCAAC  460
               ||||||||||||||||||||||||||||||||||||||||||||||||||||||||||||
Ref 433        GCGTAGTAATCATACACCTCGTTACTGGTAATCGTCGCGGCCACGCCGTAAAACCCCAAC  492

Qry 461        TTCTGAATGTTGACCTCGGATCAGGTAGGAATAC  494
               ||||||||||||||||||||||||||||||||||
Ref 493        TTCTGAATGTTGACCTCGGATCAGGTAGGAATAC  526
```

Reference sequence:
[Fusarium incarnatum-equiseti species complex (NRRL 29134; MLST type: 9-a) GQ505694]
Fusarium incarnatum-equiseti species complex (NRRL 29134; MLST type: 9-a), Fusarium incarnatum-equiseti species complex (NRRL 29134; MLST type: 9-a), 9-a, Australia, Pasture soil, n7: 28S ribosomal RNA large subunit (28S - LSU)

Sequence length: 1132

Similarity: 24/24 [100.000 %], Gaps: 0 [0.000 %], Coverage: 24/24 [100.000 %]
Score: 39.6241, Probability: 0.00194015, Direction: +/+

```
Qry 2          GCGGAGGGATCATTACCGAGTTTA  25
               ||||||||||||||||||||||||
Ref 29         GCGGAGGGATCATTACCGAGTTTA  52
```

6	Fusarium incarnatum-equiseti species complex (NRRL 32997; MLST type: 7-a) GQ505713 Fusarium incarnatum-equiseti species complex (NRRL 32997; MLST type: 7-a), Fusarium incarnatum-equiseti species complex (NRRL 32997; MLST type: 7-a), 7-a, USA CO, Human toenail, n4: Internal transcribed spacers (ITS1 and ITS2)	721.158	0	100	2	96.761	+/+	****

Alignment

Reference sequence:
[Fusarium incarnatum-equiseti species complex (NRRL 32997; MLST type: 7-a) GQ505713]
Fusarium incarnatum-equiseti species complex (NRRL 32997; MLST type: 7-a), Fusarium incarnatum-equiseti species complex (NRRL 32997; MLST type: 7-a), 7-a, USA CO, Human toenail, n4: Internal transcribed spacers (ITS1 and ITS2)

Sequence length: 1132

Similarity: 454/454 [100.000 %], Gaps: 0 [0.000 %], Coverage: 454/454 [100.000 %]
Score: 721.158, Probability: 0, Direction: +/+

```
Qry 41         ACATACCTATACGTTGCCTCGGCGGATCAGCCCGCGCCCCGTAAAAAGGGACGGCCCGCC  100
               ||||||||||||||||||||||||||||||||||||||||||||||||||||||||||||
Ref 73         ACATACCTATACGTTGCCTCGGCGGATCAGCCCGCGCCCCGTAAAAAGGGACGGCCCGCC  132

Qry 101        CGAGGACCCCTAAACTCTGTTTTTAGTGGAACTTCTGAGTAAAACAAACAAATAAATCAA  160
               ||||||||||||||||||||||||||||||||||||||||||||||||||||||||||||
Ref 133        CGAGGACCCCTAAACTCTGTTTTTAGTGGAACTTCTGAGTAAAACAAACAAATAAATCAA  192

Qry 161        AACTTTCAACAACGGATCTCTTGGTTCTGGCATCGATGAAGAACGCAGCAAAATGCGATA  220
               ||||||||||||||||||||||||||||||||||||||||||||||||||||||||||||
Ref 193        AACTTTCAACAACGGATCTCTTGGTTCTGGCATCGATGAAGAACGCAGCAAAATGCGATA  252

Qry 221        AGTAATGTGAATTGCAGAATTCAGTGAATCATCGAATCTTTGAACGCACATTGCGCCCGC  280
               ||||||||||||||||||||||||||||||||||||||||||||||||||||||||||||
Ref 253        AGTAATGTGAATTGCAGAATTCAGTGAATCATCGAATCTTTGAACGCACATTGCGCCCGC  312

Qry 281        CAGTATTCTGGCGGGCATGCCTGTTCGAGCGTCATTTCAACCCTCAAGCTCAGCTTGGTG  340
               ||||||||||||||||||||||||||||||||||||||||||||||||||||||||||||
Ref 313        CAGTATTCTGGCGGGCATGCCTGTTCGAGCGTCATTTCAACCCTCAAGCTCAGCTTGGTG  372

Qry 341        TTGGGACTCGCGGTAACCCGCGTTCCCCAAATCGATTGGCGGTCACGTCGAGCTTCCATA  400
               ||||||||||||||||||||||||||||||||||||||||||||||||||||||||||||
Ref 373        TTGGGACTCGCGGTAACCCGCGTTCCCCAAATCGATTGGCGGTCACGTCGAGCTTCCATA  432

Qry 401        GCGTAGTAATCATACACCTCGTTACTGGTAATCGTCGCGGCCACGCCGTAAAACCCCAAC  460
               ||||||||||||||||||||||||||||||||||||||||||||||||||||||||||||
Ref 433        GCGTAGTAATCATACACCTCGTTACTGGTAATCGTCGCGGCCACGCCGTAAAACCCCAAC  492
```

```
Qry 461        TTCTGAATGTTGACCTCGGATCAGGTAGGAATAC  494
               ||||||||||||||||||||||||||||||||||
Ref 493        TTCTGAATGTTGACCTCGGATCAGGTAGGAATAC  526
```

Reference sequence:
Fusarium incarnatum-equiseti species complex (NRRL 32997; MLST type: 7-a)
GQ505713
Fusarium incarnatum-equiseti species complex (NRRL 32997; MLST type: 7-a), Fusarium incarnatum-equiseti species complex (NRRL 32997; MLST type: 7-a), 7-a, USA CO, Human toenail, n4: Internal transcribed spacers (ITS1 and ITS2)

Sequence length: 1132

Similarity: 24/24 [100.000 %], Gaps: 0 [0.000 %], Coverage: 24/24 [100.000 %]
Score: 39.6241, Probability: 0.00194015, Direction: +/+

```
Qry 2          GCGGAGGGATCATTACCGAGTTTA  25
               ||||||||||||||||||||||||
Ref 29         GCGGAGGGATCATTACCGAGTTTA  52
```

7	Fusarium incarnatum-equiseti species complex (NRRL 32997; MLST type: 7-a) GQ505713 Fusarium incarnatum-equiseti species complex (NRRL 32997; MLST type: 7-a), Fusarium incarnatum-equiseti species complex (NRRL 32997; MLST type: 7-a), 7-a, USA CO, Human toenail, n7: 28S ribosomal RNA large subunit (28S - LSU)	721.158	0	100	2	96.761	+/+	****

Alignment

Reference sequence:
Fusarium incarnatum-equiseti species complex (NRRL 32997; MLST type: 7-a)
GQ505713
Fusarium incarnatum-equiseti species complex (NRRL 32997; MLST type: 7-a), Fusarium incarnatum-equiseti species complex (NRRL 32997; MLST type: 7-a), 7-a, USA CO, Human toenail, n7: 28S ribosomal RNA large subunit (28S - LSU)

Sequence length: 1132

Similarity: 454/454 [100.000 %], Gaps: 0 [0.000 %], Coverage: 454/454 [100.000 %]
Score: 721.158, Probability: 0, Direction: +/+

```
Qry 41         ACATACCTATACGTTGCCTCGGCGGATCAGCCCGCGCCCCGTAAAAAGGGACGGCCCGCC  100
               ||||||||||||||||||||||||||||||||||||||||||||||||||||||||||||
Ref 73         ACATACCTATACGTTGCCTCGGCGGATCAGCCCGCGCCCCGTAAAAAGGGACGGCCCGCC  132

Qry 101        CGAGGACCCCTAAACTCTGTTTTTAGTGGAACTTCTGAGTAAAACAAACAAATAAATCAA  160
               ||||||||||||||||||||||||||||||||||||||||||||||||||||||||||||
Ref 133        CGAGGACCCCTAAACTCTGTTTTTAGTGGAACTTCTGAGTAAAACAAACAAATAAATCAA  192

Qry 161        AACTTTCAACAACGGATCTCTTGGTTCTGGCATCGATGAAGAACGCAGCAAAATGCGATA  220
               ||||||||||||||||||||||||||||||||||||||||||||||||||||||||||||
Ref 193        AACTTTCAACAACGGATCTCTTGGTTCTGGCATCGATGAAGAACGCAGCAAAATGCGATA  252

Qry 221        AGTAATGTGAATTGCAGAATTCAGTGAATCATCGAATCTTTGAACGCACATTGCGCCCGC  280
               ||||||||||||||||||||||||||||||||||||||||||||||||||||||||||||
Ref 253        AGTAATGTGAATTGCAGAATTCAGTGAATCATCGAATCTTTGAACGCACATTGCGCCCGC  312

Qry 281        CAGTATTCTGGCGGGCATGCCTGTTCGAGCGTCATTTCAACCCTCAAGCTCAGCTTGGTG  340
               ||||||||||||||||||||||||||||||||||||||||||||||||||||||||||||
Ref 313        CAGTATTCTGGCGGGCATGCCTGTTCGAGCGTCATTTCAACCCTCAAGCTCAGCTTGGTG  372

Qry 341        TTGGGACTCGCGGTAACCCGCGTTCCCCAAATCGATTGGCGGTCACGTCGAGCTTCCATA  400
               ||||||||||||||||||||||||||||||||||||||||||||||||||||||||||||
Ref 373        TTGGGACTCGCGGTAACCCGCGTTCCCCAAATCGATTGGCGGTCACGTCGAGCTTCCATA  432

Qry 401        GCGTAGTAATCATACACCTCGTTACTGGTAATCGTCGCGGCCACGCCGTAAAACCCCAAC  460
```

```
                ||||||||||||||||||||||||||||||||||||||||||||||||||||||||||||
Ref 433         GCGTAGTAATCATACACCTCGTTACTGGTAATCGTCGCGGCCACGCCGTAAAACCCCAAC  492

Qry 461         TTCTGAATGTTGACCTCGGATCAGGTAGGAATAC  494
                ||||||||||||||||||||||||||||||||||
Ref 493         TTCTGAATGTTGACCTCGGATCAGGTAGGAATAC  526
```

Reference sequence:
[Fusarium incarnatum-equiseti species complex (NRRL 32997; MLST type: 7-a)](GQ505713)
[GQ505713](GQ505713)
Fusarium incarnatum-equiseti species complex (NRRL 32997; MLST type: 7-a), Fusarium incarnatum-equiseti species complex (NRRL 32997; MLST type: 7-a), 7-a, USA CO, Human toenail, n7: 28S ribosomal RNA large subunit (28S - LSU)

Sequence length: 1132

Similarity: 24/24 [100.000 %], Gaps: 0 [0.000 %], Coverage: 24/24 [100.000 %]
Score: 39.6241, Probability: 0.00194015, Direction: +/+

```
Qry 2           GCGGAGGGATCATTACCGAGTTTA  25
                ||||||||||||||||||||||||
Ref 29          GCGGAGGGATCATTACCGAGTTTA  52
```

8	Fusarium incarnatum-equiseti species complex (NRRL 34034; MLST type: 1-c) GQ505725 Fusarium incarnatum-equiseti species complex (NRRL 34034; MLST type: 1-c), Fusarium incarnatum-equiseti species complex (NRRL 34034; MLST type: 1-c), 1-c, USA AZ, Human leg, n4: Internal transcribed spacers (ITS1 and ITS2)	721.158	0	100	2	96.761	+/+	****

Alignment

Reference sequence:
[Fusarium incarnatum-equiseti species complex (NRRL 34034; MLST type: 1-c)](GQ505725)
[GQ505725](GQ505725)
Fusarium incarnatum-equiseti species complex (NRRL 34034; MLST type: 1-c), Fusarium incarnatum-equiseti species complex (NRRL 34034; MLST type: 1-c), 1-c, USA AZ, Human leg, n4: Internal transcribed spacers (ITS1 and ITS2)

Sequence length: 1132

Similarity: 454/454 [100.000 %], Gaps: 0 [0.000 %], Coverage: 454/454 [100.000 %]
Score: 721.158, Probability: 0, Direction: +/+

```
Qry 41          ACATACCTATACGTTGCCTCGGCGGATCAGCCCGCGCCCCGTAAAAAGGGACGGCCCGCC  100
                ||||||||||||||||||||||||||||||||||||||||||||||||||||||||||||
Ref 73          ACATACCTATACGTTGCCTCGGCGGATCAGCCCGCGCCCCGTAAAAAGGGACGGCCCGCC  132

Qry 101         CGAGGACCCCTAAACTCTGTTTTTAGTGGAACTTCTGAGTAAAACAAACAAATAAATCAA  160
                ||||||||||||||||||||||||||||||||||||||||||||||||||||||||||||
Ref 133         CGAGGACCCCTAAACTCTGTTTTTAGTGGAACTTCTGAGTAAAACAAACAAATAAATCAA  192

Qry 161         AACTTTCAACAACGGATCTCTTGGTTCTGGCATCGATGAAGAACGCAGCAAAATGCGATA  220
                ||||||||||||||||||||||||||||||||||||||||||||||||||||||||||||
Ref 193         AACTTTCAACAACGGATCTCTTGGTTCTGGCATCGATGAAGAACGCAGCAAAATGCGATA  252

Qry 221         AGTAATGTGAATTGCAGAATTCAGTGAATCATCGAATCTTTGAACGCACATTGCGCCCGC  280
                ||||||||||||||||||||||||||||||||||||||||||||||||||||||||||||
Ref 253         AGTAATGTGAATTGCAGAATTCAGTGAATCATCGAATCTTTGAACGCACATTGCGCCCGC  312

Qry 281         CAGTATTCTGGCGGGCATGCCTGTTCGAGCGTCATTTCAACCCTCAAGCTCAGCTTGGTG  340
                ||||||||||||||||||||||||||||||||||||||||||||||||||||||||||||
Ref 313         CAGTATTCTGGCGGGCATGCCTGTTCGAGCGTCATTTCAACCCTCAAGCTCAGCTTGGTG  372

Qry 341         TTGGGACTCGCGGTAACCCGCGTTCCCCAAATCGATTGGCGGTCACGTCGAGCTTCCATA  400
                ||||||||||||||||||||||||||||||||||||||||||||||||||||||||||||
```

```
Ref 373       TTGGGACTCGCGGTAACCCGCGTTCCCCAAATCGATTGGCGGTCACGTCGAGCTTCCATA  432

Qry 401       GCGTAGTAATCATACACCTCGTTACTGGTAATCGTCGCGGCCACGCCGTAAAACCCCAAC  460
              ||||||||||||||||||||||||||||||||||||||||||||||||||||||||||||
Ref 433       GCGTAGTAATCATACACCTCGTTACTGGTAATCGTCGCGGCCACGCCGTAAAACCCCAAC  492

Qry 461       TTCTGAATGTTGACCTCGGATCAGGTAGGAATAC  494
              ||||||||||||||||||||||||||||||||||
Ref 493       TTCTGAATGTTGACCTCGGATCAGGTAGGAATAC  526
```

Reference sequence:
Fusarium incarnatum-equiseti species complex (NRRL 34034; MLST type: 1-c)
GQ505725
Fusarium incarnatum-equiseti species complex (NRRL 34034; MLST type: 1-c), Fusarium incarnatum-equiseti species complex (NRRL 34034; MLST type: 1-c), 1-c, USA AZ, Human leg, n4: Internal transcribed spacers (ITS1 and ITS2)

Sequence length: 1132

Similarity: 24/24 [100.000 %], Gaps: 0 [0.000 %], Coverage: 24/24 [100.000 %]
Score: 39.6241, Probability: 0.00194015, Direction: +/+

```
Qry 2         GCGGAGGGATCATTACCGAGTTTA  25
              ||||||||||||||||||||||||
Ref 29        GCGGAGGGATCATTACCGAGTTTA  52
```

9	Fusarium incarnatum-equiseti species complex (NRRL 20423; MLST type: 4-a) GQ505682 Fusarium incarnatum-equiseti species complex (NRRL 20423; MLST type: 4-a), Fusarium incarnatum-equiseti species complex (NRRL 20423; MLST type: 4-a), 4-a, India, Lizard skin, n7: 28S ribosomal RNA large subunit (28S - LSU)	721.158	0	100	2	96.761	+/+	****

Alignment

Reference sequence:
Fusarium incarnatum-equiseti species complex (NRRL 20423; MLST type: 4-a)
GQ505682
Fusarium incarnatum-equiseti species complex (NRRL 20423; MLST type: 4-a), Fusarium incarnatum-equiseti species complex (NRRL 20423; MLST type: 4-a), 4-a, India, Lizard skin, n7: 28S ribosomal RNA large subunit (28S - LSU)

Sequence length: 1132

Similarity: 454/454 [100.000 %], Gaps: 0 [0.000 %], Coverage: 454/454 [100.000 %]
Score: 721.158, Probability: 0, Direction: +/+

```
Qry 41        ACATACCTATACGTTGCCTCGGCGGATCAGCCCGCGCCCCGTAAAAAGGGACGGCCCGCC  100
              ||||||||||||||||||||||||||||||||||||||||||||||||||||||||||||
Ref 73        ACATACCTATACGTTGCCTCGGCGGATCAGCCCGCGCCCCGTAAAAAGGGACGGCCCGCC  132

Qry 101       CGAGGACCCCTAAACTCTGTTTTTAGTGGAACTTCTGAGTAAAACAAACAAATAAATCAA  160
              ||||||||||||||||||||||||||||||||||||||||||||||||||||||||||||
Ref 133       CGAGGACCCCTAAACTCTGTTTTTAGTGGAACTTCTGAGTAAAACAAACAAATAAATCAA  192

Qry 161       AACTTTCAACAACGGATCTCTTGGTTCTGGCATCGATGAAGAACGCAGCAAAATGCGATA  220
              ||||||||||||||||||||||||||||||||||||||||||||||||||||||||||||
Ref 193       AACTTTCAACAACGGATCTCTTGGTTCTGGCATCGATGAAGAACGCAGCAAAATGCGATA  252

Qry 221       AGTAATGTGAATTGCAGAATTCAGTGAATCATCGAATCTTTGAACGCACATTGCGCCCGC  280
              ||||||||||||||||||||||||||||||||||||||||||||||||||||||||||||
Ref 253       AGTAATGTGAATTGCAGAATTCAGTGAATCATCGAATCTTTGAACGCACATTGCGCCCGC  312

Qry 281       CAGTATTCTGGCGGGCATGCCTGTTCGAGCGTCATTTCAACCCTCAAGCTCAGCTTGGTG  340
              ||||||||||||||||||||||||||||||||||||||||||||||||||||||||||||
Ref 313       CAGTATTCTGGCGGGCATGCCTGTTCGAGCGTCATTTCAACCCTCAAGCTCAGCTTGGTG  372
```

```
Qry 341        TTGGGACTCGCGGTAACCCGCGTTCCCCAAATCGATTGGCGGTCACGTCGAGCTTCCATA  400
               ||||||||||||||||||||||||||||||||||||||||||||||||||||||||||||
Ref 373        TTGGGACTCGCGGTAACCCGCGTTCCCCAAATCGATTGGCGGTCACGTCGAGCTTCCATA  432

Qry 401        GCGTAGTAATCATACACCTCGTTACTGGTAATCGTCGCGGCCACGCCGTAAAACCCCAAC  460
               ||||||||||||||||||||||||||||||||||||||||||||||||||||||||||||
Ref 433        GCGTAGTAATCATACACCTCGTTACTGGTAATCGTCGCGGCCACGCCGTAAAACCCCAAC  492

Qry 461        TTCTGAATGTTGACCTCGGATCAGGTAGGAATAC  494
               ||||||||||||||||||||||||||||||||||
Ref 493        TTCTGAATGTTGACCTCGGATCAGGTAGGAATAC  526
```

Reference sequence:
Fusarium incarnatum-equiseti species complex (NRRL 20423; MLST type: 4-a)
GQ505682
Fusarium incarnatum-equiseti species complex (NRRL 20423; MLST type: 4-a), Fusarium incarnatum-equiseti species complex (NRRL 20423; MLST type: 4-a), 4-a, India, Lizard skin, n7: 28S ribosomal RNA large subunit (28S - LSU)

Sequence length: 1132

Similarity: 24/24 [100.000 %], Gaps: 0 [0.000 %], Coverage: 24/24 [100.000 %]
Score: 39.6241, Probability: 0.00194015, Direction: +/+

```
Qry 2          GCGGAGGGATCATTACCGAGTTTA  25
               ||||||||||||||||||||||||
Ref 29         GCGGAGGGATCATTACCGAGTTTA  52
```

10	Fusarium incarnatum-equiseti species complex (NRRL 34035; MLST type: 5-d) GQ505726 Fusarium incarnatum-equiseti species complex (NRRL 34035; MLST type: 5-d), Fusarium incarnatum-equiseti species complex (NRRL 34035; MLST type: 5-d), 5-d, USA CO, Human sinus, n4: Internal transcribed spacers (ITS1 and ITS2)	721.158	0	100	2	96.761	+/+	****

Alignment

Reference sequence:
Fusarium incarnatum-equiseti species complex (NRRL 34035; MLST type: 5-d)
GQ505726
Fusarium incarnatum-equiseti species complex (NRRL 34035; MLST type: 5-d), Fusarium incarnatum-equiseti species complex (NRRL 34035; MLST type: 5-d), 5-d, USA CO, Human sinus, n4: Internal transcribed spacers (ITS1 and ITS2)

Sequence length: 1132

Similarity: 454/454 [100.000 %], Gaps: 0 [0.000 %], Coverage: 454/454 [100.000 %]
Score: 721.158, Probability: 0, Direction: +/+

```
Qry 41         ACATACCTATACGTTGCCTCGGCGGATCAGCCCGCGCCCCGTAAAAAGGGACGGCCCGCC  100
               ||||||||||||||||||||||||||||||||||||||||||||||||||||||||||||
Ref 73         ACATACCTATACGTTGCCTCGGCGGATCAGCCCGCGCCCCGTAAAAAGGGACGGCCCGCC  132

Qry 101        CGAGGACCCCTAAACTCTGTTTTTAGTGGAACTTCTGAGTAAAACAAACAAATAAATCAA  160
               ||||||||||||||||||||||||||||||||||||||||||||||||||||||||||||
Ref 133        CGAGGACCCCTAAACTCTGTTTTTAGTGGAACTTCTGAGTAAAACAAACAAATAAATCAA  192

Qry 161        AACTTTCAACAACGGATCTCTTGGTTCTGGCATCGATGAAGAACGCAGCAAAATGCGATA  220
               ||||||||||||||||||||||||||||||||||||||||||||||||||||||||||||
Ref 193        AACTTTCAACAACGGATCTCTTGGTTCTGGCATCGATGAAGAACGCAGCAAAATGCGATA  252

Qry 221        AGTAATGTGAATTGCAGAATTCAGTGAATCATCGAATCTTTGAACGCACATTGCGCCCGC  280
               ||||||||||||||||||||||||||||||||||||||||||||||||||||||||||||
Ref 253        AGTAATGTGAATTGCAGAATTCAGTGAATCATCGAATCTTTGAACGCACATTGCGCCCGC  312
```

```
Qry 281        CAGTATTCTGGCGGGCATGCCTGTTCGAGCGTCATTTCAACCCTCAAGCTCAGCTTGGTG  340
               ||||||||||||||||||||||||||||||||||||||||||||||||||||||||||||
Ref 313        CAGTATTCTGGCGGGCATGCCTGTTCGAGCGTCATTTCAACCCTCAAGCTCAGCTTGGTG  372

Qry 341        TTGGGACTCGCGGTAACCCGCGTTCCCCAAATCGATTGGCGGTCACGTCGAGCTTCCATA  400
               ||||||||||||||||||||||||||||||||||||||||||||||||||||||||||||
Ref 373        TTGGGACTCGCGGTAACCCGCGTTCCCCAAATCGATTGGCGGTCACGTCGAGCTTCCATA  432

Qry 401        GCGTAGTAATCATACACCTCGTTACTGGTAATCGTCGCGGCCACGCCGTAAAACCCCAAC  460
               ||||||||||||||||||||||||||||||||||||||||||||||||||||||||||||
Ref 433        GCGTAGTAATCATACACCTCGTTACTGGTAATCGTCGCGGCCACGCCGTAAAACCCCAAC  492

Qry 461        TTCTGAATGTTGACCTCGGATCAGGTAGGAATAC  494
               ||||||||||||||||||||||||||||||||||
Ref 493        TTCTGAATGTTGACCTCGGATCAGGTAGGAATAC  526
```

Reference sequence:
Fusarium incarnatum-equiseti species complex (NRRL 34035; MLST type: 5-d)
GQ505726
Fusarium incarnatum-equiseti species complex (NRRL 34035; MLST type: 5-d), Fusarium incarnatum-equiseti species complex (NRRL 34035; MLST type: 5-d), 5-d, USA CO, Human sinus, n4: Internal transcribed spacers (ITS1 and ITS2)

Sequence length: 1132

Similarity: 24/24 [100.000 %], Gaps: 0 [0.000 %], Coverage: 24/24 [100.000 %]
Score: 39.6241, Probability: 0.00194015, Direction: +/+

```
Qry 2          GCGGAGGGATCATTACCGAGTTTA  25
               ||||||||||||||||||||||||
Ref 29         GCGGAGGGATCATTACCGAGTTTA  52
```

11	Fusarium incarnatum-equiseti species complex (NRRL 34035; MLST type: 5-d) GQ505726 Fusarium incarnatum-equiseti species complex (NRRL 34035; MLST type: 5-d), Fusarium incarnatum-equiseti species complex (NRRL 34035; MLST type: 5-d), 5-d, USA CO, Human sinus, n7: 28S ribosomal RNA large subunit (28S - LSU)	721.158	0	100	2	96.761	+/+	****

Alignment

Reference sequence:
Fusarium incarnatum-equiseti species complex (NRRL 34035; MLST type: 5-d)
GQ505726
Fusarium incarnatum-equiseti species complex (NRRL 34035; MLST type: 5-d), Fusarium incarnatum-equiseti species complex (NRRL 34035; MLST type: 5-d), 5-d, USA CO, Human sinus, n7: 28S ribosomal RNA large subunit (28S - LSU)

Sequence length: 1132

Similarity: 454/454 [100.000 %], Gaps: 0 [0.000 %], Coverage: 454/454 [100.000 %]
Score: 721.158, Probability: 0, Direction: +/+

```
Qry 41         ACATACCTATACGTTGCCTCGGCGGATCAGCCCGCGCCCCGTAAAAAGGGACGGCCCGCC  100
               ||||||||||||||||||||||||||||||||||||||||||||||||||||||||||||
Ref 73         ACATACCTATACGTTGCCTCGGCGGATCAGCCCGCGCCCCGTAAAAAGGGACGGCCCGCC  132

Qry 101        CGAGGACCCCTAAACTCTGTTTTTAGTGGAACTTCTGAGTAAAACAAACAAATAAATCAA  160
               ||||||||||||||||||||||||||||||||||||||||||||||||||||||||||||
Ref 133        CGAGGACCCCTAAACTCTGTTTTTAGTGGAACTTCTGAGTAAAACAAACAAATAAATCAA  192

Qry 161        AACTTTCAACAACGGATCTCTTGGTTCTGGCATCGATGAAGAACGCAGCAAAATGCGATA  220
               ||||||||||||||||||||||||||||||||||||||||||||||||||||||||||||
Ref 193        AACTTTCAACAACGGATCTCTTGGTTCTGGCATCGATGAAGAACGCAGCAAAATGCGATA  252

Qry 221        AGTAATGTGAATTGCAGAATTCAGTGAATCATCGAATCTTTGAACGCACATTGCGCCCGC  280
```

```
                ||||||||||||||||||||||||||||||||||||||||||||||||||||||||||||
Ref 253         AGTAATGTGAATTGCAGAATTCAGTGAATCATCGAATCTTTGAACGCACATTGCGCCCGC  312

Qry 281         CAGTATTCTGGCGGGCATGCCTGTTCGAGCGTCATTTCAACCCTCAAGCTCAGCTTGGTG  340
                ||||||||||||||||||||||||||||||||||||||||||||||||||||||||||||
Ref 313         CAGTATTCTGGCGGGCATGCCTGTTCGAGCGTCATTTCAACCCTCAAGCTCAGCTTGGTG  372

Qry 341         TTGGGACTCGCGGTAACCCGCGTTCCCCAAATCGATTGGCGGTCACGTCGAGCTTCCATA  400
                ||||||||||||||||||||||||||||||||||||||||||||||||||||||||||||
Ref 373         TTGGGACTCGCGGTAACCCGCGTTCCCCAAATCGATTGGCGGTCACGTCGAGCTTCCATA  432

Qry 401         GCGTAGTAATCATACACCTCGTTACTGGTAATCGTCGCGGCCACGCCGTAAAACCCCAAC  460
                ||||||||||||||||||||||||||||||||||||||||||||||||||||||||||||
Ref 433         GCGTAGTAATCATACACCTCGTTACTGGTAATCGTCGCGGCCACGCCGTAAAACCCCAAC  492

Qry 461         TTCTGAATGTTGACCTCGGATCAGGTAGGAATAC  494
                ||||||||||||||||||||||||||||||||||
Ref 493         TTCTGAATGTTGACCTCGGATCAGGTAGGAATAC  526
```

Reference sequence:
Fusarium incarnatum-equiseti species complex (NRRL 34035; MLST type: 5-d)
GQ505726
Fusarium incarnatum-equiseti species complex (NRRL 34035; MLST type: 5-d), Fusarium incarnatum-equiseti species complex (NRRL 34035; MLST type: 5-d), 5-d, USA CO, Human sinus, n7: 28S ribosomal RNA large subunit (28S - LSU)

Sequence length: 1132

Similarity: 24/24 [100.000 %], Gaps: 0 [0.000 %], Coverage: 24/24 [100.000 %]
Score: 39.6241, Probability: 0.00194015, Direction: +/+

```
Qry 2           GCGGAGGGATCATTACCGAGTTTA  25
                ||||||||||||||||||||||||
Ref 29          GCGGAGGGATCATTACCGAGTTTA  52
```

12	Fusarium incarnatum-equiseti species complex (NRRL 36123; MLST type: 4-b) GQ505732 Fusarium incarnatum-equiseti species complex (NRRL 36123; MLST type: 4-b), Fusarium incarnatum-equiseti species complex (NRRL 36123; MLST type: 4-b), 4-b, Unknown, Unknown, n4: Internal transcribed spacers (ITS1 and ITS2)	721.158	0	100	2	96.761	+/+	****

Alignment

Reference sequence:
Fusarium incarnatum-equiseti species complex (NRRL 36123; MLST type: 4-b)
GQ505732
Fusarium incarnatum-equiseti species complex (NRRL 36123; MLST type: 4-b), Fusarium incarnatum-equiseti species complex (NRRL 36123; MLST type: 4-b), 4-b, Unknown, Unknown, n4: Internal transcribed spacers (ITS1 and ITS2)

Sequence length: 1132

Similarity: 454/454 [100.000 %], Gaps: 0 [0.000 %], Coverage: 454/454 [100.000 %]
Score: 721.158, Probability: 0, Direction: +/+

```
Qry 41          ACATACCTATACGTTGCCTCGGCGGATCAGCCCGCGCCCCGTAAAAAGGGACGGCCCGCC  100
                ||||||||||||||||||||||||||||||||||||||||||||||||||||||||||||
Ref 73          ACATACCTATACGTTGCCTCGGCGGATCAGCCCGCGCCCCGTAAAAAGGGACGGCCCGCC  132

Qry 101         CGAGGACCCCTAAACTCTGTTTTTAGTGGAACTTCTGAGTAAAACAAACAAATAAATCAA  160
                ||||||||||||||||||||||||||||||||||||||||||||||||||||||||||||
Ref 133         CGAGGACCCCTAAACTCTGTTTTTAGTGGAACTTCTGAGTAAAACAAACAAATAAATCAA  192

Qry 161         AACTTTCAACAACGGATCTCTTGGTTCTGGCATCGATGAAGAACGCAGCAAAATGCGATA  220
                ||||||||||||||||||||||||||||||||||||||||||||||||||||||||||||
```

```
Ref 193        AACTTTCAACAACGGATCTCTTGGTTCTGGCATCGATGAAGAACGCAGCAAAATGCGATA  252

Qry 221        AGTAATGTGAATTGCAGAATTCAGTGAATCATCGAATCTTTGAACGCACATTGCGCCCGC  280
               ||||||||||||||||||||||||||||||||||||||||||||||||||||||||||||
Ref 253        AGTAATGTGAATTGCAGAATTCAGTGAATCATCGAATCTTTGAACGCACATTGCGCCCGC  312

Qry 281        CAGTATTCTGGCGGGCATGCCTGTTCGAGCGTCATTTCAACCCTCAAGCTCAGCTTGGTG  340
               ||||||||||||||||||||||||||||||||||||||||||||||||||||||||||||
Ref 313        CAGTATTCTGGCGGGCATGCCTGTTCGAGCGTCATTTCAACCCTCAAGCTCAGCTTGGTG  372

Qry 341        TTGGGACTCGCGGTAACCCGCGTTCCCCAAATCGATTGGCGGTCACGTCGAGCTTCCATA  400
               ||||||||||||||||||||||||||||||||||||||||||||||||||||||||||||
Ref 373        TTGGGACTCGCGGTAACCCGCGTTCCCCAAATCGATTGGCGGTCACGTCGAGCTTCCATA  432

Qry 401        GCGTAGTAATCATACACCTCGTTACTGGTAATCGTCGCGGCCACGCCGTAAAACCCCAAC  460
               ||||||||||||||||||||||||||||||||||||||||||||||||||||||||||||
Ref 433        GCGTAGTAATCATACACCTCGTTACTGGTAATCGTCGCGGCCACGCCGTAAAACCCCAAC  492

Qry 461        TTCTGAATGTTGACCTCGGATCAGGTAGGAATAC  494
               ||||||||||||||||||||||||||||||||||
Ref 493        TTCTGAATGTTGACCTCGGATCAGGTAGGAATAC  526
```

Reference sequence:
Fusarium incarnatum-equiseti species complex (NRRL 36123; MLST type: 4-b)
GQ505732
Fusarium incarnatum-equiseti species complex (NRRL 36123; MLST type: 4-b), Fusarium incarnatum-equiseti species complex (NRRL 36123; MLST type: 4-b), 4-b, Unknown, Unknown, n4: Internal transcribed spacers (ITS1 and ITS2)

Sequence length: 1132

Similarity: 24/24 [100.000 %], Gaps: 0 [0.000 %], Coverage: 24/24 [100.000 %]
Score: 39.6241, Probability: 0.00194015, Direction: +/+

```
Qry 2          GCGGAGGGATCATTACCGAGTTTA  25
               ||||||||||||||||||||||||
Ref 29         GCGGAGGGATCATTACCGAGTTTA  52
```

13	Fusarium incarnatum-equiseti species complex (NRRL 36123; MLST type: 4-b) GQ505732 Fusarium incarnatum-equiseti species complex (NRRL 36123; MLST type: 4-b), Fusarium incarnatum-equiseti species complex (NRRL 36123; MLST type: 4-b), 4-b, Unknown, Unknown, n7: 28S ribosomal RNA large subunit (28S - LSU)	721.158	0	100	2	96.761	+/+	****

Alignment

Reference sequence:
Fusarium incarnatum-equiseti species complex (NRRL 36123; MLST type: 4-b)
GQ505732
Fusarium incarnatum-equiseti species complex (NRRL 36123; MLST type: 4-b), Fusarium incarnatum-equiseti species complex (NRRL 36123; MLST type: 4-b), 4-b, Unknown, Unknown, n7: 28S ribosomal RNA large subunit (28S - LSU)

Sequence length: 1132

Similarity: 454/454 [100.000 %], Gaps: 0 [0.000 %], Coverage: 454/454 [100.000 %]
Score: 721.158, Probability: 0, Direction: +/+

```
Qry 41         ACATACCTATACGTTGCCTCGGCGGATCAGCCCGCGCCCCGTAAAAAGGGACGGCCCGCC  100
               ||||||||||||||||||||||||||||||||||||||||||||||||||||||||||||
Ref 73         ACATACCTATACGTTGCCTCGGCGGATCAGCCCGCGCCCCGTAAAAAGGGACGGCCCGCC  132

Qry 101        CGAGGACCCCTAAACTCTGTTTTTAGTGGAACTTCTGAGTAAAACAAACAAATAAATCAA  160
               ||||||||||||||||||||||||||||||||||||||||||||||||||||||||||||
Ref 133        CGAGGACCCCTAAACTCTGTTTTTAGTGGAACTTCTGAGTAAAACAAACAAATAAATCAA  192
```

```
Qry 161       AACTTTCAACAACGGATCTCTTGGTTCTGGCATCGATGAAGAACGCAGCAAAATGCGATA  220
              ||||||||||||||||||||||||||||||||||||||||||||||||||||||||||||
Ref 193       AACTTTCAACAACGGATCTCTTGGTTCTGGCATCGATGAAGAACGCAGCAAAATGCGATA  252

Qry 221       AGTAATGTGAATTGCAGAATTCAGTGAATCATCGAATCTTTGAACGCACATTGCGCCCGC  280
              ||||||||||||||||||||||||||||||||||||||||||||||||||||||||||||
Ref 253       AGTAATGTGAATTGCAGAATTCAGTGAATCATCGAATCTTTGAACGCACATTGCGCCCGC  312

Qry 281       CAGTATTCTGGCGGGCATGCCTGTTCGAGCGTCATTTCAACCCTCAAGCTCAGCTTGGTG  340
              ||||||||||||||||||||||||||||||||||||||||||||||||||||||||||||
Ref 313       CAGTATTCTGGCGGGCATGCCTGTTCGAGCGTCATTTCAACCCTCAAGCTCAGCTTGGTG  372

Qry 341       TTGGGACTCGCGGTAACCCGCGTTCCCCAAATCGATTGGCGGTCACGTCGAGCTTCCATA  400
              ||||||||||||||||||||||||||||||||||||||||||||||||||||||||||||
Ref 373       TTGGGACTCGCGGTAACCCGCGTTCCCCAAATCGATTGGCGGTCACGTCGAGCTTCCATA  432

Qry 401       GCGTAGTAATCATACACCTCGTTACTGGTAATCGTCGCGGCCACGCCGTAAAACCCCAAC  460
              ||||||||||||||||||||||||||||||||||||||||||||||||||||||||||||
Ref 433       GCGTAGTAATCATACACCTCGTTACTGGTAATCGTCGCGGCCACGCCGTAAAACCCCAAC  492

Qry 461       TTCTGAATGTTGACCTCGGATCAGGTAGGAATAC  494
              ||||||||||||||||||||||||||||||||||
Ref 493       TTCTGAATGTTGACCTCGGATCAGGTAGGAATAC  526
```

Reference sequence:
Fusarium incarnatum-equiseti species complex (NRRL 36123; MLST type: 4-b)
GQ505732
Fusarium incarnatum-equiseti species complex (NRRL 36123; MLST type: 4-b), Fusarium incarnatum-equiseti species complex (NRRL 36123; MLST type: 4-b), 4-b, Unknown, Unknown, n7: 28S ribosomal RNA large subunit (28S - LSU)

Sequence length: 1132

Similarity: 24/24 [100.000 %], Gaps: 0 [0.000 %], Coverage: 24/24 [100.000 %]
Score: 39.6241, Probability: 0.00194015, Direction: +/+

```
Qry 2         GCGGAGGGATCATTACCGAGTTTA  25
              ||||||||||||||||||||||||
Ref 29        GCGGAGGGATCATTACCGAGTTTA  52
```

14	Fusarium incarnatum-equiseti species complex (NRRL 36318; MLST type: 3-a) GQ505735 Fusarium incarnatum-equiseti species complex (NRRL 36318; MLST type: 3-a), Fusarium incarnatum-equiseti species complex (NRRL 36318; MLST type: 3-a), 3-a, Unknown, Unknown, n4: Internal transcribed spacers (ITS1 and ITS2)	721.158	0	100	2	96.761	+/+	****

Alignment

Reference sequence:
Fusarium incarnatum-equiseti species complex (NRRL 36318; MLST type: 3-a)
GQ505735
Fusarium incarnatum-equiseti species complex (NRRL 36318; MLST type: 3-a), Fusarium incarnatum-equiseti species complex (NRRL 36318; MLST type: 3-a), 3-a, Unknown, Unknown, n4: Internal transcribed spacers (ITS1 and ITS2)

Sequence length: 1132

Similarity: 454/454 [100.000 %], Gaps: 0 [0.000 %], Coverage: 454/454 [100.000 %]
Score: 721.158, Probability: 0, Direction: +/+

```
Qry 41        ACATACCTATACGTTGCCTCGGCGGATCAGCCCGCGCCCCGTAAAAAGGGACGGCCCGCC  100
              ||||||||||||||||||||||||||||||||||||||||||||||||||||||||||||
Ref 73        ACATACCTATACGTTGCCTCGGCGGATCAGCCCGCGCCCCGTAAAAAGGGACGGCCCGCC  132
```

Qry 101 CGAGGACCCCTAAACTCTGTTTTTAGTGGAACTTCTGAGTAAAACAAACAAATAAATCAA 160
||||||||||||||||||||||||||||||||||||||||||||||||||||||||||||
Ref 133 CGAGGACCCCTAAACTCTGTTTTTAGTGGAACTTCTGAGTAAAACAAACAAATAAATCAA 192

Qry 161 AACTTTCAACAACGGATCTCTTGGTTCTGGCATCGATGAAGAACGCAGCAAAATGCGATA 220
||||||||||||||||||||||||||||||||||||||||||||||||||||||||||||
Ref 193 AACTTTCAACAACGGATCTCTTGGTTCTGGCATCGATGAAGAACGCAGCAAAATGCGATA 252

Qry 221 AGTAATGTGAATTGCAGAATTCAGTGAATCATCGAATCTTTGAACGCACATTGCGCCCGC 280
||||||||||||||||||||||||||||||||||||||||||||||||||||||||||||
Ref 253 AGTAATGTGAATTGCAGAATTCAGTGAATCATCGAATCTTTGAACGCACATTGCGCCCGC 312

Qry 281 CAGTATTCTGGCGGGCATGCCTGTTCGAGCGTCATTTCAACCCTCAAGCTCAGCTTGGTG 340
||||||||||||||||||||||||||||||||||||||||||||||||||||||||||||
Ref 313 CAGTATTCTGGCGGGCATGCCTGTTCGAGCGTCATTTCAACCCTCAAGCTCAGCTTGGTG 372

Qry 341 TTGGGACTCGCGGTAACCCGCGTTCCCCAAATCGATTGGCGGTCACGTCGAGCTTCCATA 400
||||||||||||||||||||||||||||||||||||||||||||||||||||||||||||
Ref 373 TTGGGACTCGCGGTAACCCGCGTTCCCCAAATCGATTGGCGGTCACGTCGAGCTTCCATA 432

Qry 401 GCGTAGTAATCATACACCTCGTTACTGGTAATCGTCGCGGCCACGCCGTAAAACCCCAAC 460
||||||||||||||||||||||||||||||||||||||||||||||||||||||||||||
Ref 433 GCGTAGTAATCATACACCTCGTTACTGGTAATCGTCGCGGCCACGCCGTAAAACCCCAAC 492

Qry 461 TTCTGAATGTTGACCTCGGATCAGGTAGGAATAC 494
||||||||||||||||||||||||||||||||||
Ref 493 TTCTGAATGTTGACCTCGGATCAGGTAGGAATAC 526

Reference sequence:
Fusarium incarnatum-equiseti species complex (NRRL 36318; MLST type: 3-a)
GQ505735
Fusarium incarnatum-equiseti species complex (NRRL 36318; MLST type: 3-a), Fusarium incarnatum-equiseti species complex (NRRL 36318; MLST type: 3-a), 3-a, Unknown, Unknown, n4: Internal transcribed spacers (ITS1 and ITS2)

Sequence length: 1132

Similarity: 24/24 [100.000 %], Gaps: 0 [0.000 %], Coverage: 24/24 [100.000 %]
Score: 39.6241, Probability: 0.00194015, Direction: +/+

Qry 2 GCGGAGGGATCATTACCGAGTTTA 25
||||||||||||||||||||||||
Ref 29 GCGGAGGGATCATTACCGAGTTTA 52

15	Fusarium incarnatum-equiseti species complex (NRRL 36318; MLST type: 3-a) GQ505735 Fusarium incarnatum-equiseti species complex (NRRL 36318; MLST type: 3-a), Fusarium incarnatum-equiseti species complex (NRRL 36318; MLST type: 3-a), 3-a, Unknown, Unknown, n7: 28S ribosomal RNA large subunit (28S - LSU)	721.158	0	100	2	96.761	+/+	****

Alignment

Reference sequence:
Fusarium incarnatum-equiseti species complex (NRRL 36318; MLST type: 3-a)
GQ505735
Fusarium incarnatum-equiseti species complex (NRRL 36318; MLST type: 3-a), Fusarium incarnatum-equiseti species complex (NRRL 36318; MLST type: 3-a), 3-a, Unknown, Unknown, n7: 28S ribosomal RNA large subunit (28S - LSU)

Sequence length: 1132

Similarity: 454/454 [100.000 %], Gaps: 0 [0.000 %], Coverage: 454/454 [100.000 %]
Score: 721.158, Probability: 0, Direction: +/+

Qry 41 ACATACCTATACGTTGCCTCGGCGGATCAGCCCGCGCCCCGTAAAAAGGGACGGCCCGCC 100

```
              ||||||||||||||||||||||||||||||||||||||||||||||||||||||||||||
Ref 73        ACATACCTATACGTTGCCTCGGCGGATCAGCCCGCGCCCCGTAAAAAGGGACGGCCCGCC  132

Qry 101       CGAGGACCCCTAAACTCTGTTTTTAGTGGAACTTCTGAGTAAAACAAACAAATAAATCAA  160
              ||||||||||||||||||||||||||||||||||||||||||||||||||||||||||||
Ref 133       CGAGGACCCCTAAACTCTGTTTTTAGTGGAACTTCTGAGTAAAACAAACAAATAAATCAA  192

Qry 161       AACTTTCAACAACGGATCTCTTGGTTCTGGCATCGATGAAGAACGCAGCAAAATGCGATA  220
              ||||||||||||||||||||||||||||||||||||||||||||||||||||||||||||
Ref 193       AACTTTCAACAACGGATCTCTTGGTTCTGGCATCGATGAAGAACGCAGCAAAATGCGATA  252

Qry 221       AGTAATGTGAATTGCAGAATTCAGTGAATCATCGAATCTTTGAACGCACATTGCGCCCGC  280
              ||||||||||||||||||||||||||||||||||||||||||||||||||||||||||||
Ref 253       AGTAATGTGAATTGCAGAATTCAGTGAATCATCGAATCTTTGAACGCACATTGCGCCCGC  312

Qry 281       CAGTATTCTGGCGGGCATGCCTGTTCGAGCGTCATTTCAACCCTCAAGCTCAGCTTGGTG  340
              ||||||||||||||||||||||||||||||||||||||||||||||||||||||||||||
Ref 313       CAGTATTCTGGCGGGCATGCCTGTTCGAGCGTCATTTCAACCCTCAAGCTCAGCTTGGTG  372

Qry 341       TTGGGACTCGCGGTAACCCGCGTTCCCCAAATCGATTGGCGGTCACGTCGAGCTTCCATA  400
              ||||||||||||||||||||||||||||||||||||||||||||||||||||||||||||
Ref 373       TTGGGACTCGCGGTAACCCGCGTTCCCCAAATCGATTGGCGGTCACGTCGAGCTTCCATA  432

Qry 401       GCGTAGTAATCATACACCTCGTTACTGGTAATCGTCGCGGCCACGCCGTAAAACCCCAAC  460
              ||||||||||||||||||||||||||||||||||||||||||||||||||||||||||||
Ref 433       GCGTAGTAATCATACACCTCGTTACTGGTAATCGTCGCGGCCACGCCGTAAAACCCCAAC  492

Qry 461       TTCTGAATGTTGACCTCGGATCAGGTAGGAATAC  494
              ||||||||||||||||||||||||||||||||||
Ref 493       TTCTGAATGTTGACCTCGGATCAGGTAGGAATAC  526
```

Reference sequence:
Fusarium incarnatum-equiseti species complex (NRRL 36318; MLST type: 3-a)
GQ505735
Fusarium incarnatum-equiseti species complex (NRRL 36318; MLST type: 3-a), Fusarium incarnatum-equiseti species complex (NRRL 36318; MLST type: 3-a), 3-a, Unknown, Unknown, n7: 28S ribosomal RNA large subunit (28S - LSU)

Sequence length: 1132

Similarity: 24/24 [100.000 %], Gaps: 0 [0.000 %], Coverage: 24/24 [100.000 %]
Score: 39.6241, Probability: 0.00194015, Direction: +/+

```
Qry 2         GCGGAGGGATCATTACCGAGTTTA  25
              ||||||||||||||||||||||||
Ref 29        GCGGAGGGATCATTACCGAGTTTA  52
```

16	Fusarium incarnatum-equiseti species complex (NRRL 36323; MLST type: 3-a) GQ505737 Fusarium incarnatum-equiseti species complex (NRRL 36323; MLST type: 3-a), Fusarium incarnatum-equiseti species complex (NRRL 36323; MLST type: 3-a), 3-a, England, Cotton yarn, n4: Internal transcribed spacers (ITS1 and ITS2)	721.158	0	100	2	96.761	+/+	****

Alignment

Reference sequence:
Fusarium incarnatum-equiseti species complex (NRRL 36323; MLST type: 3-a)
GQ505737
Fusarium incarnatum-equiseti species complex (NRRL 36323; MLST type: 3-a), Fusarium incarnatum-equiseti species complex (NRRL 36323; MLST type: 3-a), 3-a, England, Cotton yarn, n4: Internal transcribed spacers (ITS1 and ITS2)

Sequence length: 1132

Similarity: 454/454 [100.000 %], Gaps: 0 [0.000 %], Coverage: 454/454 [100.000 %]

Score: 721.158, Probability: 0, Direction: +/+

```
Qry 41         ACATACCTATACGTTGCCTCGGCGGATCAGCCCGCGCCCCGTAAAAAGGGACGGCCCGCC  100
               ||||||||||||||||||||||||||||||||||||||||||||||||||||||||||||
Ref 73         ACATACCTATACGTTGCCTCGGCGGATCAGCCCGCGCCCCGTAAAAAGGGACGGCCCGCC  132

Qry 101        CGAGGACCCCTAAACTCTGTTTTTAGTGGAACTTCTGAGTAAAACAAACAAATAAATCAA  160
               ||||||||||||||||||||||||||||||||||||||||||||||||||||||||||||
Ref 133        CGAGGACCCCTAAACTCTGTTTTTAGTGGAACTTCTGAGTAAAACAAACAAATAAATCAA  192

Qry 161        AACTTTCAACAACGGATCTCTTGGTTCTGGCATCGATGAAGAACGCAGCAAAATGCGATA  220
               ||||||||||||||||||||||||||||||||||||||||||||||||||||||||||||
Ref 193        AACTTTCAACAACGGATCTCTTGGTTCTGGCATCGATGAAGAACGCAGCAAAATGCGATA  252

Qry 221        AGTAATGTGAATTGCAGAATTCAGTGAATCATCGAATCTTTGAACGCACATTGCGCCCGC  280
               ||||||||||||||||||||||||||||||||||||||||||||||||||||||||||||
Ref 253        AGTAATGTGAATTGCAGAATTCAGTGAATCATCGAATCTTTGAACGCACATTGCGCCCGC  312

Qry 281        CAGTATTCTGGCGGGCATGCCTGTTCGAGCGTCATTTCAACCCTCAAGCTCAGCTTGGTG  340
               ||||||||||||||||||||||||||||||||||||||||||||||||||||||||||||
Ref 313        CAGTATTCTGGCGGGCATGCCTGTTCGAGCGTCATTTCAACCCTCAAGCTCAGCTTGGTG  372

Qry 341        TTGGGACTCGCGGTAACCCGCGTTCCCCAAATCGATTGGCGGTCACGTCGAGCTTCCATA  400
               ||||||||||||||||||||||||||||||||||||||||||||||||||||||||||||
Ref 373        TTGGGACTCGCGGTAACCCGCGTTCCCCAAATCGATTGGCGGTCACGTCGAGCTTCCATA  432

Qry 401        GCGTAGTAATCATACACCTCGTTACTGGTAATCGTCGCGGCCACGCCGTAAAACCCCAAC  460
               ||||||||||||||||||||||||||||||||||||||||||||||||||||||||||||
Ref 433        GCGTAGTAATCATACACCTCGTTACTGGTAATCGTCGCGGCCACGCCGTAAAACCCCAAC  492

Qry 461        TTCTGAATGTTGACCTCGGATCAGGTAGGAATAC  494
               ||||||||||||||||||||||||||||||||||
Ref 493        TTCTGAATGTTGACCTCGGATCAGGTAGGAATAC  526
```

Reference sequence:
Fusarium incarnatum-equiseti species complex (NRRL 36323; MLST type: 3-a)
GQ505737
Fusarium incarnatum-equiseti species complex (NRRL 36323; MLST type: 3-a), Fusarium incarnatum-equiseti species complex (NRRL 36323; MLST type: 3-a), 3-a, England, Cotton yarn, n4: Internal transcribed spacers (ITS1 and ITS2)

Sequence length: 1132

Similarity: 24/24 [100.000 %], Gaps: 0 [0.000 %], Coverage: 24/24 [100.000 %]
Score: 39.6241, Probability: 0.00194015, Direction: +/+

```
Qry 2          GCGGAGGGATCATTACCGAGTTTA  25
               ||||||||||||||||||||||||
Ref 29         GCGGAGGGATCATTACCGAGTTTA  52
```

17	Fusarium incarnatum-equiseti species complex (NRRL 36323; MLST type: 3-a) GQ505737 Fusarium incarnatum-equiseti species complex (NRRL 36323; MLST type: 3-a), Fusarium incarnatum-equiseti species complex (NRRL 36323; MLST type: 3-a), 3-a, England, Cotton yarn, n7: 28S ribosomal RNA large subunit (28S - LSU)	721.158	0	100	2	96.761	+/+	****

Alignment

Reference sequence:
Fusarium incarnatum-equiseti species complex (NRRL 36323; MLST type: 3-a)
GQ505737
Fusarium incarnatum-equiseti species complex (NRRL 36323; MLST type: 3-a), Fusarium incarnatum-equiseti species complex (NRRL 36323; MLST type: 3-a), 3-a, England, Cotton yarn, n7: 28S ribosomal RNA large subunit (28S - LSU)

Sequence length: 1132

Similarity: 454/454 [100.000 %], Gaps: 0 [0.000 %], Coverage: 454/454 [100.000 %]
Score: 721.158, Probability: 0, Direction: +/+

```
Qry 41        ACATACCTATACGTTGCCTCGGCGGATCAGCCCGCGCCCCGTAAAAAGGGACGGCCCGCC  100
              ||||||||||||||||||||||||||||||||||||||||||||||||||||||||||||
Ref 73        ACATACCTATACGTTGCCTCGGCGGATCAGCCCGCGCCCCGTAAAAAGGGACGGCCCGCC  132

Qry 101       CGAGGACCCCTAAACTCTGTTTTTAGTGGAACTTCTGAGTAAAACAAACAAATAAATCAA  160
              ||||||||||||||||||||||||||||||||||||||||||||||||||||||||||||
Ref 133       CGAGGACCCCTAAACTCTGTTTTTAGTGGAACTTCTGAGTAAAACAAACAAATAAATCAA  192

Qry 161       AACTTTCAACAACGGATCTCTTGGTTCTGGCATCGATGAAGAACGCAGCAAAATGCGATA  220
              ||||||||||||||||||||||||||||||||||||||||||||||||||||||||||||
Ref 193       AACTTTCAACAACGGATCTCTTGGTTCTGGCATCGATGAAGAACGCAGCAAAATGCGATA  252

Qry 221       AGTAATGTGAATTGCAGAATTCAGTGAATCATCGAATCTTTGAACGCACATTGCGCCCGC  280
              ||||||||||||||||||||||||||||||||||||||||||||||||||||||||||||
Ref 253       AGTAATGTGAATTGCAGAATTCAGTGAATCATCGAATCTTTGAACGCACATTGCGCCCGC  312

Qry 281       CAGTATTCTGGCGGGCATGCCTGTTCGAGCGTCATTTCAACCCTCAAGCTCAGCTTGGTG  340
              ||||||||||||||||||||||||||||||||||||||||||||||||||||||||||||
Ref 313       CAGTATTCTGGCGGGCATGCCTGTTCGAGCGTCATTTCAACCCTCAAGCTCAGCTTGGTG  372

Qry 341       TTGGGACTCGCGGTAACCCGCGTTCCCCAAATCGATTGGCGGTCACGTCGAGCTTCCATA  400
              ||||||||||||||||||||||||||||||||||||||||||||||||||||||||||||
Ref 373       TTGGGACTCGCGGTAACCCGCGTTCCCCAAATCGATTGGCGGTCACGTCGAGCTTCCATA  432

Qry 401       GCGTAGTAATCATACACCTCGTTACTGGTAATCGTCGCGGCCACGCCGTAAAACCCCAAC  460
              ||||||||||||||||||||||||||||||||||||||||||||||||||||||||||||
Ref 433       GCGTAGTAATCATACACCTCGTTACTGGTAATCGTCGCGGCCACGCCGTAAAACCCCAAC  492

Qry 461       TTCTGAATGTTGACCTCGGATCAGGTAGGAATAC  494
              ||||||||||||||||||||||||||||||||||
Ref 493       TTCTGAATGTTGACCTCGGATCAGGTAGGAATAC  526
```

Reference sequence:
Fusarium incarnatum-equiseti species complex (NRRL 36323; MLST type: 3-a)
GQ505737
Fusarium incarnatum-equiseti species complex (NRRL 36323; MLST type: 3-a), Fusarium incarnatum-equiseti species complex (NRRL 36323; MLST type: 3-a), 3-a, England, Cotton yarn, n7: 28S ribosomal RNA large subunit (28S - LSU)

Sequence length: 1132

Similarity: 24/24 [100.000 %], Gaps: 0 [0.000 %], Coverage: 24/24 [100.000 %]
Score: 39.6241, Probability: 0.00194015, Direction: +/+

```
Qry 2         GCGGAGGGATCATTACCGAGTTTA  25
              ||||||||||||||||||||||||
Ref 29        GCGGAGGGATCATTACCGAGTTTA  52
```

18	Fusarium incarnatum-equiseti species complex (NRRL 36448; MLST type: 2-b) GQ505741 Fusarium incarnatum-equiseti species complex (NRRL 36448; MLST type: 2-b), Fusarium incarnatum-equiseti species complex (NRRL 36448; MLST type: 2-b), 2-b, Sudan, Bean (Phaseolus vulgaris) seed, n4: Internal transcribed spacers (ITS1 and ITS2)	721.158	0	100	2	96.761	+/+	****

Alignment

Reference sequence:
Fusarium incarnatum-equiseti species complex (NRRL 36448; MLST type: 2-b)
GQ505741

Fusarium incarnatum-equiseti species complex (NRRL 36448; MLST type: 2-b), Fusarium incarnatum-equiseti species complex (NRRL 36448; MLST type: 2-b), 2-b, Sudan, Bean (Phaseolus vulgaris) seed, n4: Internal transcribed spacers (ITS1 and ITS2)

Sequence length: 1132

Similarity: 454/454 [100.000 %], Gaps: 0 [0.000 %], Coverage: 454/454 [100.000 %]
Score: 721.158, Probability: 0, Direction: +/+

```
Qry 41         ACATACCTATACGTTGCCTCGGCGGATCAGCCCGCGCCCCGTAAAAAGGGACGGCCCGCC  100
               ||||||||||||||||||||||||||||||||||||||||||||||||||||||||||||
Ref 73         ACATACCTATACGTTGCCTCGGCGGATCAGCCCGCGCCCCGTAAAAAGGGACGGCCCGCC  132

Qry 101        CGAGGACCCCTAAACTCTGTTTTTAGTGGAACTTCTGAGTAAAACAAACAAATAAATCAA  160
               ||||||||||||||||||||||||||||||||||||||||||||||||||||||||||||
Ref 133        CGAGGACCCCTAAACTCTGTTTTTAGTGGAACTTCTGAGTAAAACAAACAAATAAATCAA  192

Qry 161        AACTTTCAACAACGGATCTCTTGGTTCTGGCATCGATGAAGAACGCAGCAAAATGCGATA  220
               ||||||||||||||||||||||||||||||||||||||||||||||||||||||||||||
Ref 193        AACTTTCAACAACGGATCTCTTGGTTCTGGCATCGATGAAGAACGCAGCAAAATGCGATA  252

Qry 221        AGTAATGTGAATTGCAGAATTCAGTGAATCATCGAATCTTTGAACGCACATTGCGCCCGC  280
               ||||||||||||||||||||||||||||||||||||||||||||||||||||||||||||
Ref 253        AGTAATGTGAATTGCAGAATTCAGTGAATCATCGAATCTTTGAACGCACATTGCGCCCGC  312

Qry 281        CAGTATTCTGGCGGGCATGCCTGTTCGAGCGTCATTTCAACCCTCAAGCTCAGCTTGGTG  340
               ||||||||||||||||||||||||||||||||||||||||||||||||||||||||||||
Ref 313        CAGTATTCTGGCGGGCATGCCTGTTCGAGCGTCATTTCAACCCTCAAGCTCAGCTTGGTG  372

Qry 341        TTGGGACTCGCGGTAACCCGCGTTCCCCAAATCGATTGGCGGTCACGTCGAGCTTCCATA  400
               ||||||||||||||||||||||||||||||||||||||||||||||||||||||||||||
Ref 373        TTGGGACTCGCGGTAACCCGCGTTCCCCAAATCGATTGGCGGTCACGTCGAGCTTCCATA  432

Qry 401        GCGTAGTAATCATACACCTCGTTACTGGTAATCGTCGCGGCCACGCCGTAAAACCCCAAC  460
               ||||||||||||||||||||||||||||||||||||||||||||||||||||||||||||
Ref 433        GCGTAGTAATCATACACCTCGTTACTGGTAATCGTCGCGGCCACGCCGTAAAACCCCAAC  492

Qry 461        TTCTGAATGTTGACCTCGGATCAGGTAGGAATAC  494
               ||||||||||||||||||||||||||||||||||
Ref 493        TTCTGAATGTTGACCTCGGATCAGGTAGGAATAC  526
```

Reference sequence:
Fusarium incarnatum-equiseti species complex (NRRL 36448; MLST type: 2-b)
GQ505741
Fusarium incarnatum-equiseti species complex (NRRL 36448; MLST type: 2-b), Fusarium incarnatum-equiseti species complex (NRRL 36448; MLST type: 2-b), 2-b, Sudan, Bean (Phaseolus vulgaris) seed, n4: Internal transcribed spacers (ITS1 and ITS2)

Sequence length: 1132
Similarity: 24/24 [100.000 %], Gaps: 0 [0.000 %], Coverage: 24/24 [100.000 %]
Score: 39.6241, Probability: 0.00194015, Direction: +/+

```
Qry 2          GCGGAGGGATCATTACCGAGTTTA  25
               ||||||||||||||||||||||||
Ref 29         GCGGAGGGATCATTACCGAGTTTA  52
```

19	Fusarium incarnatum-equiseti species complex (NRRL 20423; MLST type: 4-a) GQ505682 Fusarium incarnatum-equiseti species complex (NRRL 20423; MLST type: 4-a), Fusarium incarnatum-equiseti species complex (NRRL 20423; MLST type: 4-a), 4-a, India, Lizard skin, n4: Internal transcribed spacers (ITS1 and ITS2)	721.158	0	100	2	96.761	+/+	****

Alignment

Reference sequence:
Fusarium incarnatum-equiseti species complex (NRRL 20423; MLST type: 4-a)
GQ505682
Fusarium incarnatum-equiseti species complex (NRRL 20423; MLST type: 4-a), Fusarium incarnatum-equiseti species complex (NRRL 20423; MLST type: 4-a), 4-a, India, Lizard skin, n4: Internal transcribed spacers (ITS1 and ITS2)

Sequence length: 1132

Similarity: 454/454 [100.000 %], Gaps: 0 [0.000 %], Coverage: 454/454 [100.000 %]
Score: 721.158, Probability: 0, Direction: +/+

```
Qry 41         ACATACCTATACGTTGCCTCGGCGGATCAGCCCGCGCCCCGTAAAAAGGGACGGCCCGCC  100
               ||||||||||||||||||||||||||||||||||||||||||||||||||||||||||||
Ref 73         ACATACCTATACGTTGCCTCGGCGGATCAGCCCGCGCCCCGTAAAAAGGGACGGCCCGCC  132

Qry 101        CGAGGACCCCTAAACTCTGTTTTTAGTGGAACTTCTGAGTAAAACAAACAAATAAATCAA  160
               ||||||||||||||||||||||||||||||||||||||||||||||||||||||||||||
Ref 133        CGAGGACCCCTAAACTCTGTTTTTAGTGGAACTTCTGAGTAAAACAAACAAATAAATCAA  192

Qry 161        AACTTTCAACAACGGATCTCTTGGTTCTGGCATCGATGAAGAACGCAGCAAAATGCGATA  220
               ||||||||||||||||||||||||||||||||||||||||||||||||||||||||||||
Ref 193        AACTTTCAACAACGGATCTCTTGGTTCTGGCATCGATGAAGAACGCAGCAAAATGCGATA  252

Qry 221        AGTAATGTGAATTGCAGAATTCAGTGAATCATCGAATCTTTGAACGCACATTGCGCCCGC  280
               ||||||||||||||||||||||||||||||||||||||||||||||||||||||||||||
Ref 253        AGTAATGTGAATTGCAGAATTCAGTGAATCATCGAATCTTTGAACGCACATTGCGCCCGC  312

Qry 281        CAGTATTCTGGCGGGCATGCCTGTTCGAGCGTCATTTCAACCCTCAAGCTCAGCTTGGTG  340
               ||||||||||||||||||||||||||||||||||||||||||||||||||||||||||||
Ref 313        CAGTATTCTGGCGGGCATGCCTGTTCGAGCGTCATTTCAACCCTCAAGCTCAGCTTGGTG  372

Qry 341        TTGGGACTCGCGGTAACCCGCGTTCCCCAAATCGATTGGCGGTCACGTCGAGCTTCCATA  400
               ||||||||||||||||||||||||||||||||||||||||||||||||||||||||||||
Ref 373        TTGGGACTCGCGGTAACCCGCGTTCCCCAAATCGATTGGCGGTCACGTCGAGCTTCCATA  432

Qry 401        GCGTAGTAATCATACACCTCGTTACTGGTAATCGTCGCGGCCACGCCGTAAAACCCCAAC  460
               ||||||||||||||||||||||||||||||||||||||||||||||||||||||||||||
Ref 433        GCGTAGTAATCATACACCTCGTTACTGGTAATCGTCGCGGCCACGCCGTAAAACCCCAAC  492

Qry 461        TTCTGAATGTTGACCTCGGATCAGGTAGGAATAC  494
               ||||||||||||||||||||||||||||||||||
Ref 493        TTCTGAATGTTGACCTCGGATCAGGTAGGAATAC  526
```

Reference sequence:
Fusarium incarnatum-equiseti species complex (NRRL 20423; MLST type: 4-a)
GQ505682
Fusarium incarnatum-equiseti species complex (NRRL 20423; MLST type: 4-a), Fusarium incarnatum-equiseti species complex (NRRL 20423; MLST type: 4-a), 4-a, India, Lizard skin, n4: Internal transcribed spacers (ITS1 and ITS2)

Sequence length: 1132

Similarity: 24/24 [100.000 %], Gaps: 0 [0.000 %], Coverage: 24/24 [100.000 %]
Score: 39.6241, Probability: 0.00194015, Direction: +/+

```
Qry 2          GCGGAGGGATCATTACCGAGTTTA  25
               ||||||||||||||||||||||||
Ref 29         GCGGAGGGATCATTACCGAGTTTA  52
```

20	Fusarium incarnatum-equiseti species complex (NRRL 45997; MLST type: 5-f) GQ505761 Fusarium incarnatum-equiseti species complex (NRRL 45997; MLST type: 5-f), Fusarium incarnatum-equiseti species complex (NRRL 45997; MLST type: 5-f), 5-f, USA CO, Human sinus, n7: 28S ribosomal RNA	721.158	0	100	2	96.761	+/+	****

large subunit (28S -
LSU)

Alignment

Reference sequence:
Fusarium incarnatum-equiseti species complex (NRRL 45997; MLST type: 5-f)
GQ505761
Fusarium incarnatum-equiseti species complex (NRRL 45997; MLST type: 5-f), Fusarium incarnatum-equiseti species complex (NRRL 45997; MLST type: 5-f), 5-f, USA CO, Human sinus, n7: 28S ribosomal RNA large subunit (28S - LSU)

Sequence length: 1132

Similarity: 454/454 [100.000 %], Gaps: 0 [0.000 %], Coverage: 454/454 [100.000 %]
Score: 721.158, Probability: 0, Direction: +/+

```
Qry 41         ACATACCTATACGTTGCCTCGGCGGATCAGCCCGCGCCCCGTAAAAAGGGACGGCCCGCC  100
               ||||||||||||||||||||||||||||||||||||||||||||||||||||||||||||
Ref 73         ACATACCTATACGTTGCCTCGGCGGATCAGCCCGCGCCCCGTAAAAAGGGACGGCCCGCC  132

Qry 101        CGAGGACCCCTAAACTCTGTTTTTAGTGGAACTTCTGAGTAAAACAAACAAATAAATCAA  160
               ||||||||||||||||||||||||||||||||||||||||||||||||||||||||||||
Ref 133        CGAGGACCCCTAAACTCTGTTTTTAGTGGAACTTCTGAGTAAAACAAACAAATAAATCAA  192

Qry 161        AACTTTCAACAACGGATCTCTTGGTTCTGGCATCGATGAAGAACGCAGCAAAATGCGATA  220
               ||||||||||||||||||||||||||||||||||||||||||||||||||||||||||||
Ref 193        AACTTTCAACAACGGATCTCTTGGTTCTGGCATCGATGAAGAACGCAGCAAAATGCGATA  252

Qry 221        AGTAATGTGAATTGCAGAATTCAGTGAATCATCGAATCTTTGAACGCACATTGCGCCCGC  280
               ||||||||||||||||||||||||||||||||||||||||||||||||||||||||||||
Ref 253        AGTAATGTGAATTGCAGAATTCAGTGAATCATCGAATCTTTGAACGCACATTGCGCCCGC  312

Qry 281        CAGTATTCTGGCGGGCATGCCTGTTCGAGCGTCATTTCAACCCTCAAGCTCAGCTTGGTG  340
               ||||||||||||||||||||||||||||||||||||||||||||||||||||||||||||
Ref 313        CAGTATTCTGGCGGGCATGCCTGTTCGAGCGTCATTTCAACCCTCAAGCTCAGCTTGGTG  372

Qry 341        TTGGGACTCGCGGTAACCCGCGTTCCCCAAATCGATTGGCGGTCACGTCGAGCTTCCATA  400
               ||||||||||||||||||||||||||||||||||||||||||||||||||||||||||||
Ref 373        TTGGGACTCGCGGTAACCCGCGTTCCCCAAATCGATTGGCGGTCACGTCGAGCTTCCATA  432

Qry 401        GCGTAGTAATCATACACCTCGTTACTGGTAATCGTCGCGGCCACGCCGTAAAACCCCAAC  460
               ||||||||||||||||||||||||||||||||||||||||||||||||||||||||||||
Ref 433        GCGTAGTAATCATACACCTCGTTACTGGTAATCGTCGCGGCCACGCCGTAAAACCCCAAC  492

Qry 461        TTCTGAATGTTGACCTCGGATCAGGTAGGAATAC  494
               ||||||||||||||||||||||||||||||||||
Ref 493        TTCTGAATGTTGACCTCGGATCAGGTAGGAATAC  526
```

Reference sequence:
Fusarium incarnatum-equiseti species complex (NRRL 45997; MLST type: 5-f)
GQ505761
Fusarium incarnatum-equiseti species complex (NRRL 45997; MLST type: 5-f), Fusarium incarnatum-equiseti species complex (NRRL 45997; MLST type: 5-f), 5-f, USA CO, Human sinus, n7: 28S ribosomal RNA large subunit (28S - LSU)

Sequence length: 1132

Similarity: 24/24 [100.000 %], Gaps: 0 [0.000 %], Coverage: 24/24 [100.000 %]
Score: 39.6241, Probability: 0.00194015, Direction: +/+

```
Qry 2          GCGGAGGGATCATTACCGAGTTTA  25
               ||||||||||||||||||||||||
Ref 29         GCGGAGGGATCATTACCGAGTTTA  52
```

21	Fusarium incarnatum-equiseti species complex (NRRL 45997; MLST type: 5-f) GQ505761 Fusarium incarnatum-equiseti species complex (NRRL 45997; MLST type: 5-f), Fusarium incarnatum-equiseti species complex (NRRL 45997; MLST	721.158	0	100	2	96.761	+/+	****

type: 5-f), 5-f, USA CO, Human sinus, n4: Internal transcribed spacers (ITS1 and ITS2)

Alignment

Reference sequence:
[Fusarium incarnatum-equiseti species complex (NRRL 45997; MLST type: 5-f) GQ505761]
Fusarium incarnatum-equiseti species complex (NRRL 45997; MLST type: 5-f), Fusarium incarnatum-equiseti species complex (NRRL 45997; MLST type: 5-f), 5-f, USA CO, Human sinus, n4: Internal transcribed spacers (ITS1 and ITS2)

Sequence length: 1132

Similarity: 454/454 [100.000 %], Gaps: 0 [0.000 %], Coverage: 454/454 [100.000 %]
Score: 721.158, Probability: 0, Direction: +/+

```
Qry 41         ACATACCTATACGTTGCCTCGGCGGATCAGCCCGCGCCCCGTAAAAAGGGACGGCCCGCC  100
               ||||||||||||||||||||||||||||||||||||||||||||||||||||||||||||
Ref 73         ACATACCTATACGTTGCCTCGGCGGATCAGCCCGCGCCCCGTAAAAAGGGACGGCCCGCC  132

Qry 101        CGAGGACCCCTAAACTCTGTTTTTAGTGGAACTTCTGAGTAAAACAAACAAATAAATCAA  160
               ||||||||||||||||||||||||||||||||||||||||||||||||||||||||||||
Ref 133        CGAGGACCCCTAAACTCTGTTTTTAGTGGAACTTCTGAGTAAAACAAACAAATAAATCAA  192

Qry 161        AACTTTCAACAACGGATCTCTTGGTTCTGGCATCGATGAAGAACGCAGCAAAATGCGATA  220
               ||||||||||||||||||||||||||||||||||||||||||||||||||||||||||||
Ref 193        AACTTTCAACAACGGATCTCTTGGTTCTGGCATCGATGAAGAACGCAGCAAAATGCGATA  252

Qry 221        AGTAATGTGAATTGCAGAATTCAGTGAATCATCGAATCTTTGAACGCACATTGCGCCCGC  280
               ||||||||||||||||||||||||||||||||||||||||||||||||||||||||||||
Ref 253        AGTAATGTGAATTGCAGAATTCAGTGAATCATCGAATCTTTGAACGCACATTGCGCCCGC  312

Qry 281        CAGTATTCTGGCGGGCATGCCTGTTCGAGCGTCATTTCAACCCTCAAGCTCAGCTTGGTG  340
               ||||||||||||||||||||||||||||||||||||||||||||||||||||||||||||
Ref 313        CAGTATTCTGGCGGGCATGCCTGTTCGAGCGTCATTTCAACCCTCAAGCTCAGCTTGGTG  372

Qry 341        TTGGGACTCGCGGTAACCCGCGTTCCCCAAATCGATTGGCGGTCACGTCGAGCTTCCATA  400
               ||||||||||||||||||||||||||||||||||||||||||||||||||||||||||||
Ref 373        TTGGGACTCGCGGTAACCCGCGTTCCCCAAATCGATTGGCGGTCACGTCGAGCTTCCATA  432

Qry 401        GCGTAGTAATCATACACCTCGTTACTGGTAATCGTCGCGGCCACGCCGTAAAACCCCAAC  460
               ||||||||||||||||||||||||||||||||||||||||||||||||||||||||||||
Ref 433        GCGTAGTAATCATACACCTCGTTACTGGTAATCGTCGCGGCCACGCCGTAAAACCCCAAC  492

Qry 461        TTCTGAATGTTGACCTCGGATCAGGTAGGAATAC  494
               ||||||||||||||||||||||||||||||||||
Ref 493        TTCTGAATGTTGACCTCGGATCAGGTAGGAATAC  526
```

Reference sequence:
[Fusarium incarnatum-equiseti species complex (NRRL 45997; MLST type: 5-f) GQ505761]
Fusarium incarnatum-equiseti species complex (NRRL 45997; MLST type: 5-f), Fusarium incarnatum-equiseti species complex (NRRL 45997; MLST type: 5-f), 5-f, USA CO, Human sinus, n4: Internal transcribed spacers (ITS1 and ITS2)

Sequence length: 1132

Similarity: 24/24 [100.000 %], Gaps: 0 [0.000 %], Coverage: 24/24 [100.000 %]
Score: 39.6241, Probability: 0.00194015, Direction: +/+

```
Qry 2          GCGGAGGGATCATTACCGAGTTTA  25
               ||||||||||||||||||||||||
Ref 29         GCGGAGGGATCATTACCGAGTTTA  52
```

22	Fusarium incarnatum-equiseti species complex (NRRL 43640; MLST type: 1-a) GQ505756 Fusarium incarnatum-equiseti species complex (NRRL 43640; MLST type: 1-a), Fusarium	721.158	0	100	2	96.761	+/+	****

incarnatum-equiseti
species complex
(NRRL 43640; MLST
type: 1-a), 1-a, USA
TX, Dog nose, n7:
28S ribosomal RNA
large subunit (28S -
LSU)

Alignment

Reference sequence:
Fusarium incarnatum-equiseti species complex (NRRL 43640; MLST type: 1-a)
GQ505756
Fusarium incarnatum-equiseti species complex (NRRL 43640; MLST type: 1-a), Fusarium incarnatum-equiseti species complex (NRRL 43640; MLST type: 1-a), 1-a, USA TX, Dog nose, n7: 28S ribosomal RNA large subunit (28S - LSU)

Sequence length: 1132

Similarity: 454/454 [100.000 %], Gaps: 0 [0.000 %], Coverage: 454/454 [100.000 %]
Score: 721.158, Probability: 0, Direction: +/+

```
Qry 41         ACATACCTATACGTTGCCTCGGCGGATCAGCCCGCGCCCCGTAAAAAGGGACGGCCCGCC  100
               ||||||||||||||||||||||||||||||||||||||||||||||||||||||||||||
Ref 73         ACATACCTATACGTTGCCTCGGCGGATCAGCCCGCGCCCCGTAAAAAGGGACGGCCCGCC  132

Qry 101        CGAGGACCCCTAAACTCTGTTTTTAGTGGAACTTCTGAGTAAAACAAACAAATAAATCAA  160
               ||||||||||||||||||||||||||||||||||||||||||||||||||||||||||||
Ref 133        CGAGGACCCCTAAACTCTGTTTTTAGTGGAACTTCTGAGTAAAACAAACAAATAAATCAA  192

Qry 161        AACTTTCAACAACGGATCTCTTGGTTCTGGCATCGATGAAGAACGCAGCAAAATGCGATA  220
               ||||||||||||||||||||||||||||||||||||||||||||||||||||||||||||
Ref 193        AACTTTCAACAACGGATCTCTTGGTTCTGGCATCGATGAAGAACGCAGCAAAATGCGATA  252

Qry 221        AGTAATGTGAATTGCAGAATTCAGTGAATCATCGAATCTTTGAACGCACATTGCGCCCGC  280
               ||||||||||||||||||||||||||||||||||||||||||||||||||||||||||||
Ref 253        AGTAATGTGAATTGCAGAATTCAGTGAATCATCGAATCTTTGAACGCACATTGCGCCCGC  312

Qry 281        CAGTATTCTGGCGGGCATGCCTGTTCGAGCGTCATTTCAACCCTCAAGCTCAGCTTGGTG  340
               ||||||||||||||||||||||||||||||||||||||||||||||||||||||||||||
Ref 313        CAGTATTCTGGCGGGCATGCCTGTTCGAGCGTCATTTCAACCCTCAAGCTCAGCTTGGTG  372

Qry 341        TTGGGACTCGCGGTAACCCGCGTTCCCCAAATCGATTGGCGGTCACGTCGAGCTTCCATA  400
               ||||||||||||||||||||||||||||||||||||||||||||||||||||||||||||
Ref 373        TTGGGACTCGCGGTAACCCGCGTTCCCCAAATCGATTGGCGGTCACGTCGAGCTTCCATA  432

Qry 401        GCGTAGTAATCATACACCTCGTTACTGGTAATCGTCGCGGCCACGCCGTAAAACCCCAAC  460
               ||||||||||||||||||||||||||||||||||||||||||||||||||||||||||||
Ref 433        GCGTAGTAATCATACACCTCGTTACTGGTAATCGTCGCGGCCACGCCGTAAAACCCCAAC  492

Qry 461        TTCTGAATGTTGACCTCGGATCAGGTAGGAATAC  494
               ||||||||||||||||||||||||||||||||||
Ref 493        TTCTGAATGTTGACCTCGGATCAGGTAGGAATAC  526
```

Reference sequence:
Fusarium incarnatum-equiseti species complex (NRRL 43640; MLST type: 1-a)
GQ505756
Fusarium incarnatum-equiseti species complex (NRRL 43640; MLST type: 1-a), Fusarium incarnatum-equiseti species complex (NRRL 43640; MLST type: 1-a), 1-a, USA TX, Dog nose, n7: 28S ribosomal RNA large subunit (28S - LSU)

Sequence length: 1132

Similarity: 24/24 [100.000 %], Gaps: 0 [0.000 %], Coverage: 24/24 [100.000 %]
Score: 39.6241, Probability: 0.00194015, Direction: +/+

```
Qry 2          GCGGAGGGATCATTACCGAGTTTA  25
               ||||||||||||||||||||||||
Ref 29         GCGGAGGGATCATTACCGAGTTTA  52
```

23	Fusarium incarnatum-equiseti species complex (NRRL 43640; MLST type: 1-a) GQ505756 Fusarium incarnatum-equiseti	721.158	0	100	2	96.761	+/+	****

species complex (NRRL 43640; MLST type: 1-a), Fusarium incarnatum-equiseti species complex (NRRL 43640; MLST type: 1-a), 1-a, USA TX, Dog nose, n4: Internal transcribed spacers (ITS1 and ITS2)

Alignment

Reference sequence:
Fusarium incarnatum-equiseti species complex (NRRL 43640; MLST type: 1-a)
GQ505756
Fusarium incarnatum-equiseti species complex (NRRL 43640; MLST type: 1-a), Fusarium incarnatum-equiseti species complex (NRRL 43640; MLST type: 1-a), 1-a, USA TX, Dog nose, n4: Internal transcribed spacers (ITS1 and ITS2)

Sequence length: 1132

Similarity: 454/454 [100.000 %], Gaps: 0 [0.000 %], Coverage: 454/454 [100.000 %]
Score: 721.158, Probability: 0, Direction: +/+

```
Qry 41         ACATACCTATACGTTGCCTCGGCGGATCAGCCCGCGCCCCGTAAAAAGGGACGGCCCGCC  100
               ||||||||||||||||||||||||||||||||||||||||||||||||||||||||||||
Ref 73         ACATACCTATACGTTGCCTCGGCGGATCAGCCCGCGCCCCGTAAAAAGGGACGGCCCGCC  132

Qry 101        CGAGGACCCCTAAACTCTGTTTTTAGTGGAACTTCTGAGTAAAACAAACAAATAAATCAA  160
               ||||||||||||||||||||||||||||||||||||||||||||||||||||||||||||
Ref 133        CGAGGACCCCTAAACTCTGTTTTTAGTGGAACTTCTGAGTAAAACAAACAAATAAATCAA  192

Qry 161        AACTTTCAACAACGGATCTCTTGGTTCTGGCATCGATGAAGAACGCAGCAAAATGCGATA  220
               ||||||||||||||||||||||||||||||||||||||||||||||||||||||||||||
Ref 193        AACTTTCAACAACGGATCTCTTGGTTCTGGCATCGATGAAGAACGCAGCAAAATGCGATA  252

Qry 221        AGTAATGTGAATTGCAGAATTCAGTGAATCATCGAATCTTTGAACGCACATTGCGCCCGC  280
               ||||||||||||||||||||||||||||||||||||||||||||||||||||||||||||
Ref 253        AGTAATGTGAATTGCAGAATTCAGTGAATCATCGAATCTTTGAACGCACATTGCGCCCGC  312

Qry 281        CAGTATTCTGGCGGGCATGCCTGTTCGAGCGTCATTTCAACCCTCAAGCTCAGCTTGGTG  340
               ||||||||||||||||||||||||||||||||||||||||||||||||||||||||||||
Ref 313        CAGTATTCTGGCGGGCATGCCTGTTCGAGCGTCATTTCAACCCTCAAGCTCAGCTTGGTG  372

Qry 341        TTGGGACTCGCGGTAACCCGCGTTCCCCAAATCGATTGGCGGTCACGTCGAGCTTCCATA  400
               ||||||||||||||||||||||||||||||||||||||||||||||||||||||||||||
Ref 373        TTGGGACTCGCGGTAACCCGCGTTCCCCAAATCGATTGGCGGTCACGTCGAGCTTCCATA  432

Qry 401        GCGTAGTAATCATACACCTCGTTACTGGTAATCGTCGCGGCCACGCCGTAAAACCCCAAC  460
               ||||||||||||||||||||||||||||||||||||||||||||||||||||||||||||
Ref 433        GCGTAGTAATCATACACCTCGTTACTGGTAATCGTCGCGGCCACGCCGTAAAACCCCAAC  492

Qry 461        TTCTGAATGTTGACCTCGGATCAGGTAGGAATAC  494
               ||||||||||||||||||||||||||||||||||
Ref 493        TTCTGAATGTTGACCTCGGATCAGGTAGGAATAC  526
```

Reference sequence:
Fusarium incarnatum-equiseti species complex (NRRL 43640; MLST type: 1-a)
GQ505756
Fusarium incarnatum-equiseti species complex (NRRL 43640; MLST type: 1-a), Fusarium incarnatum-equiseti species complex (NRRL 43640; MLST type: 1-a), 1-a, USA TX, Dog nose, n4: Internal transcribed spacers (ITS1 and ITS2)

Sequence length: 1132

Similarity: 24/24 [100.000 %], Gaps: 0 [0.000 %], Coverage: 24/24 [100.000 %]
Score: 39.6241, Probability: 0.00194015, Direction: +/+

```
Qry 2          GCGGAGGGATCATTACCGAGTTTA  25
               ||||||||||||||||||||||||
Ref 29         GCGGAGGGATCATTACCGAGTTTA  52
```

24	Fusarium incarnatum-equiseti species complex (NRRL 43637;	721.158	0	100	2	96.761	+/+	****

MLST type: 1-a)
GQ505753 Fusarium
incarnatum-equiseti
species complex
(NRRL 43637; MLST
type: 1-a), Fusarium
incarnatum-equiseti
species complex
(NRRL 43637; MLST
type: 1-a), 1-a, USA
PA, Dog, n7: 28S
ribosomal RNA large
subunit (28S - LSU)

Alignment

Reference sequence:
Fusarium incarnatum-equiseti species complex (NRRL 43637; MLST type: 1-a)
GQ505753
Fusarium incarnatum-equiseti species complex (NRRL 43637; MLST type: 1-a), Fusarium incarnatum-equiseti species complex (NRRL 43637; MLST type: 1-a), 1-a, USA PA, Dog, n7: 28S ribosomal RNA large subunit (28S - LSU)

Sequence length: 1132

Similarity: 454/454 [100.000 %], Gaps: 0 [0.000 %], Coverage: 454/454 [100.000 %]
Score: 721.158, Probability: 0, Direction: +/+

```
Qry 41         ACATACCTATACGTTGCCTCGGCGGATCAGCCCGCGCCCCGTAAAAAGGGACGGCCCGCC  100
               ||||||||||||||||||||||||||||||||||||||||||||||||||||||||||||
Ref 73         ACATACCTATACGTTGCCTCGGCGGATCAGCCCGCGCCCCGTAAAAAGGGACGGCCCGCC  132

Qry 101        CGAGGACCCCTAAACTCTGTTTTTAGTGGAACTTCTGAGTAAAACAAACAAATAAATCAA  160
               ||||||||||||||||||||||||||||||||||||||||||||||||||||||||||||
Ref 133        CGAGGACCCCTAAACTCTGTTTTTAGTGGAACTTCTGAGTAAAACAAACAAATAAATCAA  192

Qry 161        AACTTTCAACAACGGATCTCTTGGTTCTGGCATCGATGAAGAACGCAGCAAAATGCGATA  220
               ||||||||||||||||||||||||||||||||||||||||||||||||||||||||||||
Ref 193        AACTTTCAACAACGGATCTCTTGGTTCTGGCATCGATGAAGAACGCAGCAAAATGCGATA  252

Qry 221        AGTAATGTGAATTGCAGAATTCAGTGAATCATCGAATCTTTGAACGCACATTGCGCCCGC  280
               ||||||||||||||||||||||||||||||||||||||||||||||||||||||||||||
Ref 253        AGTAATGTGAATTGCAGAATTCAGTGAATCATCGAATCTTTGAACGCACATTGCGCCCGC  312

Qry 281        CAGTATTCTGGCGGGCATGCCTGTTCGAGCGTCATTTCAACCCTCAAGCTCAGCTTGGTG  340
               ||||||||||||||||||||||||||||||||||||||||||||||||||||||||||||
Ref 313        CAGTATTCTGGCGGGCATGCCTGTTCGAGCGTCATTTCAACCCTCAAGCTCAGCTTGGTG  372

Qry 341        TTGGGACTCGCGGTAACCCGCGTTCCCCAAATCGATTGGCGGTCACGTCGAGCTTCCATA  400
               ||||||||||||||||||||||||||||||||||||||||||||||||||||||||||||
Ref 373        TTGGGACTCGCGGTAACCCGCGTTCCCCAAATCGATTGGCGGTCACGTCGAGCTTCCATA  432

Qry 401        GCGTAGTAATCATACACCTCGTTACTGGTAATCGTCGCGGCCACGCCGTAAAACCCCAAC  460
               ||||||||||||||||||||||||||||||||||||||||||||||||||||||||||||
Ref 433        GCGTAGTAATCATACACCTCGTTACTGGTAATCGTCGCGGCCACGCCGTAAAACCCCAAC  492

Qry 461        TTCTGAATGTTGACCTCGGATCAGGTAGGAATAC  494
               ||||||||||||||||||||||||||||||||||
Ref 493        TTCTGAATGTTGACCTCGGATCAGGTAGGAATAC  526
```

Reference sequence:
Fusarium incarnatum-equiseti species complex (NRRL 43637; MLST type: 1-a)
GQ505753
Fusarium incarnatum-equiseti species complex (NRRL 43637; MLST type: 1-a), Fusarium incarnatum-equiseti species complex (NRRL 43637; MLST type: 1-a), 1-a, USA PA, Dog, n7: 28S ribosomal RNA large subunit (28S - LSU)

Sequence length: 1132

Similarity: 24/24 [100.000 %], Gaps: 0 [0.000 %], Coverage: 24/24 [100.000 %]
Score: 39.6241, Probability: 0.00194015, Direction: +/+

```
Qry 2          GCGGAGGGATCATTACCGAGTTTA  25
               ||||||||||||||||||||||||
Ref 29         GCGGAGGGATCATTACCGAGTTTA  52
```

25	Fusarium incarnatum-equiseti species complex (NRRL 26922; MLST type: 9-c) GQ505690 Fusarium incarnatum-equiseti species complex (NRRL 26922; MLST type: 9-c), Fusarium incarnatum-equiseti species complex (NRRL 26922; MLST type: 9-c), 9-c, France, Soil, n7: 28S ribosomal RNA large subunit (28S - LSU)	721.158	0	100	2	96.761	+/+	****

Alignment

Reference sequence:
Fusarium incarnatum-equiseti species complex (NRRL 26922; MLST type: 9-c)
GQ505690
Fusarium incarnatum-equiseti species complex (NRRL 26922; MLST type: 9-c), Fusarium incarnatum-equiseti species complex (NRRL 26922; MLST type: 9-c), 9-c, France, Soil, n7: 28S ribosomal RNA large subunit (28S - LSU)

Sequence length: 1132

Similarity: 454/454 [100.000 %], Gaps: 0 [0.000 %], Coverage: 454/454 [100.000 %]
Score: 721.158, Probability: 0, Direction: +/+

```
Qry 41        ACATACCTATACGTTGCCTCGGCGGATCAGCCCGCGCCCCGTAAAAAGGGACGGCCCGCC  100
              ||||||||||||||||||||||||||||||||||||||||||||||||||||||||||||
Ref 73        ACATACCTATACGTTGCCTCGGCGGATCAGCCCGCGCCCCGTAAAAAGGGACGGCCCGCC  132

Qry 101       CGAGGACCCCTAAACTCTGTTTTTAGTGGAACTTCTGAGTAAAACAAACAAATAAATCAA  160
              ||||||||||||||||||||||||||||||||||||||||||||||||||||||||||||
Ref 133       CGAGGACCCCTAAACTCTGTTTTTAGTGGAACTTCTGAGTAAAACAAACAAATAAATCAA  192

Qry 161       AACTTTCAACAACGGATCTCTTGGTTCTGGCATCGATGAAGAACGCAGCAAAATGCGATA  220
              ||||||||||||||||||||||||||||||||||||||||||||||||||||||||||||
Ref 193       AACTTTCAACAACGGATCTCTTGGTTCTGGCATCGATGAAGAACGCAGCAAAATGCGATA  252

Qry 221       AGTAATGTGAATTGCAGAATTCAGTGAATCATCGAATCTTTGAACGCACATTGCGCCCGC  280
              ||||||||||||||||||||||||||||||||||||||||||||||||||||||||||||
Ref 253       AGTAATGTGAATTGCAGAATTCAGTGAATCATCGAATCTTTGAACGCACATTGCGCCCGC  312

Qry 281       CAGTATTCTGGCGGGCATGCCTGTTCGAGCGTCATTTCAACCCTCAAGCTCAGCTTGGTG  340
              ||||||||||||||||||||||||||||||||||||||||||||||||||||||||||||
Ref 313       CAGTATTCTGGCGGGCATGCCTGTTCGAGCGTCATTTCAACCCTCAAGCTCAGCTTGGTG  372

Qry 341       TTGGGACTCGCGGTAACCCGCGTTCCCCAAATCGATTGGCGGTCACGTCGAGCTTCCATA  400
              ||||||||||||||||||||||||||||||||||||||||||||||||||||||||||||
Ref 373       TTGGGACTCGCGGTAACCCGCGTTCCCCAAATCGATTGGCGGTCACGTCGAGCTTCCATA  432

Qry 401       GCGTAGTAATCATACACCTCGTTACTGGTAATCGTCGCGGCCACGCCGTAAAACCCCAAC  460
              ||||||||||||||||||||||||||||||||||||||||||||||||||||||||||||
Ref 433       GCGTAGTAATCATACACCTCGTTACTGGTAATCGTCGCGGCCACGCCGTAAAACCCCAAC  492

Qry 461       TTCTGAATGTTGACCTCGGATCAGGTAGGAATAC  494
              ||||||||||||||||||||||||||||||||||
Ref 493       TTCTGAATGTTGACCTCGGATCAGGTAGGAATAC  526
```

Reference sequence:
Fusarium incarnatum-equiseti species complex (NRRL 26922; MLST type: 9-c)
GQ505690
Fusarium incarnatum-equiseti species complex (NRRL 26922; MLST type: 9-c), Fusarium incarnatum-equiseti species complex (NRRL 26922; MLST type: 9-c), 9-c, France, Soil, n7: 28S ribosomal RNA large subunit (28S - LSU)

Sequence length: 1132

Similarity: 24/24 [100.000 %], Gaps: 0 [0.000 %], Coverage: 24/24 [100.000 %]
Score: 39.6241, Probability: 0.00194015, Direction: +/+

```
Qry 2         GCGGAGGGATCATTACCGAGTTTA  25
```

```
                ||||||||||||||||||||||||
Ref 29          GCGGAGGGATCATTACCGAGTTTA  52
```

26	Fusarium incarnatum-equiseti species complex (NRRL 43637; MLST type: 1-a) GQ505753 Fusarium incarnatum-equiseti species complex (NRRL 43637; MLST type: 1-a), Fusarium incarnatum-equiseti species complex (NRRL 43637; MLST type: 1-a), 1-a, USA PA, Dog, n4: Internal transcribed spacers (ITS1 and ITS2)	721.158	0	100	2	96.761	+/+	****

Alignment

Reference sequence:
Fusarium incarnatum-equiseti species complex (NRRL 43637; MLST type: 1-a)
GQ505753
Fusarium incarnatum-equiseti species complex (NRRL 43637; MLST type: 1-a), Fusarium incarnatum-equiseti species complex (NRRL 43637; MLST type: 1-a), 1-a, USA PA, Dog, n4: Internal transcribed spacers (ITS1 and ITS2)

Sequence length: 1132

Similarity: 454/454 [100.000 %], Gaps: 0 [0.000 %], Coverage: 454/454 [100.000 %]
Score: 721.158, Probability: 0, Direction: +/+

```
Qry 41          ACATACCTATACGTTGCCTCGGCGGATCAGCCCGCGCCCCGTAAAAAGGGACGGCCCGCC  100
                ||||||||||||||||||||||||||||||||||||||||||||||||||||||||||||
Ref 73          ACATACCTATACGTTGCCTCGGCGGATCAGCCCGCGCCCCGTAAAAAGGGACGGCCCGCC  132

Qry 101         CGAGGACCCCTAAACTCTGTTTTTAGTGGAACTTCTGAGTAAAACAAACAAATAAATCAA  160
                ||||||||||||||||||||||||||||||||||||||||||||||||||||||||||||
Ref 133         CGAGGACCCCTAAACTCTGTTTTTAGTGGAACTTCTGAGTAAAACAAACAAATAAATCAA  192

Qry 161         AACTTTCAACAACGGATCTCTTGGTTCTGGCATCGATGAAGAACGCAGCAAAATGCGATA  220
                ||||||||||||||||||||||||||||||||||||||||||||||||||||||||||||
Ref 193         AACTTTCAACAACGGATCTCTTGGTTCTGGCATCGATGAAGAACGCAGCAAAATGCGATA  252

Qry 221         AGTAATGTGAATTGCAGAATTCAGTGAATCATCGAATCTTTGAACGCACATTGCGCCCGC  280
                ||||||||||||||||||||||||||||||||||||||||||||||||||||||||||||
Ref 253         AGTAATGTGAATTGCAGAATTCAGTGAATCATCGAATCTTTGAACGCACATTGCGCCCGC  312

Qry 281         CAGTATTCTGGCGGGCATGCCTGTTCGAGCGTCATTTCAACCCTCAAGCTCAGCTTGGTG  340
                ||||||||||||||||||||||||||||||||||||||||||||||||||||||||||||
Ref 313         CAGTATTCTGGCGGGCATGCCTGTTCGAGCGTCATTTCAACCCTCAAGCTCAGCTTGGTG  372

Qry 341         TTGGGACTCGCGGTAACCCGCGTTCCCCAAATCGATTGGCGGTCACGTCGAGCTTCCATA  400
                ||||||||||||||||||||||||||||||||||||||||||||||||||||||||||||
Ref 373         TTGGGACTCGCGGTAACCCGCGTTCCCCAAATCGATTGGCGGTCACGTCGAGCTTCCATA  432

Qry 401         GCGTAGTAATCATACACCTCGTTACTGGTAATCGTCGCGGCCACGCCGTAAAACCCCAAC  460
                ||||||||||||||||||||||||||||||||||||||||||||||||||||||||||||
Ref 433         GCGTAGTAATCATACACCTCGTTACTGGTAATCGTCGCGGCCACGCCGTAAAACCCCAAC  492

Qry 461         TTCTGAATGTTGACCTCGGATCAGGTAGGAATAC  494
                ||||||||||||||||||||||||||||||||||
Ref 493         TTCTGAATGTTGACCTCGGATCAGGTAGGAATAC  526
```

Reference sequence:
Fusarium incarnatum-equiseti species complex (NRRL 43637; MLST type: 1-a)
GQ505753
Fusarium incarnatum-equiseti species complex (NRRL 43637; MLST type: 1-a), Fusarium incarnatum-equiseti species complex (NRRL 43637; MLST type: 1-a), 1-a, USA PA, Dog, n4: Internal transcribed spacers (ITS1 and ITS2)

Sequence length: 1132

Similarity: 24/24 [100.000 %], Gaps: 0 [0.000 %], Coverage: 24/24 [100.000 %]

Score: 39.6241, Probability: 0.00194015, Direction: +/+

```
Qry 2          GCGGAGGGATCATTACCGAGTTTA  25
               ||||||||||||||||||||||||
Ref 29         GCGGAGGGATCATTACCGAGTTTA  52
```

27	Fusarium incarnatum-equiseti species complex (NRRL 43635; MLST type: 13-a) GQ505751 Fusarium incarnatum-equiseti species complex (NRRL 43635; MLST type: 13-a), Fusarium incarnatum-equiseti species complex (NRRL 43635; MLST type: 13-a), 13-a, USA NE, Horse, n4: Internal transcribed spacers (ITS1 and ITS2)	721.158	0	100	2	96.761	+/+	****

Alignment

Reference sequence:
Fusarium incarnatum-equiseti species complex (NRRL 43635; MLST type: 13-a) GQ505751
Fusarium incarnatum-equiseti species complex (NRRL 43635; MLST type: 13-a), Fusarium incarnatum-equiseti species complex (NRRL 43635; MLST type: 13-a), 13-a, USA NE, Horse, n4: Internal transcribed spacers (ITS1 and ITS2)

Sequence length: 1132

Similarity: 454/454 [100.000 %], Gaps: 0 [0.000 %], Coverage: 454/454 [100.000 %]
Score: 721.158, Probability: 0, Direction: +/+

```
Qry 41         ACATACCTATACGTTGCCTCGGCGGATCAGCCCGCGCCCCGTAAAAAGGGACGGCCCGCC  100
               ||||||||||||||||||||||||||||||||||||||||||||||||||||||||||||
Ref 73         ACATACCTATACGTTGCCTCGGCGGATCAGCCCGCGCCCCGTAAAAAGGGACGGCCCGCC  132

Qry 101        CGAGGACCCCTAAACTCTGTTTTTAGTGGAACTTCTGAGTAAAACAAACAAATAAATCAA  160
               ||||||||||||||||||||||||||||||||||||||||||||||||||||||||||||
Ref 133        CGAGGACCCCTAAACTCTGTTTTTAGTGGAACTTCTGAGTAAAACAAACAAATAAATCAA  192

Qry 161        AACTTTCAACAACGGATCTCTTGGTTCTGGCATCGATGAAGAACGCAGCAAAATGCGATA  220
               ||||||||||||||||||||||||||||||||||||||||||||||||||||||||||||
Ref 193        AACTTTCAACAACGGATCTCTTGGTTCTGGCATCGATGAAGAACGCAGCAAAATGCGATA  252

Qry 221        AGTAATGTGAATTGCAGAATTCAGTGAATCATCGAATCTTTGAACGCACATTGCGCCCGC  280
               ||||||||||||||||||||||||||||||||||||||||||||||||||||||||||||
Ref 253        AGTAATGTGAATTGCAGAATTCAGTGAATCATCGAATCTTTGAACGCACATTGCGCCCGC  312

Qry 281        CAGTATTCTGGCGGGCATGCCTGTTCGAGCGTCATTTCAACCCTCAAGCTCAGCTTGGTG  340
               ||||||||||||||||||||||||||||||||||||||||||||||||||||||||||||
Ref 313        CAGTATTCTGGCGGGCATGCCTGTTCGAGCGTCATTTCAACCCTCAAGCTCAGCTTGGTG  372

Qry 341        TTGGGACTCGCGGTAACCCGCGTTCCCCAAATCGATTGGCGGTCACGTCGAGCTTCCATA  400
               ||||||||||||||||||||||||||||||||||||||||||||||||||||||||||||
Ref 373        TTGGGACTCGCGGTAACCCGCGTTCCCCAAATCGATTGGCGGTCACGTCGAGCTTCCATA  432

Qry 401        GCGTAGTAATCATACACCTCGTTACTGGTAATCGTCGCGGCCACGCCGTAAAACCCCAAC  460
               ||||||||||||||||||||||||||||||||||||||||||||||||||||||||||||
Ref 433        GCGTAGTAATCATACACCTCGTTACTGGTAATCGTCGCGGCCACGCCGTAAAACCCCAAC  492

Qry 461        TTCTGAATGTTGACCTCGGATCAGGTAGGAATAC  494
               ||||||||||||||||||||||||||||||||||
Ref 493        TTCTGAATGTTGACCTCGGATCAGGTAGGAATAC  526
```

Reference sequence:
Fusarium incarnatum-equiseti species complex (NRRL 43635; MLST type: 13-a) GQ505751
Fusarium incarnatum-equiseti species complex (NRRL 43635; MLST type: 13-a), Fusarium incarnatum-equiseti species complex (NRRL 43635; MLST type: 13-a), 13-a, USA NE, Horse, n4: Internal transcribed spacers (ITS1 and ITS2)

Sequence length: 1132

Similarity: 24/24 [100.000 %], Gaps: 0 [0.000 %], Coverage: 24/24 [100.000 %]
Score: 39.6241, Probability: 0.00194015, Direction: +/+

```
Qry 2          GCGGAGGGATCATTACCGAGTTTA  25
               ||||||||||||||||||||||||
Ref 29         GCGGAGGGATCATTACCGAGTTTA  52
```

28	Fusarium incarnatum-equiseti species complex (NRRL 43623; MLST type: 5-e) GQ505750 Fusarium incarnatum-equiseti species complex (NRRL 43623; MLST type: 5-e), Fusarium incarnatum-equiseti species complex (NRRL 43623; MLST type: 5-e), 5-e, USA CO, Human maxillary sinus, n7: 28S ribosomal RNA large subunit (28S - LSU)	721.158	0	100	2	96.761	+/+	****

Alignment

Reference sequence:
Fusarium incarnatum-equiseti species complex (NRRL 43623; MLST type: 5-e)
GQ505750
Fusarium incarnatum-equiseti species complex (NRRL 43623; MLST type: 5-e), Fusarium incarnatum-equiseti species complex (NRRL 43623; MLST type: 5-e), 5-e, USA CO, Human maxillary sinus, n7: 28S ribosomal RNA large subunit (28S - LSU)

Sequence length: 1132

Similarity: 454/454 [100.000 %], Gaps: 0 [0.000 %], Coverage: 454/454 [100.000 %]
Score: 721.158, Probability: 0, Direction: +/+

```
Qry 41         ACATACCTATACGTTGCCTCGGCGGATCAGCCCGCGCCCCGTAAAAAGGGACGGCCCGCC  100
               ||||||||||||||||||||||||||||||||||||||||||||||||||||||||||||
Ref 73         ACATACCTATACGTTGCCTCGGCGGATCAGCCCGCGCCCCGTAAAAAGGGACGGCCCGCC  132

Qry 101        CGAGGACCCCTAAACTCTGTTTTTAGTGGAACTTCTGAGTAAAACAAACAAATAAATCAA  160
               ||||||||||||||||||||||||||||||||||||||||||||||||||||||||||||
Ref 133        CGAGGACCCCTAAACTCTGTTTTTAGTGGAACTTCTGAGTAAAACAAACAAATAAATCAA  192

Qry 161        AACTTTCAACAACGGATCTCTTGGTTCTGGCATCGATGAAGAACGCAGCAAAATGCGATA  220
               ||||||||||||||||||||||||||||||||||||||||||||||||||||||||||||
Ref 193        AACTTTCAACAACGGATCTCTTGGTTCTGGCATCGATGAAGAACGCAGCAAAATGCGATA  252

Qry 221        AGTAATGTGAATTGCAGAATTCAGTGAATCATCGAATCTTTGAACGCACATTGCGCCCGC  280
               ||||||||||||||||||||||||||||||||||||||||||||||||||||||||||||
Ref 253        AGTAATGTGAATTGCAGAATTCAGTGAATCATCGAATCTTTGAACGCACATTGCGCCCGC  312

Qry 281        CAGTATTCTGGCGGGCATGCCTGTTCGAGCGTCATTTCAACCCTCAAGCTCAGCTTGGTG  340
               ||||||||||||||||||||||||||||||||||||||||||||||||||||||||||||
Ref 313        CAGTATTCTGGCGGGCATGCCTGTTCGAGCGTCATTTCAACCCTCAAGCTCAGCTTGGTG  372

Qry 341        TTGGGACTCGCGGTAACCCGCGTTCCCCAAATCGATTGGCGGTCACGTCGAGCTTCCATA  400
               ||||||||||||||||||||||||||||||||||||||||||||||||||||||||||||
Ref 373        TTGGGACTCGCGGTAACCCGCGTTCCCCAAATCGATTGGCGGTCACGTCGAGCTTCCATA  432

Qry 401        GCGTAGTAATCATACACCTCGTTACTGGTAATCGTCGCGGCCACGCCGTAAAACCCCAAC  460
               ||||||||||||||||||||||||||||||||||||||||||||||||||||||||||||
Ref 433        GCGTAGTAATCATACACCTCGTTACTGGTAATCGTCGCGGCCACGCCGTAAAACCCCAAC  492

Qry 461        TTCTGAATGTTGACCTCGGATCAGGTAGGAATAC  494
               ||||||||||||||||||||||||||||||||||
Ref 493        TTCTGAATGTTGACCTCGGATCAGGTAGGAATAC  526
```

Reference sequence:
Fusarium incarnatum-equiseti species complex (NRRL 43623; MLST type: 5-e)
GQ505750
Fusarium incarnatum-equiseti species complex (NRRL 43623; MLST type: 5-e), Fusarium

incarnatum-equiseti species complex (NRRL 43623; MLST type: 5-e), 5-e, USA CO, Human maxillary sinus, n7: 28S ribosomal RNA large subunit (28S - LSU)

Sequence length: 1132

Similarity: 24/24 [100.000 %], Gaps: 0 [0.000 %], Coverage: 24/24 [100.000 %]
Score: 39.6241, Probability: 0.00194015, Direction: +/+

```
Qry 2        GCGGAGGGATCATTACCGAGTTTA  25
             ||||||||||||||||||||||||
Ref 29       GCGGAGGGATCATTACCGAGTTTA  52
```

29	Fusarium incarnatum-equiseti species complex (NRRL 43623; MLST type: 5-e) GQ505750 Fusarium incarnatum-equiseti species complex (NRRL 43623; MLST type: 5-e), Fusarium incarnatum-equiseti species complex (NRRL 43623; MLST type: 5-e), 5-e, USA CO, Human maxillary sinus, n4: Internal transcribed spacers (ITS1 and ITS2)	721.158	0	100	2	96.761	+/+	****

Alignment

Reference sequence:
Fusarium incarnatum-equiseti species complex (NRRL 43623; MLST type: 5-e)
GQ505750
Fusarium incarnatum-equiseti species complex (NRRL 43623; MLST type: 5-e), Fusarium incarnatum-equiseti species complex (NRRL 43623; MLST type: 5-e), 5-e, USA CO, Human maxillary sinus, n4: Internal transcribed spacers (ITS1 and ITS2)

Sequence length: 1132

Similarity: 454/454 [100.000 %], Gaps: 0 [0.000 %], Coverage: 454/454 [100.000 %]
Score: 721.158, Probability: 0, Direction: +/+

```
Qry 41       ACATACCTATACGTTGCCTCGGCGGATCAGCCCGCGCCCCGTAAAAAGGGACGGCCCGCC  100
             ||||||||||||||||||||||||||||||||||||||||||||||||||||||||||||
Ref 73       ACATACCTATACGTTGCCTCGGCGGATCAGCCCGCGCCCCGTAAAAAGGGACGGCCCGCC  132

Qry 101      CGAGGACCCCTAAACTCTGTTTTTAGTGGAACTTCTGAGTAAAACAAACAAATAAATCAA  160
             ||||||||||||||||||||||||||||||||||||||||||||||||||||||||||||
Ref 133      CGAGGACCCCTAAACTCTGTTTTTAGTGGAACTTCTGAGTAAAACAAACAAATAAATCAA  192

Qry 161      AACTTTCAACAACGGATCTCTTGGTTCTGGCATCGATGAAGAACGCAGCAAAATGCGATA  220
             ||||||||||||||||||||||||||||||||||||||||||||||||||||||||||||
Ref 193      AACTTTCAACAACGGATCTCTTGGTTCTGGCATCGATGAAGAACGCAGCAAAATGCGATA  252

Qry 221      AGTAATGTGAATTGCAGAATTCAGTGAATCATCGAATCTTTGAACGCACATTGCGCCCGC  280
             ||||||||||||||||||||||||||||||||||||||||||||||||||||||||||||
Ref 253      AGTAATGTGAATTGCAGAATTCAGTGAATCATCGAATCTTTGAACGCACATTGCGCCCGC  312

Qry 281      CAGTATTCTGGCGGGCATGCCTGTTCGAGCGTCATTTCAACCCTCAAGCTCAGCTTGGTG  340
             ||||||||||||||||||||||||||||||||||||||||||||||||||||||||||||
Ref 313      CAGTATTCTGGCGGGCATGCCTGTTCGAGCGTCATTTCAACCCTCAAGCTCAGCTTGGTG  372

Qry 341      TTGGGACTCGCGGTAACCCGCGTTCCCCAAATCGATTGGCGGTCACGTCGAGCTTCCATA  400
             ||||||||||||||||||||||||||||||||||||||||||||||||||||||||||||
Ref 373      TTGGGACTCGCGGTAACCCGCGTTCCCCAAATCGATTGGCGGTCACGTCGAGCTTCCATA  432

Qry 401      GCGTAGTAATCATACACCTCGTTACTGGTAATCGTCGCGGCCACGCCGTAAAACCCCAAC  460
             ||||||||||||||||||||||||||||||||||||||||||||||||||||||||||||
Ref 433      GCGTAGTAATCATACACCTCGTTACTGGTAATCGTCGCGGCCACGCCGTAAAACCCCAAC  492

Qry 461      TTCTGAATGTTGACCTCGGATCAGGTAGGAATAC  494
             ||||||||||||||||||||||||||||||||||
Ref 493      TTCTGAATGTTGACCTCGGATCAGGTAGGAATAC  526
```

Reference sequence:

Fusarium incarnatum-equiseti species complex (NRRL 43623; MLST type: 5-e)
GQ505750
Fusarium incarnatum-equiseti species complex (NRRL 43623; MLST type: 5-e), Fusarium incarnatum-equiseti species complex (NRRL 43623; MLST type: 5-e), 5-e, USA CO, Human maxillary sinus, n4: Internal transcribed spacers (ITS1 and ITS2)

Sequence length: 1132

Similarity: 24/24 [100.000 %], Gaps: 0 [0.000 %], Coverage: 24/24 [100.000 %]
Score: 39.6241, Probability: 0.00194015, Direction: +/+

```
Qry 2          GCGGAGGGATCATTACCGAGTTTA  25
               ||||||||||||||||||||||||
Ref 29         GCGGAGGGATCATTACCGAGTTTA  52
```

30	Fusarium incarnatum-equiseti species complex (NRRL 36478; MLST type: 9-a) GQ505743 Fusarium incarnatum-equiseti species complex (NRRL 36478; MLST type: 9-a), Fusarium incarnatum-equiseti species complex (NRRL 36478; MLST type: 9-a), 9-a, Australia, Pasture soil, n7: 28S ribosomal RNA large subunit (28S - LSU)	721.158	0	100	2	96.761	+/+	****

Alignment

Reference sequence:
Fusarium incarnatum-equiseti species complex (NRRL 36478; MLST type: 9-a)
GQ505743
Fusarium incarnatum-equiseti species complex (NRRL 36478; MLST type: 9-a), Fusarium incarnatum-equiseti species complex (NRRL 36478; MLST type: 9-a), 9-a, Australia, Pasture soil, n7: 28S ribosomal RNA large subunit (28S - LSU)

Sequence length: 1132

Similarity: 454/454 [100.000 %], Gaps: 0 [0.000 %], Coverage: 454/454 [100.000 %]
Score: 721.158, Probability: 0, Direction: +/+

```
Qry 41         ACATACCTATACGTTGCCTCGGCGGATCAGCCCGCGCCCCGTAAAAAGGGACGGCCCGCC  100
               ||||||||||||||||||||||||||||||||||||||||||||||||||||||||||||
Ref 73         ACATACCTATACGTTGCCTCGGCGGATCAGCCCGCGCCCCGTAAAAAGGGACGGCCCGCC  132

Qry 101        CGAGGACCCCTAAACTCTGTTTTTAGTGGAACTTCTGAGTAAAACAAACAAATAAATCAA  160
               ||||||||||||||||||||||||||||||||||||||||||||||||||||||||||||
Ref 133        CGAGGACCCCTAAACTCTGTTTTTAGTGGAACTTCTGAGTAAAACAAACAAATAAATCAA  192

Qry 161        AACTTTCAACAACGGATCTCTTGGTTCTGGCATCGATGAAGAACGCAGCAAAATGCGATA  220
               ||||||||||||||||||||||||||||||||||||||||||||||||||||||||||||
Ref 193        AACTTTCAACAACGGATCTCTTGGTTCTGGCATCGATGAAGAACGCAGCAAAATGCGATA  252

Qry 221        AGTAATGTGAATTGCAGAATTCAGTGAATCATCGAATCTTTGAACGCACATTGCGCCCGC  280
               ||||||||||||||||||||||||||||||||||||||||||||||||||||||||||||
Ref 253        AGTAATGTGAATTGCAGAATTCAGTGAATCATCGAATCTTTGAACGCACATTGCGCCCGC  312

Qry 281        CAGTATTCTGGCGGGCATGCCTGTTCGAGCGTCATTTCAACCCTCAAGCTCAGCTTGGTG  340
               ||||||||||||||||||||||||||||||||||||||||||||||||||||||||||||
Ref 313        CAGTATTCTGGCGGGCATGCCTGTTCGAGCGTCATTTCAACCCTCAAGCTCAGCTTGGTG  372

Qry 341        TTGGGACTCGCGGTAACCCGCGTTCCCCAAATCGATTGGCGGTCACGTCGAGCTTCCATA  400
               ||||||||||||||||||||||||||||||||||||||||||||||||||||||||||||
Ref 373        TTGGGACTCGCGGTAACCCGCGTTCCCCAAATCGATTGGCGGTCACGTCGAGCTTCCATA  432

Qry 401        GCGTAGTAATCATACACCTCGTTACTGGTAATCGTCGCGGCCACGCCGTAAAACCCCAAC  460
               ||||||||||||||||||||||||||||||||||||||||||||||||||||||||||||
Ref 433        GCGTAGTAATCATACACCTCGTTACTGGTAATCGTCGCGGCCACGCCGTAAAACCCCAAC  492

Qry 461        TTCTGAATGTTGACCTCGGATCAGGTAGGAATAC  494
               ||||||||||||||||||||||||||||||||||
```

```
Ref 493        TTCTGAATGTTGACCTCGGATCAGGTAGGAATAC  526
```

Reference sequence:
Fusarium incarnatum-equiseti species complex (NRRL 36478; MLST type: 9-a)
GQ505743
Fusarium incarnatum-equiseti species complex (NRRL 36478; MLST type: 9-a), Fusarium incarnatum-equiseti species complex (NRRL 36478; MLST type: 9-a), 9-a, Australia, Pasture soil, n7: 28S ribosomal RNA large subunit (28S - LSU)

Sequence length: 1132

Similarity: 24/24 [100.000 %], Gaps: 0 [0.000 %], Coverage: 24/24 [100.000 %]
Score: 39.6241, Probability: 0.00194015, Direction: +/+

```
Qry 2          GCGGAGGGATCATTACCGAGTTTA  25
               ||||||||||||||||||||||||
Ref 29         GCGGAGGGATCATTACCGAGTTTA  52
```

31	Fusarium incarnatum-equiseti species complex (NRRL 36478; MLST type: 9-a) GQ505743 Fusarium incarnatum-equiseti species complex (NRRL 36478; MLST type: 9-a), Fusarium incarnatum-equiseti species complex (NRRL 36478; MLST type: 9-a), 9-a, Australia, Pasture soil, n4: Internal transcribed spacers (ITS1 and ITS2)	721.158	0	100	2	96.761	+/+	****

Alignment

Reference sequence:
Fusarium incarnatum-equiseti species complex (NRRL 36478; MLST type: 9-a)
GQ505743
Fusarium incarnatum-equiseti species complex (NRRL 36478; MLST type: 9-a), Fusarium incarnatum-equiseti species complex (NRRL 36478; MLST type: 9-a), 9-a, Australia, Pasture soil, n4: Internal transcribed spacers (ITS1 and ITS2)

Sequence length: 1132

Similarity: 454/454 [100.000 %], Gaps: 0 [0.000 %], Coverage: 454/454 [100.000 %]
Score: 721.158, Probability: 0, Direction: +/+

```
Qry 41         ACATACCTATACGTTGCCTCGGCGGATCAGCCCGCGCCCCGTAAAAAGGGACGGCCCGCC  100
               ||||||||||||||||||||||||||||||||||||||||||||||||||||||||||||
Ref 73         ACATACCTATACGTTGCCTCGGCGGATCAGCCCGCGCCCCGTAAAAAGGGACGGCCCGCC  132

Qry 101        CGAGGACCCCTAAACTCTGTTTTTAGTGGAACTTCTGAGTAAAACAAACAAATAAATCAA  160
               ||||||||||||||||||||||||||||||||||||||||||||||||||||||||||||
Ref 133        CGAGGACCCCTAAACTCTGTTTTTAGTGGAACTTCTGAGTAAAACAAACAAATAAATCAA  192

Qry 161        AACTTTCAACAACGGATCTCTTGGTTCTGGCATCGATGAAGAACGCAGCAAAATGCGATA  220
               ||||||||||||||||||||||||||||||||||||||||||||||||||||||||||||
Ref 193        AACTTTCAACAACGGATCTCTTGGTTCTGGCATCGATGAAGAACGCAGCAAAATGCGATA  252

Qry 221        AGTAATGTGAATTGCAGAATTCAGTGAATCATCGAATCTTTGAACGCACATTGCGCCCGC  280
               ||||||||||||||||||||||||||||||||||||||||||||||||||||||||||||
Ref 253        AGTAATGTGAATTGCAGAATTCAGTGAATCATCGAATCTTTGAACGCACATTGCGCCCGC  312

Qry 281        CAGTATTCTGGCGGGCATGCCTGTTCGAGCGTCATTTCAACCCTCAAGCTCAGCTTGGTG  340
               ||||||||||||||||||||||||||||||||||||||||||||||||||||||||||||
Ref 313        CAGTATTCTGGCGGGCATGCCTGTTCGAGCGTCATTTCAACCCTCAAGCTCAGCTTGGTG  372

Qry 341        TTGGGACTCGCGGTAACCCGCGTTCCCCAAATCGATTGGCGGTCACGTCGAGCTTCCATA  400
               ||||||||||||||||||||||||||||||||||||||||||||||||||||||||||||
Ref 373        TTGGGACTCGCGGTAACCCGCGTTCCCCAAATCGATTGGCGGTCACGTCGAGCTTCCATA  432

Qry 401        GCGTAGTAATCATACACCTCGTTACTGGTAATCGTCGCGGCCACGCCGTAAAACCCCAAC  460
               ||||||||||||||||||||||||||||||||||||||||||||||||||||||||||||
Ref 433        GCGTAGTAATCATACACCTCGTTACTGGTAATCGTCGCGGCCACGCCGTAAAACCCCAAC  492
```

```
Qry 461        TTCTGAATGTTGACCTCGGATCAGGTAGGAATAC  494
               ||||||||||||||||||||||||||||||||||
Ref 493        TTCTGAATGTTGACCTCGGATCAGGTAGGAATAC  526
```

Reference sequence:
Fusarium incarnatum-equiseti species complex (NRRL 36478; MLST type: 9-a)
GQ505743
Fusarium incarnatum-equiseti species complex (NRRL 36478; MLST type: 9-a), Fusarium incarnatum-equiseti species complex (NRRL 36478; MLST type: 9-a), 9-a, Australia, Pasture soil, n4: Internal transcribed spacers (ITS1 and ITS2)

Sequence length: 1132

Similarity: 24/24 [100.000 %], Gaps: 0 [0.000 %], Coverage: 24/24 [100.000 %]
Score: 39.6241, Probability: 0.00194015, Direction: +/+

```
Qry 2          GCGGAGGGATCATTACCGAGTTTA  25
               ||||||||||||||||||||||||
Ref 29         GCGGAGGGATCATTACCGAGTTTA  52
```

32	Fusarium incarnatum-equiseti species complex (NRRL 36448; MLST type: 2-b) GQ505741 Fusarium incarnatum-equiseti species complex (NRRL 36448; MLST type: 2-b), Fusarium incarnatum-equiseti species complex (NRRL 36448; MLST type: 2-b), 2-b, Sudan, Bean (Phaseolus vulgaris) seed, n7: 28S ribosomal RNA large subunit (28S - LSU)	721.158	0	100	2	96.761	+/+	****

Alignment

Reference sequence:
Fusarium incarnatum-equiseti species complex (NRRL 36448; MLST type: 2-b)
GQ505741
Fusarium incarnatum-equiseti species complex (NRRL 36448; MLST type: 2-b), Fusarium incarnatum-equiseti species complex (NRRL 36448; MLST type: 2-b), 2-b, Sudan, Bean (Phaseolus vulgaris) seed, n7: 28S ribosomal RNA large subunit (28S - LSU)

Sequence length: 1132

Similarity: 454/454 [100.000 %], Gaps: 0 [0.000 %], Coverage: 454/454 [100.000 %]
Score: 721.158, Probability: 0, Direction: +/+

```
Qry 41         ACATACCTATACGTTGCCTCGGCGGATCAGCCCGCGCCCCGTAAAAAGGGACGGCCCGCC  100
               ||||||||||||||||||||||||||||||||||||||||||||||||||||||||||||
Ref 73         ACATACCTATACGTTGCCTCGGCGGATCAGCCCGCGCCCCGTAAAAAGGGACGGCCCGCC  132

Qry 101        CGAGGACCCCTAAACTCTGTTTTTAGTGGAACTTCTGAGTAAAACAAACAAATAAATCAA  160
               ||||||||||||||||||||||||||||||||||||||||||||||||||||||||||||
Ref 133        CGAGGACCCCTAAACTCTGTTTTTAGTGGAACTTCTGAGTAAAACAAACAAATAAATCAA  192

Qry 161        AACTTTCAACAACGGATCTCTTGGTTCTGGCATCGATGAAGAACGCAGCAAAATGCGATA  220
               ||||||||||||||||||||||||||||||||||||||||||||||||||||||||||||
Ref 193        AACTTTCAACAACGGATCTCTTGGTTCTGGCATCGATGAAGAACGCAGCAAAATGCGATA  252

Qry 221        AGTAATGTGAATTGCAGAATTCAGTGAATCATCGAATCTTTGAACGCACATTGCGCCCGC  280
               ||||||||||||||||||||||||||||||||||||||||||||||||||||||||||||
Ref 253        AGTAATGTGAATTGCAGAATTCAGTGAATCATCGAATCTTTGAACGCACATTGCGCCCGC  312

Qry 281        CAGTATTCTGGCGGGCATGCCTGTTCGAGCGTCATTTCAACCCTCAAGCTCAGCTTGGTG  340
               ||||||||||||||||||||||||||||||||||||||||||||||||||||||||||||
Ref 313        CAGTATTCTGGCGGGCATGCCTGTTCGAGCGTCATTTCAACCCTCAAGCTCAGCTTGGTG  372

Qry 341        TTGGGACTCGCGGTAACCCGCGTTCCCCAAATCGATTGGCGGTCACGTCGAGCTTCCATA  400
               ||||||||||||||||||||||||||||||||||||||||||||||||||||||||||||
Ref 373        TTGGGACTCGCGGTAACCCGCGTTCCCCAAATCGATTGGCGGTCACGTCGAGCTTCCATA  432
```

```
Qry 401        GCGTAGTAATCATACACCTCGTTACTGGTAATCGTCGCGGCCACGCCGTAAAACCCCAAC  460
               ||||||||||||||||||||||||||||||||||||||||||||||||||||||||||||
Ref 433        GCGTAGTAATCATACACCTCGTTACTGGTAATCGTCGCGGCCACGCCGTAAAACCCCAAC  492

Qry 461        TTCTGAATGTTGACCTCGGATCAGGTAGGAATAC  494
               ||||||||||||||||||||||||||||||||||
Ref 493        TTCTGAATGTTGACCTCGGATCAGGTAGGAATAC  526
```

Reference sequence:
Fusarium incarnatum-equiseti species complex (NRRL 36448; MLST type: 2-b)
GQ505741
Fusarium incarnatum-equiseti species complex (NRRL 36448; MLST type: 2-b), Fusarium incarnatum-equiseti species complex (NRRL 36448; MLST type: 2-b), 2-b, Sudan, Bean (Phaseolus vulgaris) seed, n7: 28S ribosomal RNA large subunit (28S - LSU)

Sequence length: 1132

Similarity: 24/24 [100.000 %], Gaps: 0 [0.000 %], Coverage: 24/24 [100.000 %]
Score: 39.6241, Probability: 0.00194015, Direction: +/+

```
Qry 2          GCGGAGGGATCATTACCGAGTTTA  25
               ||||||||||||||||||||||||
Ref 29         GCGGAGGGATCATTACCGAGTTTA  52
```

33	Fusarium incarnatum-equiseti species complex (NRRL 43635; MLST type: 13-a) GQ505751 Fusarium incarnatum-equiseti species complex (NRRL 43635; MLST type: 13-a), Fusarium incarnatum-equiseti species complex (NRRL 43635; MLST type: 13-a), 13-a, USA NE, Horse, n7: 28S ribosomal RNA large subunit (28S - LSU)	721.158	0	100	2	96.761	+/+	****

Alignment

Reference sequence:
Fusarium incarnatum-equiseti species complex (NRRL 43635; MLST type: 13-a)
GQ505751
Fusarium incarnatum-equiseti species complex (NRRL 43635; MLST type: 13-a), Fusarium incarnatum-equiseti species complex (NRRL 43635; MLST type: 13-a), 13-a, USA NE, Horse, n7: 28S ribosomal RNA large subunit (28S - LSU)

Sequence length: 1132

Similarity: 454/454 [100.000 %], Gaps: 0 [0.000 %], Coverage: 454/454 [100.000 %]
Score: 721.158, Probability: 0, Direction: +/+

```
Qry 41         ACATACCTATACGTTGCCTCGGCGGATCAGCCCGCGCCCCGTAAAAAGGGACGGCCCGCC  100
               ||||||||||||||||||||||||||||||||||||||||||||||||||||||||||||
Ref 73         ACATACCTATACGTTGCCTCGGCGGATCAGCCCGCGCCCCGTAAAAAGGGACGGCCCGCC  132

Qry 101        CGAGGACCCCTAAACTCTGTTTTTAGTGGAACTTCTGAGTAAAACAAACAAATAAATCAA  160
               ||||||||||||||||||||||||||||||||||||||||||||||||||||||||||||
Ref 133        CGAGGACCCCTAAACTCTGTTTTTAGTGGAACTTCTGAGTAAAACAAACAAATAAATCAA  192

Qry 161        AACTTTCAACAACGGATCTCTTGGTTCTGGCATCGATGAAGAACGCAGCAAAATGCGATA  220
               ||||||||||||||||||||||||||||||||||||||||||||||||||||||||||||
Ref 193        AACTTTCAACAACGGATCTCTTGGTTCTGGCATCGATGAAGAACGCAGCAAAATGCGATA  252

Qry 221        AGTAATGTGAATTGCAGAATTCAGTGAATCATCGAATCTTTGAACGCACATTGCGCCCGC  280
               ||||||||||||||||||||||||||||||||||||||||||||||||||||||||||||
Ref 253        AGTAATGTGAATTGCAGAATTCAGTGAATCATCGAATCTTTGAACGCACATTGCGCCCGC  312

Qry 281        CAGTATTCTGGCGGGCATGCCTGTTCGAGCGTCATTTCAACCCTCAAGCTCAGCTTGGTG  340
               ||||||||||||||||||||||||||||||||||||||||||||||||||||||||||||
Ref 313        CAGTATTCTGGCGGGCATGCCTGTTCGAGCGTCATTTCAACCCTCAAGCTCAGCTTGGTG  372
```

```
Qry 341       TTGGGACTCGCGGTAACCCGCGTTCCCCAAATCGATTGGCGGTCACGTCGAGCTTCCATA  400
              ||||||||||||||||||||||||||||||||||||||||||||||||||||||||||||
Ref 373       TTGGGACTCGCGGTAACCCGCGTTCCCCAAATCGATTGGCGGTCACGTCGAGCTTCCATA  432

Qry 401       GCGTAGTAATCATACACCTCGTTACTGGTAATCGTCGCGGCCACGCCGTAAAACCCCAAC  460
              ||||||||||||||||||||||||||||||||||||||||||||||||||||||||||||
Ref 433       GCGTAGTAATCATACACCTCGTTACTGGTAATCGTCGCGGCCACGCCGTAAAACCCCAAC  492

Qry 461       TTCTGAATGTTGACCTCGGATCAGGTAGGAATAC  494
              ||||||||||||||||||||||||||||||||||
Ref 493       TTCTGAATGTTGACCTCGGATCAGGTAGGAATAC  526
```

Reference sequence:
Fusarium incarnatum-equiseti species complex (NRRL 43635; MLST type: 13-a)
GQ505751
Fusarium incarnatum-equiseti species complex (NRRL 43635; MLST type: 13-a), Fusarium incarnatum-equiseti species complex (NRRL 43635; MLST type: 13-a), 13-a, USA NE, Horse, n7: 28S ribosomal RNA large subunit (28S - LSU)

Sequence length: 1132

Similarity: 24/24 [100.000 %], Gaps: 0 [0.000 %], Coverage: 24/24 [100.000 %]
Score: 39.6241, Probability: 0.00194015, Direction: +/+

```
Qry 2         GCGGAGGGATCATTACCGAGTTTA  25
              ||||||||||||||||||||||||
Ref 29        GCGGAGGGATCATTACCGAGTTTA  52
```

34	Fusarium incarnatum-equiseti species complex (NRRL 26922; MLST type: 9-c) GQ505690 Fusarium incarnatum-equiseti species complex (NRRL 26922; MLST type: 9-c), Fusarium incarnatum-equiseti species complex (NRRL 26922; MLST type: 9-c), 9-c, France, Soil, n4: Internal transcribed spacers (ITS1 and ITS2)	721.158	0	100	2	96.761	+/+	****

Alignment

Reference sequence:
Fusarium incarnatum-equiseti species complex (NRRL 26922; MLST type: 9-c)
GQ505690
Fusarium incarnatum-equiseti species complex (NRRL 26922; MLST type: 9-c), Fusarium incarnatum-equiseti species complex (NRRL 26922; MLST type: 9-c), 9-c, France, Soil, n4: Internal transcribed spacers (ITS1 and ITS2)

Sequence length: 1132

Similarity: 454/454 [100.000 %], Gaps: 0 [0.000 %], Coverage: 454/454 [100.000 %]
Score: 721.158, Probability: 0, Direction: +/+

```
Qry 41        ACATACCTATACGTTGCCTCGGCGGATCAGCCCGCGCCCCGTAAAAAGGGACGGCCCGCC  100
              ||||||||||||||||||||||||||||||||||||||||||||||||||||||||||||
Ref 73        ACATACCTATACGTTGCCTCGGCGGATCAGCCCGCGCCCCGTAAAAAGGGACGGCCCGCC  132

Qry 101       CGAGGACCCCTAAACTCTGTTTTTAGTGGAACTTCTGAGTAAAACAAACAAATAAATCAA  160
              ||||||||||||||||||||||||||||||||||||||||||||||||||||||||||||
Ref 133       CGAGGACCCCTAAACTCTGTTTTTAGTGGAACTTCTGAGTAAAACAAACAAATAAATCAA  192

Qry 161       AACTTTCAACAACGGATCTCTTGGTTCTGGCATCGATGAAGAACGCAGCAAAATGCGATA  220
              ||||||||||||||||||||||||||||||||||||||||||||||||||||||||||||
Ref 193       AACTTTCAACAACGGATCTCTTGGTTCTGGCATCGATGAAGAACGCAGCAAAATGCGATA  252

Qry 221       AGTAATGTGAATTGCAGAATTCAGTGAATCATCGAATCTTTGAACGCACATTGCGCCCGC  280
              ||||||||||||||||||||||||||||||||||||||||||||||||||||||||||||
Ref 253       AGTAATGTGAATTGCAGAATTCAGTGAATCATCGAATCTTTGAACGCACATTGCGCCCGC  312

Qry 281       CAGTATTCTGGCGGGCATGCCTGTTCGAGCGTCATTTCAACCCTCAAGCTCAGCTTGGTG  340
```

```
                ||||||||||||||||||||||||||||||||||||||||||||||||||||||||||||
Ref 313         CAGTATTCTGGCGGGCATGCCTGTTCGAGCGTCATTTCAACCCTCAAGCTCAGCTTGGTG  372

Qry 341         TTGGGACTCGCGGTAACCCGCGTTCCCCAAATCGATTGGCGGTCACGTCGAGCTTCCATA  400
                ||||||||||||||||||||||||||||||||||||||||||||||||||||||||||||
Ref 373         TTGGGACTCGCGGTAACCCGCGTTCCCCAAATCGATTGGCGGTCACGTCGAGCTTCCATA  432

Qry 401         GCGTAGTAATCATACACCTCGTTACTGGTAATCGTCGCGGCCACGCCGTAAAACCCCAAC  460
                ||||||||||||||||||||||||||||||||||||||||||||||||||||||||||||
Ref 433         GCGTAGTAATCATACACCTCGTTACTGGTAATCGTCGCGGCCACGCCGTAAAACCCCAAC  492

Qry 461         TTCTGAATGTTGACCTCGGATCAGGTAGGAATAC  494
                ||||||||||||||||||||||||||||||||||
Ref 493         TTCTGAATGTTGACCTCGGATCAGGTAGGAATAC  526
```

Reference sequence:
Fusarium incarnatum-equiseti species complex (NRRL 26922; MLST type: 9-c)
GQ505690
Fusarium incarnatum-equiseti species complex (NRRL 26922; MLST type: 9-c), Fusarium incarnatum-equiseti species complex (NRRL 26922; MLST type: 9-c), 9-c, France, Soil, n4: Internal transcribed spacers (ITS1 and ITS2)

Sequence length: 1132

Similarity: 24/24 [100.000 %], Gaps: 0 [0.000 %], Coverage: 24/24 [100.000 %]
Score: 39.6241, Probability: 0.00194015, Direction: +/+

```
Qry 2           GCGGAGGGATCATTACCGAGTTTA  25
                ||||||||||||||||||||||||
Ref 29          GCGGAGGGATCATTACCGAGTTTA  52
```

35	Fusarium incarnatum-equiseti species complex (NRRL 45996; MLST type: 1-a) GQ505760 Fusarium incarnatum-equiseti species complex (NRRL 45996; MLST type: 1-a), Fusarium incarnatum-equiseti species complex (NRRL 45996; MLST type: 1-a), 1-a, USA NY, Human sinus, n7: 28S ribosomal RNA large subunit (28S - LSU)	732.253	0	97.984	1	99.798	+/+	****

Alignment

Reference sequence:
Fusarium incarnatum-equiseti species complex (NRRL 45996; MLST type: 1-a)
GQ505760
Fusarium incarnatum-equiseti species complex (NRRL 45996; MLST type: 1-a), Fusarium incarnatum-equiseti species complex (NRRL 45996; MLST type: 1-a), 1-a, USA NY, Human sinus, n7: 28S ribosomal RNA large subunit (28S - LSU)

Sequence length: 1130

Similarity: 486/496 [97.984 %], Gaps: 3 [0.605 %], Coverage: 493/496 [99.395 %]
Score: 732.253, Probability: 0, Direction: +/+

```
Qry 2           GCGGAGGGATCATTACCGAGTTTAACTCCAACCCCTGTG---ACATACCTATACGTTGCC  58
                ||||||||||||||||||||||  ||||| |  ||  ||   ||||||||||||||||||
Ref 29          GCGGAGGGATCATTACCGAGTTACACTCCCAAACCCCTGTGAACATACCTATACGTTGCC  88

Qry 59          TCGGCGGATCAGCCCGCGCCCCGTAAAAAGGGACGGCCCGCCCGAGGACCCCTAAACTCT  118
                ||||||||||||||||||||||||||||||||||||||||||||||||||||||||||||
Ref 89          TCGGCGGATCAGCCCGCGCCCCGTAAAAAGGGACGGCCCGCCCGAGGACCCCTAAACTCT  148

Qry 119         GTTTTTAGTGGAACTTCTGAGTAAAACAAACAAATAAATCAAAACTTTCAACAACGGATC  178
                ||||||||||||||||||||||||||||||||||||||||||||||||||||||||||||
Ref 149         GTTTTTAGTGGAACTTCTGAGTAAAACAAACAAATAAATCAAAACTTTCAACAACGGATC  208

Qry 179         TCTTGGTTCTGGCATCGATGAAGAACGCAGCAAAATGCGATAAGTAATGTGAATTGCAGA  238
                ||||||||||||||||||||||||||||||||||||||||||||||||||||||||||||
```

```
Ref 209       TCTTGGTTCTGGCATCGATGAAGAACGCAGCAAAATGCGATAAGTAATGTGAATTGCAGA  268

Qry 239       ATTCAGTGAATCATCGAATCTTTGAACGCACATTGCGCCCGCCAGTATTCTGGCGGGCAT  298
              ||||||||||||||||||||||||||||||||||||||||||||||||||||||||||||
Ref 269       ATTCAGTGAATCATCGAATCTTTGAACGCACATTGCGCCCGCCAGTATTCTGGCGGGCAT  328

Qry 299       GCCTGTTCGAGCGTCATTTCAACCCTCAAGCTCAGCTTGGTGTTGGGACTCGCGGTAACC  358
              ||||||||||||||||||||||||||||||||||||||||||||||||||||||||||||
Ref 329       GCCTGTTCGAGCGTCATTTCAACCCTCAAGCTCAGCTTGGTGTTGGGACTCGCGGTAACC  388

Qry 359       CGCGTTCCCCAAATCGATTGGCGGTCACGTCGAGCTTCCATAGCGTAGTAATCATACACC  418
              ||||||||||||||||||||||||||||||||||||||||||||||||||||||||||||
Ref 389       CGCGTTCCCCAAATCGATTGGCGGTCACGTCGAGCTTCCATAGCGTAGTAATCATACACC  448

Qry 419       TCGTTACTGGTAATCGTCGCGGCCACGCCGTAAAACCCCAACTTCTGAATGTTGACCTCG  478
              ||||||||||||||||||||||||||||||||||||||||||||||||||||||||||||
Ref 449       TCGTTACTGGTAATCGTCGCGGCCACGCCGTAAAACCCCAACTTCTGAATGTTGACCTCG  508

Qry 479       GATCAGGTAGGAATAC  494
              ||||||||||||||||
Ref 509       GATCAGGTAGGAATAC  524
```

36	Fusarium incarnatum-equiseti species complex (NRRL 45996; MLST type: 1-a) GQ505760 Fusarium incarnatum-equiseti species complex (NRRL 45996; MLST type: 1-a), Fusarium incarnatum-equiseti species complex (NRRL 45996; MLST type: 1-a), 1-a, USA NY, Human sinus, n4: Internal transcribed spacers (ITS1 and ITS2)	732.253	0	97.984	1	99.798	+/+	****

Alignment

Reference sequence:
Fusarium incarnatum-equiseti species complex (NRRL 45996; MLST type: 1-a)
GQ505760
Fusarium incarnatum-equiseti species complex (NRRL 45996; MLST type: 1-a), Fusarium incarnatum-equiseti species complex (NRRL 45996; MLST type: 1-a), 1-a, USA NY, Human sinus, n4: Internal transcribed spacers (ITS1 and ITS2)

Sequence length: 1130

Similarity: 486/496 [97.984 %], Gaps: 3 [0.605 %], Coverage: 493/496 [99.395 %]
Score: 732.253, Probability: 0, Direction: +/+

```
Qry 2         GCGGAGGGATCATTACCGAGTTTAACTCCAACCCCTGTG---ACATACCTATACGTTGCC  58
              ||||||||||||||||||||||  ||||| |  ||  ||   ||||||||||||||||||
Ref 29        GCGGAGGGATCATTACCGAGTTACACTCCCAAACCCCTGTGAACATACCTATACGTTGCC  88

Qry 59        TCGGCGGATCAGCCCGCGCCCCGTAAAAAGGGACGGCCCGCCCGAGGACCCCTAAACTCT  118
              ||||||||||||||||||||||||||||||||||||||||||||||||||||||||||||
Ref 89        TCGGCGGATCAGCCCGCGCCCCGTAAAAAGGGACGGCCCGCCCGAGGACCCCTAAACTCT  148

Qry 119       GTTTTTAGTGGAACTTCTGAGTAAAACAAACAAATAAATCAAAACTTTCAACAACGGATC  178
              ||||||||||||||||||||||||||||||||||||||||||||||||||||||||||||
Ref 149       GTTTTTAGTGGAACTTCTGAGTAAAACAAACAAATAAATCAAAACTTTCAACAACGGATC  208

Qry 179       TCTTGGTTCTGGCATCGATGAAGAACGCAGCAAAATGCGATAAGTAATGTGAATTGCAGA  238
              ||||||||||||||||||||||||||||||||||||||||||||||||||||||||||||
Ref 209       TCTTGGTTCTGGCATCGATGAAGAACGCAGCAAAATGCGATAAGTAATGTGAATTGCAGA  268

Qry 239       ATTCAGTGAATCATCGAATCTTTGAACGCACATTGCGCCCGCCAGTATTCTGGCGGGCAT  298
              ||||||||||||||||||||||||||||||||||||||||||||||||||||||||||||
Ref 269       ATTCAGTGAATCATCGAATCTTTGAACGCACATTGCGCCCGCCAGTATTCTGGCGGGCAT  328

Qry 299       GCCTGTTCGAGCGTCATTTCAACCCTCAAGCTCAGCTTGGTGTTGGGACTCGCGGTAACC  358
              ||||||||||||||||||||||||||||||||||||||||||||||||||||||||||||
Ref 329       GCCTGTTCGAGCGTCATTTCAACCCTCAAGCTCAGCTTGGTGTTGGGACTCGCGGTAACC  388
```

```
Qry 359       CGCGTTCCCCAAATCGATTGGCGGTCACGTCGAGCTTCCATAGCGTAGTAATCATACACC  418
              ||||||||||||||||||||||||||||||||||||||||||||||||||||||||||||
Ref 389       CGCGTTCCCCAAATCGATTGGCGGTCACGTCGAGCTTCCATAGCGTAGTAATCATACACC  448

Qry 419       TCGTTACTGGTAATCGTCGCGGCCACGCCGTAAAACCCCAACTTCTGAATGTTGACCTCG  478
              ||||||||||||||||||||||||||||||||||||||||||||||||||||||||||||
Ref 449       TCGTTACTGGTAATCGTCGCGGCCACGCCGTAAAACCCCAACTTCTGAATGTTGACCTCG  508

Qry 479       GATCAGGTAGGAATAC  494
              ||||||||||||||||
Ref 509       GATCAGGTAGGAATAC  524
```

37	Fusarium incarnatum-equiseti species complex (NRRL 43619; MLST type: 15-a) GQ505748 Fusarium incarnatum-equiseti species complex (NRRL 43619; MLST type: 15-a), Fusarium incarnatum-equiseti species complex (NRRL 43619; MLST type: 15-a), 15-a, USA TX, Human finger, n7: 28S ribosomal RNA large subunit (28S - LSU)	717.988	0	99.791	2	96.761	+/+	***

Alignment

Reference sequence:
Fusarium incarnatum-equiseti species complex (NRRL 43619; MLST type: 15-a)
GQ505748
Fusarium incarnatum-equiseti species complex (NRRL 43619; MLST type: 15-a), Fusarium incarnatum-equiseti species complex (NRRL 43619; MLST type: 15-a), 15-a, USA TX, Human finger, n7: 28S ribosomal RNA large subunit (28S - LSU)

Sequence length: 1132

Similarity: 453/454 [99.780 %], Gaps: 0 [0.000 %], Coverage: 454/454 [100.000 %]
Score: 717.988, Probability: 0, Direction: +/+

```
Qry 41        ACATACCTATACGTTGCCTCGGCGGATCAGCCCGCGCCCCGTAAAAAGGGACGGCCCGCC  100
              |||||||||||||||||||||||||||||||||||||||||||||| |||||||||||||
Ref 73        ACATACCTATACGTTGCCTCGGCGGATCAGCCCGCGCCCCGTAAAACGGGACGGCCCGCC  132

Qry 101       CGAGGACCCCTAAACTCTGTTTTTAGTGGAACTTCTGAGTAAAACAAACAAATAAATCAA  160
              ||||||||||||||||||||||||||||||||||||||||||||||||||||||||||||
Ref 133       CGAGGACCCCTAAACTCTGTTTTTAGTGGAACTTCTGAGTAAAACAAACAAATAAATCAA  192

Qry 161       AACTTTCAACAACGGATCTCTTGGTTCTGGCATCGATGAAGAACGCAGCAAAATGCGATA  220
              ||||||||||||||||||||||||||||||||||||||||||||||||||||||||||||
Ref 193       AACTTTCAACAACGGATCTCTTGGTTCTGGCATCGATGAAGAACGCAGCAAAATGCGATA  252

Qry 221       AGTAATGTGAATTGCAGAATTCAGTGAATCATCGAATCTTTGAACGCACATTGCGCCCGC  280
              ||||||||||||||||||||||||||||||||||||||||||||||||||||||||||||
Ref 253       AGTAATGTGAATTGCAGAATTCAGTGAATCATCGAATCTTTGAACGCACATTGCGCCCGC  312

Qry 281       CAGTATTCTGGCGGGCATGCCTGTTCGAGCGTCATTTCAACCCTCAAGCTCAGCTTGGTG  340
              ||||||||||||||||||||||||||||||||||||||||||||||||||||||||||||
Ref 313       CAGTATTCTGGCGGGCATGCCTGTTCGAGCGTCATTTCAACCCTCAAGCTCAGCTTGGTG  372

Qry 341       TTGGGACTCGCGGTAACCCGCGTTCCCCAAATCGATTGGCGGTCACGTCGAGCTTCCATA  400
              ||||||||||||||||||||||||||||||||||||||||||||||||||||||||||||
Ref 373       TTGGGACTCGCGGTAACCCGCGTTCCCCAAATCGATTGGCGGTCACGTCGAGCTTCCATA  432

Qry 401       GCGTAGTAATCATACACCTCGTTACTGGTAATCGTCGCGGCCACGCCGTAAAACCCCAAC  460
              ||||||||||||||||||||||||||||||||||||||||||||||||||||||||||||
Ref 433       GCGTAGTAATCATACACCTCGTTACTGGTAATCGTCGCGGCCACGCCGTAAAACCCCAAC  492

Qry 461       TTCTGAATGTTGACCTCGGATCAGGTAGGAATAC  494
              ||||||||||||||||||||||||||||||||||
Ref 493       TTCTGAATGTTGACCTCGGATCAGGTAGGAATAC  526
```

Reference sequence:
Fusarium incarnatum-equiseti species complex (NRRL 43619; MLST type: 15-a)
GQ505748
Fusarium incarnatum-equiseti species complex (NRRL 43619; MLST type: 15-a), Fusarium incarnatum-equiseti species complex (NRRL 43619; MLST type: 15-a), 15-a, USA TX, Human finger, n7: 28S ribosomal RNA large subunit (28S - LSU)

Sequence length: 1132

Similarity: 24/24 [100.000 %], Gaps: 0 [0.000 %], Coverage: 24/24 [100.000 %]
Score: 39.6241, Probability: 0.00194015, Direction: +/+

```
Qry 2          GCGGAGGGATCATTACCGAGTTTA  25
               ||||||||||||||||||||||||
Ref 29         GCGGAGGGATCATTACCGAGTTTA  52
```

38	Fusarium incarnatum-equiseti species complex (NRRL 43619; MLST type: 15-a) GQ505748 Fusarium incarnatum-equiseti species complex (NRRL 43619; MLST type: 15-a), Fusarium incarnatum-equiseti species complex (NRRL 43619; MLST type: 15-a), 15-a, USA TX, Human finger, n4: Internal transcribed spacers (ITS1 and ITS2)	717.988	0	99.791	2	96.761	+/+	***

Alignment

Reference sequence:
Fusarium incarnatum-equiseti species complex (NRRL 43619; MLST type: 15-a)
GQ505748
Fusarium incarnatum-equiseti species complex (NRRL 43619; MLST type: 15-a), Fusarium incarnatum-equiseti species complex (NRRL 43619; MLST type: 15-a), 15-a, USA TX, Human finger, n4: Internal transcribed spacers (ITS1 and ITS2)

Sequence length: 1132

Similarity: 453/454 [99.780 %], Gaps: 0 [0.000 %], Coverage: 454/454 [100.000 %]
Score: 717.988, Probability: 0, Direction: +/+

```
Qry 41         ACATACCTATACGTTGCCTCGGCGGATCAGCCCGCGCCCCGTAAAAAGGGACGGCCCGCC  100
               ||||||||||||||||||||||||||||||||||||||||||||| ||||||||||||||
Ref 73         ACATACCTATACGTTGCCTCGGCGGATCAGCCCGCGCCCCGTAAAACGGGACGGCCCGCC  132

Qry 101        CGAGGACCCCTAAACTCTGTTTTTAGTGGAACTTCTGAGTAAAACAAACAAATAAATCAA  160
               ||||||||||||||||||||||||||||||||||||||||||||||||||||||||||||
Ref 133        CGAGGACCCCTAAACTCTGTTTTTAGTGGAACTTCTGAGTAAAACAAACAAATAAATCAA  192

Qry 161        AACTTTCAACAACGGATCTCTTGGTTCTGGCATCGATGAAGAACGCAGCAAAATGCGATA  220
               ||||||||||||||||||||||||||||||||||||||||||||||||||||||||||||
Ref 193        AACTTTCAACAACGGATCTCTTGGTTCTGGCATCGATGAAGAACGCAGCAAAATGCGATA  252

Qry 221        AGTAATGTGAATTGCAGAATTCAGTGAATCATCGAATCTTTGAACGCACATTGCGCCCGC  280
               ||||||||||||||||||||||||||||||||||||||||||||||||||||||||||||
Ref 253        AGTAATGTGAATTGCAGAATTCAGTGAATCATCGAATCTTTGAACGCACATTGCGCCCGC  312

Qry 281        CAGTATTCTGGCGGGCATGCCTGTTCGAGCGTCATTTCAACCCTCAAGCTCAGCTTGGTG  340
               ||||||||||||||||||||||||||||||||||||||||||||||||||||||||||||
Ref 313        CAGTATTCTGGCGGGCATGCCTGTTCGAGCGTCATTTCAACCCTCAAGCTCAGCTTGGTG  372

Qry 341        TTGGGACTCGCGGTAACCCGCGTTCCCCAAATCGATTGGCGGTCACGTCGAGCTTCCATA  400
               ||||||||||||||||||||||||||||||||||||||||||||||||||||||||||||
Ref 373        TTGGGACTCGCGGTAACCCGCGTTCCCCAAATCGATTGGCGGTCACGTCGAGCTTCCATA  432

Qry 401        GCGTAGTAATCATACACCTCGTTACTGGTAATCGTCGCGGCCACGCCGTAAAACCCCAAC  460
               ||||||||||||||||||||||||||||||||||||||||||||||||||||||||||||
Ref 433        GCGTAGTAATCATACACCTCGTTACTGGTAATCGTCGCGGCCACGCCGTAAAACCCCAAC  492

Qry 461        TTCTGAATGTTGACCTCGGATCAGGTAGGAATAC  494
```

```
              ||||||||||||||||||||||||||||||||||
Ref 493       TTCTGAATGTTGACCTCGGATCAGGTAGGAATAC  526
```

Reference sequence:
Fusarium incarnatum-equiseti species complex (NRRL 43619; MLST type: 15-a)
GQ505748
Fusarium incarnatum-equiseti species complex (NRRL 43619; MLST type: 15-a), Fusarium incarnatum-equiseti species complex (NRRL 43619; MLST type: 15-a), 15-a, USA TX, Human finger, n4: Internal transcribed spacers (ITS1 and ITS2)

Sequence length: 1132

Similarity: 24/24 [100.000 %], Gaps: 0 [0.000 %], Coverage: 24/24 [100.000 %]
Score: 39.6241, Probability: 0.00194015, Direction: +/+

```
Qry 2         GCGGAGGGATCATTACCGAGTTTA  25
              ||||||||||||||||||||||||
Ref 29        GCGGAGGGATCATTACCGAGTTTA  52
```

39	Fusarium incarnatum-equiseti species complex (NRRL 43297; MLST type: 24-b) GQ505746 Fusarium incarnatum-equiseti species complex (NRRL 43297; MLST type: 24-b), Fusarium incarnatum-equiseti species complex (NRRL 43297; MLST type: 24-b), 24-b, USA CT, Spartina rhizomes, n7: 28S ribosomal RNA large subunit (28S - LSU)	717.988	0	99.791	2	96.761	+/+	***

Alignment

Reference sequence:
Fusarium incarnatum-equiseti species complex (NRRL 43297; MLST type: 24-b)
GQ505746
Fusarium incarnatum-equiseti species complex (NRRL 43297; MLST type: 24-b), Fusarium incarnatum-equiseti species complex (NRRL 43297; MLST type: 24-b), 24-b, USA CT, Spartina rhizomes, n7: 28S ribosomal RNA large subunit (28S - LSU)

Sequence length: 1132

Similarity: 453/454 [99.780 %], Gaps: 0 [0.000 %], Coverage: 454/454 [100.000 %]
Score: 717.988, Probability: 0, Direction: +/+

```
Qry 41        ACATACCTATACGTTGCCTCGGCGGATCAGCCCGCGCCCCGTAAAAAGGGACGGCCCGCC  100
              |||||||||||||||||||||||||||||||||||||||||||||| |||||||||||||
Ref 73        ACATACCTATACGTTGCCTCGGCGGATCAGCCCGCGCCCCGTAAAACGGGACGGCCCGCC  132

Qry 101       CGAGGACCCCTAAACTCTGTTTTTAGTGGAACTTCTGAGTAAAACAAACAAATAAATCAA  160
              ||||||||||||||||||||||||||||||||||||||||||||||||||||||||||||
Ref 133       CGAGGACCCCTAAACTCTGTTTTTAGTGGAACTTCTGAGTAAAACAAACAAATAAATCAA  192

Qry 161       AACTTTCAACAACGGATCTCTTGGTTCTGGCATCGATGAAGAACGCAGCAAAATGCGATA  220
              ||||||||||||||||||||||||||||||||||||||||||||||||||||||||||||
Ref 193       AACTTTCAACAACGGATCTCTTGGTTCTGGCATCGATGAAGAACGCAGCAAAATGCGATA  252

Qry 221       AGTAATGTGAATTGCAGAATTCAGTGAATCATCGAATCTTTGAACGCACATTGCGCCCGC  280
              ||||||||||||||||||||||||||||||||||||||||||||||||||||||||||||
Ref 253       AGTAATGTGAATTGCAGAATTCAGTGAATCATCGAATCTTTGAACGCACATTGCGCCCGC  312

Qry 281       CAGTATTCTGGCGGGCATGCCTGTTCGAGCGTCATTTCAACCCTCAAGCTCAGCTTGGTG  340
              ||||||||||||||||||||||||||||||||||||||||||||||||||||||||||||
Ref 313       CAGTATTCTGGCGGGCATGCCTGTTCGAGCGTCATTTCAACCCTCAAGCTCAGCTTGGTG  372

Qry 341       TTGGGACTCGCGGTAACCCGCGTTCCCCAAATCGATTGGCGGTCACGTCGAGCTTCCATA  400
              ||||||||||||||||||||||||||||||||||||||||||||||||||||||||||||
Ref 373       TTGGGACTCGCGGTAACCCGCGTTCCCCAAATCGATTGGCGGTCACGTCGAGCTTCCATA  432

Qry 401       GCGTAGTAATCATACACCTCGTTACTGGTAATCGTCGCGGCCACGCCGTAAAACCCCAAC  460
```

```
              ||||||||||||||||||||||||||||||||||||||||||||||||||||||||||||
Ref 433       GCGTAGTAATCATACACCTCGTTACTGGTAATCGTCGCGGCCACGCCGTAAAACCCCAAC  492

Qry 461       TTCTGAATGTTGACCTCGGATCAGGTAGGAATAC  494
              ||||||||||||||||||||||||||||||||||
Ref 493       TTCTGAATGTTGACCTCGGATCAGGTAGGAATAC  526
```

Reference sequence:
[Fusarium incarnatum-equiseti species complex (NRRL 43297; MLST type: 24-b)](#)
[GQ505746](#)
Fusarium incarnatum-equiseti species complex (NRRL 43297; MLST type: 24-b), Fusarium incarnatum-equiseti species complex (NRRL 43297; MLST type: 24-b), 24-b, USA CT, Spartina rhizomes, n7: 28S ribosomal RNA large subunit (28S - LSU)

Sequence length: 1132

Similarity: 24/24 [100.000 %], Gaps: 0 [0.000 %], Coverage: 24/24 [100.000 %]
Score: 39.6241, Probability: 0.00194015, Direction: +/+

```
Qry 2         GCGGAGGGATCATTACCGAGTTTA  25
              ||||||||||||||||||||||||
Ref 29        GCGGAGGGATCATTACCGAGTTTA  52
```

40	Fusarium incarnatum-equiseti species complex (NRRL 43297; MLST type: 24-b) GQ505746 Fusarium incarnatum-equiseti species complex (NRRL 43297; MLST type: 24-b), Fusarium incarnatum-equiseti species complex (NRRL 43297; MLST type: 24-b), 24-b, USA CT, Spartina rhizomes, n4: Internal transcribed spacers (ITS1 and ITS2)	717.988	0	99.791	2	96.761	+/+	***

Alignment

Reference sequence:
[Fusarium incarnatum-equiseti species complex (NRRL 43297; MLST type: 24-b)](#)
[GQ505746](#)
Fusarium incarnatum-equiseti species complex (NRRL 43297; MLST type: 24-b), Fusarium incarnatum-equiseti species complex (NRRL 43297; MLST type: 24-b), 24-b, USA CT, Spartina rhizomes, n4: Internal transcribed spacers (ITS1 and ITS2)

Sequence length: 1132

Similarity: 453/454 [99.780 %], Gaps: 0 [0.000 %], Coverage: 454/454 [100.000 %]
Score: 717.988, Probability: 0, Direction: +/+

```
Qry 41        ACATACCTATACGTTGCCTCGGCGGATCAGCCCGCGCCCCGTAAAAAGGGACGGCCCGCC  100
              |||||||||||||||||||||||||||||||||||||||||||||| |||||||||||||
Ref 73        ACATACCTATACGTTGCCTCGGCGGATCAGCCCGCGCCCCGTAAAACGGGACGGCCCGCC  132

Qry 101       CGAGGACCCCTAAACTCTGTTTTTAGTGGAACTTCTGAGTAAAACAAACAAATAAATCAA  160
              ||||||||||||||||||||||||||||||||||||||||||||||||||||||||||||
Ref 133       CGAGGACCCCTAAACTCTGTTTTTAGTGGAACTTCTGAGTAAAACAAACAAATAAATCAA  192

Qry 161       AACTTTCAACAACGGATCTCTTGGTTCTGGCATCGATGAAGAACGCAGCAAAATGCGATA  220
              ||||||||||||||||||||||||||||||||||||||||||||||||||||||||||||
Ref 193       AACTTTCAACAACGGATCTCTTGGTTCTGGCATCGATGAAGAACGCAGCAAAATGCGATA  252

Qry 221       AGTAATGTGAATTGCAGAATTCAGTGAATCATCGAATCTTTGAACGCACATTGCGCCCGC  280
              ||||||||||||||||||||||||||||||||||||||||||||||||||||||||||||
Ref 253       AGTAATGTGAATTGCAGAATTCAGTGAATCATCGAATCTTTGAACGCACATTGCGCCCGC  312

Qry 281       CAGTATTCTGGCGGGCATGCCTGTTCGAGCGTCATTTCAACCCTCAAGCTCAGCTTGGTG  340
              ||||||||||||||||||||||||||||||||||||||||||||||||||||||||||||
Ref 313       CAGTATTCTGGCGGGCATGCCTGTTCGAGCGTCATTTCAACCCTCAAGCTCAGCTTGGTG  372

Qry 341       TTGGGACTCGCGGTAACCCGCGTTCCCCAAATCGATTGGCGGTCACGTCGAGCTTCCATA  400
```

```
               ||||||||||||||||||||||||||||||||||||||||||||||||||||||||||||
Ref 373        TTGGGACTCGCGGTAACCCGCGTTCCCCAAATCGATTGGCGGTCACGTCGAGCTTCCATA  432

Qry 401        GCGTAGTAATCATACACCTCGTTACTGGTAATCGTCGCGGCCACGCCGTAAAACCCCAAC  460
               ||||||||||||||||||||||||||||||||||||||||||||||||||||||||||||
Ref 433        GCGTAGTAATCATACACCTCGTTACTGGTAATCGTCGCGGCCACGCCGTAAAACCCCAAC  492

Qry 461        TTCTGAATGTTGACCTCGGATCAGGTAGGAATAC  494
               ||||||||||||||||||||||||||||||||||
Ref 493        TTCTGAATGTTGACCTCGGATCAGGTAGGAATAC  526
```

Reference sequence:
Fusarium incarnatum-equiseti species complex (NRRL 43297; MLST type: 24-b)
GQ505746
Fusarium incarnatum-equiseti species complex (NRRL 43297; MLST type: 24-b), Fusarium incarnatum-equiseti species complex (NRRL 43297; MLST type: 24-b), 24-b, USA CT, Spartina rhizomes, n4: Internal transcribed spacers (ITS1 and ITS2)

Sequence length: 1132

Similarity: 24/24 [100.000 %], Gaps: 0 [0.000 %], Coverage: 24/24 [100.000 %]
Score: 39.6241, Probability: 0.00194015, Direction: +/+

```
Qry 2          GCGGAGGGATCATTACCGAGTTTA  25
               ||||||||||||||||||||||||
Ref 29         GCGGAGGGATCATTACCGAGTTTA  52
```

41	Fusarium incarnatum-equiseti species complex (NRRL 36548; MLST type: 17-b) GQ505744 Fusarium incarnatum-equiseti species complex (NRRL 36548; MLST type: 17-b), Fusarium incarnatum-equiseti species complex (NRRL 36548; MLST type: 17-b), 17-b, Congo, Banana, n7: 28S ribosomal RNA large subunit (28S - LSU)	717.988	0	99.791	2	96.761	+/+	***

Alignment

Reference sequence:
Fusarium incarnatum-equiseti species complex (NRRL 36548; MLST type: 17-b)
GQ505744
Fusarium incarnatum-equiseti species complex (NRRL 36548; MLST type: 17-b), Fusarium incarnatum-equiseti species complex (NRRL 36548; MLST type: 17-b), 17-b, Congo, Banana, n7: 28S ribosomal RNA large subunit (28S - LSU)

Sequence length: 1132

Similarity: 453/454 [99.780 %], Gaps: 0 [0.000 %], Coverage: 454/454 [100.000 %]
Score: 717.988, Probability: 0, Direction: +/+

```
Qry 41         ACATACCTATACGTTGCCTCGGCGGATCAGCCCGCGCCCCGTAAAAAGGGACGGCCCGCC  100
               ||||||||||||||||||||||||||||||||||||||||||||| ||||||||||||||
Ref 73         ACATACCTATACGTTGCCTCGGCGGATCAGCCCGCGCCCCGTAAAACGGGACGGCCCGCC  132

Qry 101        CGAGGACCCCTAAACTCTGTTTTTAGTGGAACTTCTGAGTAAAACAAACAAATAAATCAA  160
               ||||||||||||||||||||||||||||||||||||||||||||||||||||||||||||
Ref 133        CGAGGACCCCTAAACTCTGTTTTTAGTGGAACTTCTGAGTAAAACAAACAAATAAATCAA  192

Qry 161        AACTTTCAACAACGGATCTCTTGGTTCTGGCATCGATGAAGAACGCAGCAAAATGCGATA  220
               ||||||||||||||||||||||||||||||||||||||||||||||||||||||||||||
Ref 193        AACTTTCAACAACGGATCTCTTGGTTCTGGCATCGATGAAGAACGCAGCAAAATGCGATA  252

Qry 221        AGTAATGTGAATTGCAGAATTCAGTGAATCATCGAATCTTTGAACGCACATTGCGCCCGC  280
               ||||||||||||||||||||||||||||||||||||||||||||||||||||||||||||
Ref 253        AGTAATGTGAATTGCAGAATTCAGTGAATCATCGAATCTTTGAACGCACATTGCGCCCGC  312

Qry 281        CAGTATTCTGGCGGGCATGCCTGTTCGAGCGTCATTTCAACCCTCAAGCTCAGCTTGGTG  340
               ||||||||||||||||||||||||||||||||||||||||||||||||||||||||||||
```

```
Ref 313        CAGTATTCTGGCGGGCATGCCTGTTCGAGCGTCATTTCAACCCTCAAGCTCAGCTTGGTG  372

Qry 341        TTGGGACTCGCGGTAACCCGCGTTCCCCAAATCGATTGGCGGTCACGTCGAGCTTCCATA  400
               ||||||||||||||||||||||||||||||||||||||||||||||||||||||||||||
Ref 373        TTGGGACTCGCGGTAACCCGCGTTCCCCAAATCGATTGGCGGTCACGTCGAGCTTCCATA  432

Qry 401        GCGTAGTAATCATACACCTCGTTACTGGTAATCGTCGCGGCCACGCCGTAAAACCCCAAC  460
               ||||||||||||||||||||||||||||||||||||||||||||||||||||||||||||
Ref 433        GCGTAGTAATCATACACCTCGTTACTGGTAATCGTCGCGGCCACGCCGTAAAACCCCAAC  492

Qry 461        TTCTGAATGTTGACCTCGGATCAGGTAGGAATAC  494
               ||||||||||||||||||||||||||||||||||
Ref 493        TTCTGAATGTTGACCTCGGATCAGGTAGGAATAC  526
```

Reference sequence:
Fusarium incarnatum-equiseti species complex (NRRL 36548; MLST type: 17-b)
GQ505744
Fusarium incarnatum-equiseti species complex (NRRL 36548; MLST type: 17-b), Fusarium incarnatum-equiseti species complex (NRRL 36548; MLST type: 17-b), 17-b, Congo, Banana, n7: 28S ribosomal RNA large subunit (28S - LSU)

Sequence length: 1132

Similarity: 24/24 [100.000 %], Gaps: 0 [0.000 %], Coverage: 24/24 [100.000 %]
Score: 39.6241, Probability: 0.00194015, Direction: +/+

```
Qry 2          GCGGAGGGATCATTACCGAGTTTA  25
               ||||||||||||||||||||||||
Ref 29         GCGGAGGGATCATTACCGAGTTTA  52
```

42	Fusarium incarnatum-equiseti species complex (NRRL 36548; MLST type: 17-b) GQ505744 Fusarium incarnatum-equiseti species complex (NRRL 36548; MLST type: 17-b), Fusarium incarnatum-equiseti species complex (NRRL 36548; MLST type: 17-b), 17-b, Congo, Banana, n4: Internal transcribed spacers (ITS1 and ITS2)	717.988	0	99.791	2	96.761	+/+	***

Alignment

Reference sequence:
Fusarium incarnatum-equiseti species complex (NRRL 36548; MLST type: 17-b)
GQ505744
Fusarium incarnatum-equiseti species complex (NRRL 36548; MLST type: 17-b), Fusarium incarnatum-equiseti species complex (NRRL 36548; MLST type: 17-b), 17-b, Congo, Banana, n4: Internal transcribed spacers (ITS1 and ITS2)

Sequence length: 1132

Similarity: 453/454 [99.780 %], Gaps: 0 [0.000 %], Coverage: 454/454 [100.000 %]
Score: 717.988, Probability: 0, Direction: +/+

```
Qry 41         ACATACCTATACGTTGCCTCGGCGGATCAGCCCGCGCCCCGTAAAAAGGGACGGCCCGCC  100
               |||||||||||||||||||||||||||||||||||||||||||||| |||||||||||||
Ref 73         ACATACCTATACGTTGCCTCGGCGGATCAGCCCGCGCCCCGTAAAACGGGACGGCCCGCC  132

Qry 101        CGAGGACCCCTAAACTCTGTTTTTAGTGGAACTTCTGAGTAAAACAAACAAATAAATCAA  160
               ||||||||||||||||||||||||||||||||||||||||||||||||||||||||||||
Ref 133        CGAGGACCCCTAAACTCTGTTTTTAGTGGAACTTCTGAGTAAAACAAACAAATAAATCAA  192

Qry 161        AACTTTCAACAACGGATCTCTTGGTTCTGGCATCGATGAAGAACGCAGCAAAATGCGATA  220
               ||||||||||||||||||||||||||||||||||||||||||||||||||||||||||||
Ref 193        AACTTTCAACAACGGATCTCTTGGTTCTGGCATCGATGAAGAACGCAGCAAAATGCGATA  252

Qry 221        AGTAATGTGAATTGCAGAATTCAGTGAATCATCGAATCTTTGAACGCACATTGCGCCCGC  280
               ||||||||||||||||||||||||||||||||||||||||||||||||||||||||||||
Ref 253        AGTAATGTGAATTGCAGAATTCAGTGAATCATCGAATCTTTGAACGCACATTGCGCCCGC  312
```

```
Qry 281       CAGTATTCTGGCGGGCATGCCTGTTCGAGCGTCATTTCAACCCTCAAGCTCAGCTTGGTG  340
              ||||||||||||||||||||||||||||||||||||||||||||||||||||||||||||
Ref 313       CAGTATTCTGGCGGGCATGCCTGTTCGAGCGTCATTTCAACCCTCAAGCTCAGCTTGGTG  372

Qry 341       TTGGGACTCGCGGTAACCCGCGTTCCCCAAATCGATTGGCGGTCACGTCGAGCTTCCATA  400
              ||||||||||||||||||||||||||||||||||||||||||||||||||||||||||||
Ref 373       TTGGGACTCGCGGTAACCCGCGTTCCCCAAATCGATTGGCGGTCACGTCGAGCTTCCATA  432

Qry 401       GCGTAGTAATCATACACCTCGTTACTGGTAATCGTCGCGGCCACGCCGTAAAACCCCAAC  460
              ||||||||||||||||||||||||||||||||||||||||||||||||||||||||||||
Ref 433       GCGTAGTAATCATACACCTCGTTACTGGTAATCGTCGCGGCCACGCCGTAAAACCCCAAC  492

Qry 461       TTCTGAATGTTGACCTCGGATCAGGTAGGAATAC  494
              ||||||||||||||||||||||||||||||||||
Ref 493       TTCTGAATGTTGACCTCGGATCAGGTAGGAATAC  526
```

Reference sequence:
Fusarium incarnatum-equiseti species complex (NRRL 36548; MLST type: 17-b)
GQ505744
Fusarium incarnatum-equiseti species complex (NRRL 36548; MLST type: 17-b), Fusarium incarnatum-equiseti species complex (NRRL 36548; MLST type: 17-b), 17-b, Congo, Banana, n4: Internal transcribed spacers (ITS1 and ITS2)

Sequence length: 1132

Similarity: 24/24 [100.000 %], Gaps: 0 [0.000 %], Coverage: 24/24 [100.000 %]
Score: 39.6241, Probability: 0.00194015, Direction: +/+

```
Qry 2         GCGGAGGGATCATTACCGAGTTTA  25
              ||||||||||||||||||||||||
Ref 29        GCGGAGGGATCATTACCGAGTTTA  52
```

43	Fusarium incarnatum-equiseti species complex (NRRL 36372; MLST type: 11-a) GQ505738 Fusarium incarnatum-equiseti species complex (NRRL 36372; MLST type: 11-a), Fusarium incarnatum-equiseti species complex (NRRL 36372; MLST type: 11-a), 11-a, Netherlands, Air, n7: 28S ribosomal RNA large subunit (28S - LSU)	717.988	0	99.791	2	96.761	+/+	***

Alignment

Reference sequence:
Fusarium incarnatum-equiseti species complex (NRRL 36372; MLST type: 11-a)
GQ505738
Fusarium incarnatum-equiseti species complex (NRRL 36372; MLST type: 11-a), Fusarium incarnatum-equiseti species complex (NRRL 36372; MLST type: 11-a), 11-a, Netherlands, Air, n7: 28S ribosomal RNA large subunit (28S - LSU)

Sequence length: 1132

Similarity: 453/454 [99.780 %], Gaps: 0 [0.000 %], Coverage: 454/454 [100.000 %]
Score: 717.988, Probability: 0, Direction: +/+

```
Qry 41        ACATACCTATACGTTGCCTCGGCGGATCAGCCCGCGCCCCGTAAAAAGGGACGGCCCGCC  100
              |||||||||||||||||||||||||||||| |||||||||||||||||||||||||||||
Ref 73        ACATACCTATACGTTGCCTCGGCGGATCAGTCCGCGCCCCGTAAAAAGGGACGGCCCGCC  132

Qry 101       CGAGGACCCCTAAACTCTGTTTTTAGTGGAACTTCTGAGTAAAACAAACAAATAAATCAA  160
              ||||||||||||||||||||||||||||||||||||||||||||||||||||||||||||
Ref 133       CGAGGACCCCTAAACTCTGTTTTTAGTGGAACTTCTGAGTAAAACAAACAAATAAATCAA  192

Qry 161       AACTTTCAACAACGGATCTCTTGGTTCTGGCATCGATGAAGAACGCAGCAAAATGCGATA  220
              ||||||||||||||||||||||||||||||||||||||||||||||||||||||||||||
Ref 193       AACTTTCAACAACGGATCTCTTGGTTCTGGCATCGATGAAGAACGCAGCAAAATGCGATA  252
```

```
Qry 221       AGTAATGTGAATTGCAGAATTCAGTGAATCATCGAATCTTTGAACGCACATTGCGCCCGC  280
              ||||||||||||||||||||||||||||||||||||||||||||||||||||||||||||
Ref 253       AGTAATGTGAATTGCAGAATTCAGTGAATCATCGAATCTTTGAACGCACATTGCGCCCGC  312

Qry 281       CAGTATTCTGGCGGGCATGCCTGTTCGAGCGTCATTTCAACCCTCAAGCTCAGCTTGGTG  340
              ||||||||||||||||||||||||||||||||||||||||||||||||||||||||||||
Ref 313       CAGTATTCTGGCGGGCATGCCTGTTCGAGCGTCATTTCAACCCTCAAGCTCAGCTTGGTG  372

Qry 341       TTGGGACTCGCGGTAACCCGCGTTCCCCAAATCGATTGGCGGTCACGTCGAGCTTCCATA  400
              ||||||||||||||||||||||||||||||||||||||||||||||||||||||||||||
Ref 373       TTGGGACTCGCGGTAACCCGCGTTCCCCAAATCGATTGGCGGTCACGTCGAGCTTCCATA  432

Qry 401       GCGTAGTAATCATACACCTCGTTACTGGTAATCGTCGCGGCCACGCCGTAAAACCCCAAC  460
              ||||||||||||||||||||||||||||||||||||||||||||||||||||||||||||
Ref 433       GCGTAGTAATCATACACCTCGTTACTGGTAATCGTCGCGGCCACGCCGTAAAACCCCAAC  492

Qry 461       TTCTGAATGTTGACCTCGGATCAGGTAGGAATAC  494
              ||||||||||||||||||||||||||||||||||
Ref 493       TTCTGAATGTTGACCTCGGATCAGGTAGGAATAC  526
```

Reference sequence:
Fusarium incarnatum-equiseti species complex (NRRL 36372; MLST type: 11-a)
GQ505738
Fusarium incarnatum-equiseti species complex (NRRL 36372; MLST type: 11-a), Fusarium incarnatum-equiseti species complex (NRRL 36372; MLST type: 11-a), 11-a, Netherlands, Air, n7: 28S ribosomal RNA large subunit (28S - LSU)

Sequence length: 1132

Similarity: 24/24 [100.000 %], Gaps: 0 [0.000 %], Coverage: 24/24 [100.000 %]
Score: 39.6241, Probability: 0.00194015, Direction: +/+

```
Qry 2         GCGGAGGGATCATTACCGAGTTTA  25
              ||||||||||||||||||||||||
Ref 29        GCGGAGGGATCATTACCGAGTTTA  52
```

44	Fusarium incarnatum-equiseti species complex (NRRL 36372; MLST type: 11-a) GQ505738 Fusarium incarnatum-equiseti species complex (NRRL 36372; MLST type: 11-a), Fusarium incarnatum-equiseti species complex (NRRL 36372; MLST type: 11-a), 11-a, Netherlands, Air, n4: Internal transcribed spacers (ITS1 and ITS2)	717.988	0	99.791	2	96.761	+/+	***

Alignment

Reference sequence:
Fusarium incarnatum-equiseti species complex (NRRL 36372; MLST type: 11-a)
GQ505738
Fusarium incarnatum-equiseti species complex (NRRL 36372; MLST type: 11-a), Fusarium incarnatum-equiseti species complex (NRRL 36372; MLST type: 11-a), 11-a, Netherlands, Air, n4: Internal transcribed spacers (ITS1 and ITS2)

Sequence length: 1132

Similarity: 453/454 [99.780 %], Gaps: 0 [0.000 %], Coverage: 454/454 [100.000 %]
Score: 717.988, Probability: 0, Direction: +/+

```
Qry 41        ACATACCTATACGTTGCCTCGGCGGATCAGCCCGCGCCCCGTAAAAAGGGACGGCCCGCC  100
              |||||||||||||||||||||||||||||| |||||||||||||||||||||||||||||
Ref 73        ACATACCTATACGTTGCCTCGGCGGATCAGTCCGCGCCCCGTAAAAAGGGACGGCCCGCC  132

Qry 101       CGAGGACCCCTAAACTCTGTTTTTAGTGGAACTTCTGAGTAAAACAAACAAATAAATCAA  160
              ||||||||||||||||||||||||||||||||||||||||||||||||||||||||||||
Ref 133       CGAGGACCCCTAAACTCTGTTTTTAGTGGAACTTCTGAGTAAAACAAACAAATAAATCAA  192

Qry 161       AACTTTCAACAACGGATCTCTTGGTTCTGGCATCGATGAAGAACGCAGCAAAATGCGATA  220
```

```
               ||||||||||||||||||||||||||||||||||||||||||||||||||||||||||||
Ref 193        AACTTTCAACAACGGATCTCTTGGTTCTGGCATCGATGAAGAACGCAGCAAAATGCGATA  252

Qry 221        AGTAATGTGAATTGCAGAATTCAGTGAATCATCGAATCTTTGAACGCACATTGCGCCCGC  280
               ||||||||||||||||||||||||||||||||||||||||||||||||||||||||||||
Ref 253        AGTAATGTGAATTGCAGAATTCAGTGAATCATCGAATCTTTGAACGCACATTGCGCCCGC  312

Qry 281        CAGTATTCTGGCGGGCATGCCTGTTCGAGCGTCATTTCAACCCTCAAGCTCAGCTTGGTG  340
               ||||||||||||||||||||||||||||||||||||||||||||||||||||||||||||
Ref 313        CAGTATTCTGGCGGGCATGCCTGTTCGAGCGTCATTTCAACCCTCAAGCTCAGCTTGGTG  372

Qry 341        TTGGGACTCGCGGTAACCCGCGTTCCCCAAATCGATTGGCGGTCACGTCGAGCTTCCATA  400
               ||||||||||||||||||||||||||||||||||||||||||||||||||||||||||||
Ref 373        TTGGGACTCGCGGTAACCCGCGTTCCCCAAATCGATTGGCGGTCACGTCGAGCTTCCATA  432

Qry 401        GCGTAGTAATCATACACCTCGTTACTGGTAATCGTCGCGGCCACGCCGTAAAACCCCAAC  460
               ||||||||||||||||||||||||||||||||||||||||||||||||||||||||||||
Ref 433        GCGTAGTAATCATACACCTCGTTACTGGTAATCGTCGCGGCCACGCCGTAAAACCCCAAC  492

Qry 461        TTCTGAATGTTGACCTCGGATCAGGTAGGAATAC  494
               ||||||||||||||||||||||||||||||||||
Ref 493        TTCTGAATGTTGACCTCGGATCAGGTAGGAATAC  526
```

Reference sequence:
Fusarium incarnatum-equiseti species complex (NRRL 36372; MLST type: 11-a)
GQ505738
Fusarium incarnatum-equiseti species complex (NRRL 36372; MLST type: 11-a), Fusarium incarnatum-equiseti species complex (NRRL 36372; MLST type: 11-a), 11-a, Netherlands, Air, n4: Internal transcribed spacers (ITS1 and ITS2)

Sequence length: 1132

Similarity: 24/24 [100.000 %], Gaps: 0 [0.000 %], Coverage: 24/24 [100.000 %]
Score: 39.6241, Probability: 0.00194015, Direction: +/+

```
Qry 2          GCGGAGGGATCATTACCGAGTTTA  25
               ||||||||||||||||||||||||
Ref 29         GCGGAGGGATCATTACCGAGTTTA  52
```

45	Fusarium incarnatum-equiseti species complex (NRRL 34056; MLST type: 16-b) GQ505729 Fusarium incarnatum-equiseti species complex (NRRL 34056; MLST type: 16-b), Fusarium incarnatum-equiseti species complex (NRRL 34056; MLST type: 16-b), 16-b, USA IL, Human bronchial wash, n7: 28S ribosomal RNA large subunit (28S - LSU)	717.988	0	99.791	2	96.761	+/+	***

Alignment

Reference sequence:
Fusarium incarnatum-equiseti species complex (NRRL 34056; MLST type: 16-b)
GQ505729
Fusarium incarnatum-equiseti species complex (NRRL 34056; MLST type: 16-b), Fusarium incarnatum-equiseti species complex (NRRL 34056; MLST type: 16-b), 16-b, USA IL, Human bronchial wash, n7: 28S ribosomal RNA large subunit (28S - LSU)

Sequence length: 1132

Similarity: 453/454 [99.780 %], Gaps: 0 [0.000 %], Coverage: 454/454 [100.000 %]
Score: 717.988, Probability: 0, Direction: +/+

```
Qry 41         ACATACCTATACGTTGCCTCGGCGGATCAGCCCGCGCCCCGTAAAAAGGGACGGCCCGCC  100
               |||||||||||||||||||||||||||||||||||||||||||||| |||||||||||||
Ref 73         ACATACCTATACGTTGCCTCGGCGGATCAGCCCGCGCCCCGTAAAACGGGACGGCCCGCC  132

Qry 101        CGAGGACCCCTAAACTCTGTTTTTAGTGGAACTTCTGAGTAAAACAAACAAATAAATCAA  160
```

```
                ||||||||||||||||||||||||||||||||||||||||||||||||||||||||||||
Ref 133         CGAGGACCCCTAAACTCTGTTTTTAGTGGAACTTCTGAGTAAAACAAACAAATAAATCAA  192

Qry 161         AACTTTCAACAACGGATCTCTTGGTTCTGGCATCGATGAAGAACGCAGCAAAATGCGATA  220
                ||||||||||||||||||||||||||||||||||||||||||||||||||||||||||||
Ref 193         AACTTTCAACAACGGATCTCTTGGTTCTGGCATCGATGAAGAACGCAGCAAAATGCGATA  252

Qry 221         AGTAATGTGAATTGCAGAATTCAGTGAATCATCGAATCTTTGAACGCACATTGCGCCCGC  280
                ||||||||||||||||||||||||||||||||||||||||||||||||||||||||||||
Ref 253         AGTAATGTGAATTGCAGAATTCAGTGAATCATCGAATCTTTGAACGCACATTGCGCCCGC  312

Qry 281         CAGTATTCTGGCGGGCATGCCTGTTCGAGCGTCATTTCAACCCTCAAGCTCAGCTTGGTG  340
                ||||||||||||||||||||||||||||||||||||||||||||||||||||||||||||
Ref 313         CAGTATTCTGGCGGGCATGCCTGTTCGAGCGTCATTTCAACCCTCAAGCTCAGCTTGGTG  372

Qry 341         TTGGGACTCGCGGTAACCCGCGTTCCCCAAATCGATTGGCGGTCACGTCGAGCTTCCATA  400
                ||||||||||||||||||||||||||||||||||||||||||||||||||||||||||||
Ref 373         TTGGGACTCGCGGTAACCCGCGTTCCCCAAATCGATTGGCGGTCACGTCGAGCTTCCATA  432

Qry 401         GCGTAGTAATCATACACCTCGTTACTGGTAATCGTCGCGGCCACGCCGTAAAACCCCAAC  460
                ||||||||||||||||||||||||||||||||||||||||||||||||||||||||||||
Ref 433         GCGTAGTAATCATACACCTCGTTACTGGTAATCGTCGCGGCCACGCCGTAAAACCCCAAC  492

Qry 461         TTCTGAATGTTGACCTCGGATCAGGTAGGAATAC  494
                ||||||||||||||||||||||||||||||||||
Ref 493         TTCTGAATGTTGACCTCGGATCAGGTAGGAATAC  526
```

Reference sequence:
Fusarium incarnatum-equiseti species complex (NRRL 34056; MLST type: 16-b)
GQ505729
Fusarium incarnatum-equiseti species complex (NRRL 34056; MLST type: 16-b), Fusarium incarnatum-equiseti species complex (NRRL 34056; MLST type: 16-b), 16-b, USA IL, Human bronchial wash, n7: 28S ribosomal RNA large subunit (28S - LSU)

Sequence length: 1132

Similarity: 24/24 [100.000 %], Gaps: 0 [0.000 %], Coverage: 24/24 [100.000 %]
Score: 39.6241, Probability: 0.00194015, Direction: +/+

```
Qry 2           GCGGAGGGATCATTACCGAGTTTA  25
                ||||||||||||||||||||||||
Ref 29          GCGGAGGGATCATTACCGAGTTTA  52
```

46	Fusarium incarnatum-equiseti species complex (NRRL 34056; MLST type: 16-b) GQ505729 Fusarium incarnatum-equiseti species complex (NRRL 34056; MLST type: 16-b), Fusarium incarnatum-equiseti species complex (NRRL 34056; MLST type: 16-b), 16-b, USA IL, Human bronchial wash, n4: Internal transcribed spacers (ITS1 and ITS2)	717.988	0	99.791	2	96.761	+/+	***

Alignment

Reference sequence:
Fusarium incarnatum-equiseti species complex (NRRL 34056; MLST type: 16-b)
GQ505729
Fusarium incarnatum-equiseti species complex (NRRL 34056; MLST type: 16-b), Fusarium incarnatum-equiseti species complex (NRRL 34056; MLST type: 16-b), 16-b, USA IL, Human bronchial wash, n4: Internal transcribed spacers (ITS1 and ITS2)

Sequence length: 1132

Similarity: 453/454 [99.780 %], Gaps: 0 [0.000 %], Coverage: 454/454 [100.000 %]
Score: 717.988, Probability: 0, Direction: +/+

```
Qry 41          ACATACCTATACGTTGCCTCGGCGGATCAGCCCGCGCCCCGTAAAAAGGGACGGCCCGCC  100
```

```
              |||||||||||||||||||||||||||||||||||||||||||||| |||||||||||||
Ref 73        ACATACCTATACGTTGCCTCGGCGGATCAGCCCGCGCCCCGTAAAACGGGACGGCCCGCC  132

Qry 101       CGAGGACCCCTAAACTCTGTTTTTAGTGGAACTTCTGAGTAAAACAAACAAATAAATCAA  160
              ||||||||||||||||||||||||||||||||||||||||||||||||||||||||||||
Ref 133       CGAGGACCCCTAAACTCTGTTTTTAGTGGAACTTCTGAGTAAAACAAACAAATAAATCAA  192

Qry 161       AACTTTCAACAACGGATCTCTTGGTTCTGGCATCGATGAAGAACGCAGCAAAATGCGATA  220
              ||||||||||||||||||||||||||||||||||||||||||||||||||||||||||||
Ref 193       AACTTTCAACAACGGATCTCTTGGTTCTGGCATCGATGAAGAACGCAGCAAAATGCGATA  252

Qry 221       AGTAATGTGAATTGCAGAATTCAGTGAATCATCGAATCTTTGAACGCACATTGCGCCCGC  280
              ||||||||||||||||||||||||||||||||||||||||||||||||||||||||||||
Ref 253       AGTAATGTGAATTGCAGAATTCAGTGAATCATCGAATCTTTGAACGCACATTGCGCCCGC  312

Qry 281       CAGTATTCTGGCGGGCATGCCTGTTCGAGCGTCATTTCAACCCTCAAGCTCAGCTTGGTG  340
              ||||||||||||||||||||||||||||||||||||||||||||||||||||||||||||
Ref 313       CAGTATTCTGGCGGGCATGCCTGTTCGAGCGTCATTTCAACCCTCAAGCTCAGCTTGGTG  372

Qry 341       TTGGGACTCGCGGTAACCCGCGTTCCCCAAATCGATTGGCGGTCACGTCGAGCTTCCATA  400
              ||||||||||||||||||||||||||||||||||||||||||||||||||||||||||||
Ref 373       TTGGGACTCGCGGTAACCCGCGTTCCCCAAATCGATTGGCGGTCACGTCGAGCTTCCATA  432

Qry 401       GCGTAGTAATCATACACCTCGTTACTGGTAATCGTCGCGGCCACGCCGTAAAACCCCAAC  460
              ||||||||||||||||||||||||||||||||||||||||||||||||||||||||||||
Ref 433       GCGTAGTAATCATACACCTCGTTACTGGTAATCGTCGCGGCCACGCCGTAAAACCCCAAC  492

Qry 461       TTCTGAATGTTGACCTCGGATCAGGTAGGAATAC  494
              ||||||||||||||||||||||||||||||||||
Ref 493       TTCTGAATGTTGACCTCGGATCAGGTAGGAATAC  526
```

Reference sequence:
Fusarium incarnatum-equiseti species complex (NRRL 34056; MLST type: 16-b)
GQ505729
Fusarium incarnatum-equiseti species complex (NRRL 34056; MLST type: 16-b), Fusarium incarnatum-equiseti species complex (NRRL 34056; MLST type: 16-b), 16-b, USA IL, Human bronchial wash, n4: Internal transcribed spacers (ITS1 and ITS2)

Sequence length: 1132

Similarity: 24/24 [100.000 %], Gaps: 0 [0.000 %], Coverage: 24/24 [100.000 %]
Score: 39.6241, Probability: 0.00194015, Direction: +/+

```
Qry 2         GCGGAGGGATCATTACCGAGTTTA  25
              ||||||||||||||||||||||||
Ref 29        GCGGAGGGATCATTACCGAGTTTA  52
```

47	Fusarium incarnatum-equiseti species complex (NRRL 34039; MLST type: 1-b) GQ505728 Fusarium incarnatum-equiseti species complex (NRRL 34039; MLST type: 1-b), Fusarium incarnatum-equiseti species complex (NRRL 34039; MLST type: 1-b), 1-b, USA CT, Human, n7: 28S ribosomal RNA large subunit (28S - LSU)	717.988	0	99.791	2	96.761	+/+	***

Alignment

Reference sequence:
Fusarium incarnatum-equiseti species complex (NRRL 34039; MLST type: 1-b)
GQ505728
Fusarium incarnatum-equiseti species complex (NRRL 34039; MLST type: 1-b), Fusarium incarnatum-equiseti species complex (NRRL 34039; MLST type: 1-b), 1-b, USA CT, Human, n7: 28S ribosomal RNA large subunit (28S - LSU)

Sequence length: 1132

Similarity: 453/454 [99.780 %], Gaps: 0 [0.000 %], Coverage: 454/454 [100.000 %]
Score: 717.988, Probability: 0, Direction: +/+

```
Qry 41         ACATACCTATACGTTGCCTCGGCGGATCAGCCCGCGCCCCGTAAAAAGGGACGGCCCGCC  100
               |||||||||||||||||||||||||||||||||||||||||||| |||||||||||||||
Ref 73         ACATACCTATACGTTGCCTCGGCGGATCAGCCCGCGCCCCGTAACAAGGGACGGCCCGCC  132

Qry 101        CGAGGACCCCTAAACTCTGTTTTTAGTGGAACTTCTGAGTAAAACAAACAAATAAATCAA  160
               ||||||||||||||||||||||||||||||||||||||||||||||||||||||||||||
Ref 133        CGAGGACCCCTAAACTCTGTTTTTAGTGGAACTTCTGAGTAAAACAAACAAATAAATCAA  192

Qry 161        AACTTTCAACAACGGATCTCTTGGTTCTGGCATCGATGAAGAACGCAGCAAAATGCGATA  220
               ||||||||||||||||||||||||||||||||||||||||||||||||||||||||||||
Ref 193        AACTTTCAACAACGGATCTCTTGGTTCTGGCATCGATGAAGAACGCAGCAAAATGCGATA  252

Qry 221        AGTAATGTGAATTGCAGAATTCAGTGAATCATCGAATCTTTGAACGCACATTGCGCCCGC  280
               ||||||||||||||||||||||||||||||||||||||||||||||||||||||||||||
Ref 253        AGTAATGTGAATTGCAGAATTCAGTGAATCATCGAATCTTTGAACGCACATTGCGCCCGC  312

Qry 281        CAGTATTCTGGCGGGCATGCCTGTTCGAGCGTCATTTCAACCCTCAAGCTCAGCTTGGTG  340
               ||||||||||||||||||||||||||||||||||||||||||||||||||||||||||||
Ref 313        CAGTATTCTGGCGGGCATGCCTGTTCGAGCGTCATTTCAACCCTCAAGCTCAGCTTGGTG  372

Qry 341        TTGGGACTCGCGGTAACCCGCGTTCCCCAAATCGATTGGCGGTCACGTCGAGCTTCCATA  400
               ||||||||||||||||||||||||||||||||||||||||||||||||||||||||||||
Ref 373        TTGGGACTCGCGGTAACCCGCGTTCCCCAAATCGATTGGCGGTCACGTCGAGCTTCCATA  432

Qry 401        GCGTAGTAATCATACACCTCGTTACTGGTAATCGTCGCGGCCACGCCGTAAAACCCCAAC  460
               ||||||||||||||||||||||||||||||||||||||||||||||||||||||||||||
Ref 433        GCGTAGTAATCATACACCTCGTTACTGGTAATCGTCGCGGCCACGCCGTAAAACCCCAAC  492

Qry 461        TTCTGAATGTTGACCTCGGATCAGGTAGGAATAC  494
               ||||||||||||||||||||||||||||||||||
Ref 493        TTCTGAATGTTGACCTCGGATCAGGTAGGAATAC  526
```

Reference sequence:
Fusarium incarnatum-equiseti species complex (NRRL 34039; MLST type: 1-b)
GQ505728
Fusarium incarnatum-equiseti species complex (NRRL 34039; MLST type: 1-b), Fusarium incarnatum-equiseti species complex (NRRL 34039; MLST type: 1-b), 1-b, USA CT, Human, n7: 28S ribosomal RNA large subunit (28S - LSU)

Sequence length: 1132

Similarity: 24/24 [100.000 %], Gaps: 0 [0.000 %], Coverage: 24/24 [100.000 %]
Score: 39.6241, Probability: 0.00194015, Direction: +/+

```
Qry 2          GCGGAGGGATCATTACCGAGTTTA  25
               ||||||||||||||||||||||||
Ref 29         GCGGAGGGATCATTACCGAGTTTA  52
```

48	Fusarium incarnatum-equiseti species complex (NRRL 34039; MLST type: 1-b) GQ505728 Fusarium incarnatum-equiseti species complex (NRRL 34039; MLST type: 1-b), Fusarium incarnatum-equiseti species complex (NRRL 34039; MLST type: 1-b), 1-b, USA CT, Human, n4: Internal transcribed spacers (ITS1 and ITS2)	717.988	0	99.791	2	96.761	+/+	***

Alignment

Reference sequence:
Fusarium incarnatum-equiseti species complex (NRRL 34039; MLST type: 1-b)
GQ505728
Fusarium incarnatum-equiseti species complex (NRRL 34039; MLST type: 1-b), Fusarium incarnatum-equiseti species complex (NRRL 34039; MLST type: 1-b), 1-b, USA CT, Human, n4: Internal transcribed spacers (ITS1 and ITS2)

Sequence length: 1132

```
Similarity: 453/454 [99.780 %], Gaps: 0 [0.000 %], Coverage: 454/454 [100.000 %]
Score: 717.988, Probability: 0, Direction: +/+

Qry 41         ACATACCTATACGTTGCCTCGGCGGATCAGCCCGCGCCCCGTAAAAAGGGACGGCCCGCC  100
               |||||||||||||||||||||||||||||||||||||||||||| |||||||||||||||
Ref 73         ACATACCTATACGTTGCCTCGGCGGATCAGCCCGCGCCCCGTAACAAGGGACGGCCCGCC  132

Qry 101        CGAGGACCCCTAAACTCTGTTTTTAGTGGAACTTCTGAGTAAAACAAACAAATAAATCAA  160
               ||||||||||||||||||||||||||||||||||||||||||||||||||||||||||||
Ref 133        CGAGGACCCCTAAACTCTGTTTTTAGTGGAACTTCTGAGTAAAACAAACAAATAAATCAA  192

Qry 161        AACTTTCAACAACGGATCTCTTGGTTCTGGCATCGATGAAGAACGCAGCAAAATGCGATA  220
               ||||||||||||||||||||||||||||||||||||||||||||||||||||||||||||
Ref 193        AACTTTCAACAACGGATCTCTTGGTTCTGGCATCGATGAAGAACGCAGCAAAATGCGATA  252

Qry 221        AGTAATGTGAATTGCAGAATTCAGTGAATCATCGAATCTTTGAACGCACATTGCGCCCGC  280
               ||||||||||||||||||||||||||||||||||||||||||||||||||||||||||||
Ref 253        AGTAATGTGAATTGCAGAATTCAGTGAATCATCGAATCTTTGAACGCACATTGCGCCCGC  312

Qry 281        CAGTATTCTGGCGGGCATGCCTGTTCGAGCGTCATTTCAACCCTCAAGCTCAGCTTGGTG  340
               ||||||||||||||||||||||||||||||||||||||||||||||||||||||||||||
Ref 313        CAGTATTCTGGCGGGCATGCCTGTTCGAGCGTCATTTCAACCCTCAAGCTCAGCTTGGTG  372

Qry 341        TTGGGACTCGCGGTAACCCGCGTTCCCCAAATCGATTGGCGGTCACGTCGAGCTTCCATA  400
               ||||||||||||||||||||||||||||||||||||||||||||||||||||||||||||
Ref 373        TTGGGACTCGCGGTAACCCGCGTTCCCCAAATCGATTGGCGGTCACGTCGAGCTTCCATA  432

Qry 401        GCGTAGTAATCATACACCTCGTTACTGGTAATCGTCGCGGCCACGCCGTAAAACCCCAAC  460
               ||||||||||||||||||||||||||||||||||||||||||||||||||||||||||||
Ref 433        GCGTAGTAATCATACACCTCGTTACTGGTAATCGTCGCGGCCACGCCGTAAAACCCCAAC  492

Qry 461        TTCTGAATGTTGACCTCGGATCAGGTAGGAATAC  494
               ||||||||||||||||||||||||||||||||||
Ref 493        TTCTGAATGTTGACCTCGGATCAGGTAGGAATAC  526
```

Reference sequence:
Fusarium incarnatum-equiseti species complex (NRRL 34039; MLST type: 1-b)
GQ505728
Fusarium incarnatum-equiseti species complex (NRRL 34039; MLST type: 1-b), Fusarium incarnatum-equiseti species complex (NRRL 34039; MLST type: 1-b), 1-b, USA CT, Human, n4: Internal transcribed spacers (ITS1 and ITS2)

Sequence length: 1132

```
Similarity: 24/24 [100.000 %], Gaps: 0 [0.000 %], Coverage: 24/24 [100.000 %]
Score: 39.6241, Probability: 0.00194015, Direction: +/+

Qry 2          GCGGAGGGATCATTACCGAGTTTA  25
               ||||||||||||||||||||||||
Ref 29         GCGGAGGGATCATTACCGAGTTTA  52
```

49	Fusarium incarnatum-equiseti species complex (NRRL 34011; MLST type: 15-a) GQ505723 Fusarium incarnatum-equiseti species complex (NRRL 34011; MLST type: 15-a), Fusarium incarnatum-equiseti species complex (NRRL 34011; MLST type: 15-a), 15-a, USA TX, Human sputum, n7: 28S ribosomal RNA large subunit (28S - LSU)	717.988	0	99.791	2	96.761	+/+	***

Alignment

Reference sequence:
Fusarium incarnatum-equiseti species complex (NRRL 34011; MLST type: 15-a)
GQ505723
Fusarium incarnatum-equiseti species complex (NRRL 34011; MLST type: 15-a), Fusarium incarnatum-equiseti species complex (NRRL 34011; MLST type: 15-a), 15-a, USA TX,

Human sputum, n7: 28S ribosomal RNA large subunit (28S - LSU)

Sequence length: 1132

Similarity: 453/454 [99.780 %], Gaps: 0 [0.000 %], Coverage: 454/454 [100.000 %]
Score: 717.988, Probability: 0, Direction: +/+

```
Qry 41       ACATACCTATACGTTGCCTCGGCGGATCAGCCCGCGCCCCGTAAAAAGGGACGGCCCGCC  100
             ||||||||||||||||||||||||||||||||||||||||||||||| ||||||||||||||
Ref 73       ACATACCTATACGTTGCCTCGGCGGATCAGCCCGCGCCCCGTAAAACGGGACGGCCCGCC  132

Qry 101      CGAGGACCCCTAAACTCTGTTTTTAGTGGAACTTCTGAGTAAAACAAACAAATAAATCAA  160
             ||||||||||||||||||||||||||||||||||||||||||||||||||||||||||||
Ref 133      CGAGGACCCCTAAACTCTGTTTTTAGTGGAACTTCTGAGTAAAACAAACAAATAAATCAA  192

Qry 161      AACTTTCAACAACGGATCTCTTGGTTCTGGCATCGATGAAGAACGCAGCAAAATGCGATA  220
             ||||||||||||||||||||||||||||||||||||||||||||||||||||||||||||
Ref 193      AACTTTCAACAACGGATCTCTTGGTTCTGGCATCGATGAAGAACGCAGCAAAATGCGATA  252

Qry 221      AGTAATGTGAATTGCAGAATTCAGTGAATCATCGAATCTTTGAACGCACATTGCGCCCGC  280
             ||||||||||||||||||||||||||||||||||||||||||||||||||||||||||||
Ref 253      AGTAATGTGAATTGCAGAATTCAGTGAATCATCGAATCTTTGAACGCACATTGCGCCCGC  312

Qry 281      CAGTATTCTGGCGGGCATGCCTGTTCGAGCGTCATTTCAACCCTCAAGCTCAGCTTGGTG  340
             ||||||||||||||||||||||||||||||||||||||||||||||||||||||||||||
Ref 313      CAGTATTCTGGCGGGCATGCCTGTTCGAGCGTCATTTCAACCCTCAAGCTCAGCTTGGTG  372

Qry 341      TTGGGACTCGCGGTAACCCGCGTTCCCCAAATCGATTGGCGGTCACGTCGAGCTTCCATA  400
             ||||||||||||||||||||||||||||||||||||||||||||||||||||||||||||
Ref 373      TTGGGACTCGCGGTAACCCGCGTTCCCCAAATCGATTGGCGGTCACGTCGAGCTTCCATA  432

Qry 401      GCGTAGTAATCATACACCTCGTTACTGGTAATCGTCGCGGCCACGCCGTAAAACCCCAAC  460
             ||||||||||||||||||||||||||||||||||||||||||||||||||||||||||||
Ref 433      GCGTAGTAATCATACACCTCGTTACTGGTAATCGTCGCGGCCACGCCGTAAAACCCCAAC  492

Qry 461      TTCTGAATGTTGACCTCGGATCAGGTAGGAATAC  494
             ||||||||||||||||||||||||||||||||||
Ref 493      TTCTGAATGTTGACCTCGGATCAGGTAGGAATAC  526
```

Reference sequence:
Fusarium incarnatum-equiseti species complex (NRRL 34011; MLST type: 15-a)
GQ505723
Fusarium incarnatum-equiseti species complex (NRRL 34011; MLST type: 15-a), Fusarium incarnatum-equiseti species complex (NRRL 34011; MLST type: 15-a), 15-a, USA TX, Human sputum, n7: 28S ribosomal RNA large subunit (28S - LSU)

Sequence length: 1132

Similarity: 24/24 [100.000 %], Gaps: 0 [0.000 %], Coverage: 24/24 [100.000 %]
Score: 39.6241, Probability: 0.00194015, Direction: +/+

```
Qry 2        GCGGAGGGATCATTACCGAGTTTA  25
             ||||||||||||||||||||||||
Ref 29       GCGGAGGGATCATTACCGAGTTTA  52
```

50	Fusarium incarnatum-equiseti species complex (NRRL 34011; MLST type: 15-a) GQ505723 Fusarium incarnatum-equiseti species complex (NRRL 34011; MLST type: 15-a), Fusarium incarnatum-equiseti species complex (NRRL 34011; MLST type: 15-a), 15-a, USA TX, Human sputum, n4: Internal transcribed spacers (ITS1 and ITS2)	717.988	0	99.791	2	96.761	+/+	***

Alignment

Reference sequence:
Fusarium incarnatum-equiseti species complex (NRRL 34011; MLST type: 15-a)

GQ505723
Fusarium incarnatum-equiseti species complex (NRRL 34011; MLST type: 15-a), Fusarium incarnatum-equiseti species complex (NRRL 34011; MLST type: 15-a), 15-a, USA TX, Human sputum, n4: Internal transcribed spacers (ITS1 and ITS2)

Sequence length: 1132

Similarity: 453/454 [99.780 %], Gaps: 0 [0.000 %], Coverage: 454/454 [100.000 %]
Score: 717.988, Probability: 0, Direction: +/+

```
Qry 41         ACATACCTATACGTTGCCTCGGCGGATCAGCCCGCGCCCCGTAAAAAGGGACGGCCCGCC  100
               |||||||||||||||||||||||||||||||||||||||||||||| |||||||||||||
Ref 73         ACATACCTATACGTTGCCTCGGCGGATCAGCCCGCGCCCCGTAAAACGGGACGGCCCGCC  132

Qry 101        CGAGGACCCCTAAACTCTGTTTTTAGTGGAACTTCTGAGTAAAACAAACAAATAAATCAA  160
               ||||||||||||||||||||||||||||||||||||||||||||||||||||||||||||
Ref 133        CGAGGACCCCTAAACTCTGTTTTTAGTGGAACTTCTGAGTAAAACAAACAAATAAATCAA  192

Qry 161        AACTTTCAACAACGGATCTCTTGGTTCTGGCATCGATGAAGAACGCAGCAAAATGCGATA  220
               ||||||||||||||||||||||||||||||||||||||||||||||||||||||||||||
Ref 193        AACTTTCAACAACGGATCTCTTGGTTCTGGCATCGATGAAGAACGCAGCAAAATGCGATA  252

Qry 221        AGTAATGTGAATTGCAGAATTCAGTGAATCATCGAATCTTTGAACGCACATTGCGCCCGC  280
               ||||||||||||||||||||||||||||||||||||||||||||||||||||||||||||
Ref 253        AGTAATGTGAATTGCAGAATTCAGTGAATCATCGAATCTTTGAACGCACATTGCGCCCGC  312

Qry 281        CAGTATTCTGGCGGGCATGCCTGTTCGAGCGTCATTTCAACCCTCAAGCTCAGCTTGGTG  340
               ||||||||||||||||||||||||||||||||||||||||||||||||||||||||||||
Ref 313        CAGTATTCTGGCGGGCATGCCTGTTCGAGCGTCATTTCAACCCTCAAGCTCAGCTTGGTG  372

Qry 341        TTGGGACTCGCGGTAACCCGCGTTCCCCAAATCGATTGGCGGTCACGTCGAGCTTCCATA  400
               ||||||||||||||||||||||||||||||||||||||||||||||||||||||||||||
Ref 373        TTGGGACTCGCGGTAACCCGCGTTCCCCAAATCGATTGGCGGTCACGTCGAGCTTCCATA  432

Qry 401        GCGTAGTAATCATACACCTCGTTACTGGTAATCGTCGCGGCCACGCCGTAAAACCCCAAC  460
               ||||||||||||||||||||||||||||||||||||||||||||||||||||||||||||
Ref 433        GCGTAGTAATCATACACCTCGTTACTGGTAATCGTCGCGGCCACGCCGTAAAACCCCAAC  492

Qry 461        TTCTGAATGTTGACCTCGGATCAGGTAGGAATAC  494
               ||||||||||||||||||||||||||||||||||
Ref 493        TTCTGAATGTTGACCTCGGATCAGGTAGGAATAC  526
```

Reference sequence:
Fusarium incarnatum-equiseti species complex (NRRL 34011; MLST type: 15-a)
GQ505723
Fusarium incarnatum-equiseti species complex (NRRL 34011; MLST type: 15-a), Fusarium incarnatum-equiseti species complex (NRRL 34011; MLST type: 15-a), 15-a, USA TX, Human sputum, n4: Internal transcribed spacers (ITS1 and ITS2)

Sequence length: 1132

Similarity: 24/24 [100.000 %], Gaps: 0 [0.000 %], Coverage: 24/24 [100.000 %]
Score: 39.6241, Probability: 0.00194015, Direction: +/+

```
Qry 2          GCGGAGGGATCATTACCGAGTTTA  25
               ||||||||||||||||||||||||
Ref 29         GCGGAGGGATCATTACCGAGTTTA  52
```
